# High-fat stimulation induces atrial neural remodeling by reducing NO production via the CRIF1/eNOS/P21 axi

**DOI:** 10.1186/s12944-023-01952-7

**Published:** 2023-11-06

**Authors:** An Zhang, Huilin Li, Qiyuan Song, Yansong Cui, Yujiao Zhang, Ximin Wang, Zhan Li, Yinglong Hou

**Affiliations:** 1 Department of Cardiology, Shandong Provincial Qianfoshan Hospital, Shandong Medicine and Health Key Laboratory of Cardiac Electrophysiology and Arrhythmia, The First Affiliated Hospital of Shandong First Medical University, Shandong, China; 2 Department of Cardiology, Cheeloo College of Medicine, Shandong Provincial Qianfoshan Hospital, Shandong University, Jinan, China; 3 Department of Emergency Medicine, The First Affiliated Hospital of Shandong First Medical University & Shandong Provincial Qianfoshan Hospital, Shandong, China

**Keywords:** Atrial fibrillation, High-fat, Nitric oxide, Nitrosylation, Neural remodelling

## Abstract

**Background:**

Autonomic remodeling of the atria plays a pivotal role in the development of atrial fibrillation (AF) and exerts a substantial influence on the progression of this condition. Hyperlipidemia is a predisposing factor for AF, but its effect on atrial nerve remodeling is unclear. The primary goal of this study was to explore the possible mechanisms through which the consumption of a high-fat diet (HFD) induces remodeling of atrial nerves, and to identify novel targets for clinical intervention.

**Methods:**

Cell models were created in vitro by subjecting cells to palmitic acid (PA), while rat models were established by feeding them a high-fat diet. To investigate the interplay between cardiomyocytes and nerve cells in a co-culture system, we utilized Transwell cell culture plates featuring a pore size of 0.4 μm. The CCK-8 assay was employed to determine cell viability, fluorescent probe DCFH-DA and flow cytometry were utilized for measuring ROS levels, JC-1 was used to assess the mitochondrial membrane potential, the Griess method was employed to measure the nitric oxide (NO) level in the supernatant, a fluorescence-based method was used to measure ATP levels, and MitoTracker was utilized for assessing mitochondrial morphology. The expression of pertinent proteins was evaluated using western blotting (WB) and immunohistochemistry techniques. SNAP was used to treat nerve cells in order to replicate a high-NO atmosphere, and the level of nitroso was assessed using the iodoTMT reagent labeling method.

**Results:**

The study found that cardiomyocytes’ mitochondrial morphology and function were impaired under high-fat stimulation, affecting nitric oxide (NO) production through the CRIF1/SIRT1/eNOS axis. In a coculture model, overexpression of eNOS in cardiomyocytes increased NO expression. Moreover, the increased Keap1 nitrosylation within neuronal cells facilitated the entry of Nrf2 into the nucleus, resulting in an augmentation of P21 transcription and a suppression of proliferation. Atrial neural remodeling occurred in the HFD rat model and was ameliorated by increasing myocardial tissue eNOS protein expression with trimetazidine (TMZ).

**Conclusions:**

Neural remodeling is triggered by high-fat stimulation, which decreases the production of NO through the CRIF1/eNOS/P21 axis. Additionally, TMZ prevents neural remodeling and reduces the occurrence of AF by enhancing eNOS expression.

**Supplementary Information:**

The online version contains supplementary material available at 10.1186/s12944-023-01952-7.

## Introduction

Atrial fibrillation (AF) is the prevailing clinical irregular heartbeat [1], and in recent times, its frequency has been progressively rising annually [2]. AF is marked by a high occurrence of other medical conditions and an elevated likelihood of mortality, yet the factors causing it are not thoroughly comprehended. The main pathogenesis of AF includes electrical, structural, neural, and metabolic remodeling [3]. Certain research has indicated that neural restructuring plays a crucial part in the growth and sustenance of AF. Neurological remodeling includes the overproliferation of nerves, heterogeneity of distribution, and enhanced activity. Hyperlipidemia is an elevation of lipids in the blood due to abnormal metabolic function [4]. Hyperlipidemia can affect cardiac function in several ways, and it is well-known to cause atherosclerosis. Research has indicated that hyperlipidemia has the potential to cause oxidative stress and trigger proinflammatory reactions, resulting in the disturbance of myocardial homeostasis [5]. Myocardial remodeling and heightened vulnerability to atrial fibrillation occur due to mitochondrial damage, mast cell activation, and subsequent degranulation [6]. In excess, reactive oxygen species (ROS) can harm mitochondria [7, 8]and disturb the organism’s redox balance. Evidence shows that excessive lipids can cause oxidative stress that increases the risk of AF [9]. Nitric oxide (NO) can also be directly reduced when ROS levels are elevated [10]. NO is a gas that acts as a signaling molecule and is produced by nitric oxide synthase (NOS) [11]. It is involved in regulating cell growth, metabolism, and exerting anti-inflammatory effects. Regulating cardiovascular homeostasis is a crucial element. Furthermore, NO groups have the ability to covalently alter cysteine thiols, resulting in the production of S-nitrosylated thiols (SNO) [12], which is referred to as S-nitrosylation, in addition to activating the conventional cyclic guanosine monophosphate (cGMP)-dependent signaling pathway. In persistent AF animal models, reduced eNOS expression and decreased circulating NO levels have been demonstrated [13, 14]. Under normal physiological conditions, NO inhibits the proliferation of neuronal cells [15]. Some studies have confirmed that increasing atrial muscle NO content in dogs with AF can inhibit autonomic remodeling, but the exact mechanism is unknown [16].

Our prior research discovered that the stimulation of a diet rich in fats could enhance the development of atrial fibrillation caused by myocardial fibrosis through the initiation of programmed cell death in the heart muscle cells. In order to explore the potential mechanisms through which high-fat conditions contribute to the progression of AF, the present study hypothesizes that high-fat stimulation induces atrial fibrillation (AF) by disrupting the balance of nitric oxide (NO) and promoting atrial nerve remodeling through the CRIF1/eNOS/P21 axis via mitochondrial damage.

## Materials and methods

### Cell culture

Fu Heng Biotechnology (Shanghai, China) provided rat cardiomyocytes (H9C2) and rat adrenal pheochromocytoma cells (PC12). Cell lines were cultured in DMEM (Invitrogen, Waltham, USA) supplemented with 10% FBS at 37 °C under 5% CO2. Sigma (St. Louis, MO) was where the purchase of Palmitic acid (PA) took place. PA was dissolved in BSA before use. When only BSA was added to the cells as a control, it was labeled as ‘VEH’, while in all other cases, it was labeled as ‘CON’. Unless otherwise noted, all drugs used in this experiment were purchased from MCE.

### Cell viability assay

Once the cell model was built, we added 10µL of Cell Counting Kit-8 (CCK-8) solution to every well. This was followed by a 2-hour incubation period in a cell incubator. Subsequently, we assessed the absorbance at 450 nm.

### Western blotting

The lysate mixture was made by combining RIPA lysis buffer and PMSF (Beyotime, Shanghai, China). Next, the lysate mixture was introduced to the samples, subjected to lysis on ice, and then centrifuged at a speed of 12,000 revolutions per minute for 5 min at a temperature of four degrees. The liquid above the sediment was combined with 5× SDS-PAGE sample loading buffer and incubated for 10 min at 95 °C in a metal bath before being frozen at -20 °C. Proteins were isolated by employing SDS‒PAGE (EpiZyme, Shanghai, China) on a 15*15 gel containing 15 µg protein in each lane. The proteins were transferred onto PVDF membranes, followed by a 1-hour blocking step using skimmed milk powder. Afterwards, the primary antibody (Table 1) was incubated with them overnight at a temperature of 4 °C. The membrane was subjected to three successive 5-minute rinses with TBST, then incubated for 1 h with horseradish enzyme-conjugated goat anti-mouse/rabbit IgG (H + L) (ZSGB-BIO, China). Subsequently, it underwent an additional series of three 5-minute TBST rinses. The membrane was coated with a uniform layer of ECL luminescent solution obtained from Millipore in Germany, and then it was exposed.

**Table 1:** List of all antibodies used in the study

Name	Article number	Dilution ratio	Company
β-actin	3700	1:1000	CST
CHAT	ab13786	1:1000	Abcam
eNOS	ab199956	1:1000	Abcam
Keap1	10503-2-AP	1:2000	Proteintech
Mfn1	ab221661	1:1000	Abcam
Mfn2	12186-1-AP	1:2000	Proteintech
MTCO1	ab203912	1:1000	Abcam
NDUFB8	ab192878	1:1000	Abcam
Nrf2	ab109199	1:1000	Abcam
PGP9.5	ab108986	1:1000	Abcam
SDHA	ab137040	1:1000	Abcam
SIRT1	9475	1:1000	CST
TH	sc25269	1:1000	Santa Cruz
UQCRC2	ab203832	1:1000	Abcam

### ROS and fluorescence measurement

The ROS fluorescent probe (DCFH-DA) was diluted 1:2000 in DMEM. Then, the medium was aspirated and discarded. The probe was introduced and left to incubate for 30 min at a temperature of 37 degrees Celsius. The probe was then discarded, and DMEM was used three times to wash the cells. Next, the fluorescence intensity was measured using a Leica fluorescence microscope with an excitation wavelength of 488 mm. Establish a suitable threshold for fluorescence intensity and compare the ratio of cells exhibiting a positive signal at the designated threshold to the overall cell count.

### JC-1 assay

The JC-1 assay was performed using the JC-1 kit (Beyotime, Shanghai, China). The JC-1 working solution was diluted 1:200 with JC-1 buffer. Following incubation, the liquid above the sediment was decanted, and the cells were subsequently washed three times with JC-1 buffer. Next, a sufficient amount of medium was introduced, and the cells were examined using a fluorescence microscope (Leica, Wetzlar, Germany). It was detected using a 490 nm excitation wavelength and a 530 nm emission wavelength for JC-1 monomers, and a 525 nm excitation wavelength and a 590 nm emission wavelength for JC-1 aggregates.

### ATP detection

The cells were lysed on ice using the improved ATP assay kit (Beyotime, Shanghai, China), and then centrifuged at 12,000×g for 5 min at 4 °C. Afterwards, the liquid above was combined with the ATP assay solution, and the resulting intensity of chemiluminescence (measured in RLU) was documented using a chemiluminescence device.

### NO content measurement

The cells were lysed on ice using the improved ATP assay kit (Beyotime, Shanghai, China), then centrifuged at 12,000 × g for 5 min at 4 °C. Afterwards, the liquid above was combined with the ATP assay solution, and the resulting intensity of chemiluminescence (measured in RLU) was documented using a chemiluminescence device.

### Cell transfection

Transfection was performed when the cell confluence was 60–70%. Opti-MEM (Gibco, NY, USA) was used to replace the culture medium. Lipo2000 (Invitrogen, Waltham, USA) was mixed with plasmid or siRNA (GenePharma, Shanghai, China). The blend was slowly introduced into the cells and subsequently replaced with a complete medium for 4–6 h.

### Expression of eNOS acetylation

Using the immunoprecipitation technique, we observed the presence of eNOS acetylation. The precipitation of proteins was achieved by utilizing an anti-eNOS antibody (Santa Cruz, Cat#sc-376,751) that was cross-linked to Protein A/G magnetic beads (MCE, HY-K0202). The proteins were detected through Western blotting using an anti-acetyl-lysine antibody (CST, Cat#9441).

### Immunofluorescence

After removing the medium, the cells were rinsed three times with PBS. Afterwards, we introduced 4% paraformaldehyde into the mixture and allowed it to react for 15 min. Afterward, the cells underwent another PBS wash, and a permeabilization buffer was applied for 10 min. After another wash, the cells were incubated with a blocking buffer for 10 min.

After incubating overnight at 4 °C, the primary antibody was removed by washing the next day. Next, the luminescent secondary antibody was introduced and left to incubate for 1.5 h in a lightless environment. This was followed by DAPI staining (Beyotime, Shanghai, China). Subsequently, an anti-fluorescence quenching reagent was employed to encapsulate the sample, followed by examination of the outcomes using a confocal microscope (Nikon, Tokyo, Japan). The antibodies utilized included Nrf2 (Proteintech, Cat#16396-1-AP).

### Nitrosylation assay

Nitrosylation was detected using the S-nitrosylation assay kit (Thermo, 90,105). The protein was enriched using a Keap1 antibody (Proteintech, Cat#10503-2-AP) cross-linked with magnetic beads. Next, the protein was extracted, and the protein sample concentration was adjusted to 1 mg/ml using lysate. Next, half of the total was combined with five times the loading buffer and heated at 95 °C for 10 min. The other half was added to a corresponding volume of MMTS and vigorously shaken to block free sulfhydryl groups, precipitate the protein and remove excess MMTS. It was then resuspended using HENS buffer, and sodium ascorbate was added to reduce S-nitrosocysteine. Then, it was labeled with iodoTMT reagent, and Western blot detection was performed using an anti-TMT antibody. The control group will have the addition of corresponding DMSO, labeled as VEH.

### EdU

We performed EdU detection using the kit purchased from Beyotime(Cat# C0075S). After completing the stimulation, an equal volume of the EdU reaction solution was introduced, and the cells were subsequently incubated at 37 °C for 2 h. The reaction solution was prepared as per the manufacturer’s instructions and added after washing and fixation, followed by a light-protected incubation for 20 min. Cell nuclei were labeled with Hoechst staining after washing with PBS. Fluorescence microscopy was utilized for detection, with a maximum excitation wavelength of 346 nm and a maximum emission wavelength of 565 nm.

### Animal grouping and modeling

Male Sprague‒Dawley (SD) rats, aged eight weeks and weighing approximately 200 g (± 20 g), were acquired from Viton Lever Laboratory Animal Technology Co. The rodents were kept in a chamber where the temperature was controlled at (20 ± 2) °C and exposed to a 12-hour period of light followed by a 12-hour period of darkness. A total of thirty rats were divided into three groups: control (n = 10), HFD(n = 10), and HFD + TMZ (n = 10). Throughout the 12-week study duration, the control group was provided with a standard diet. Following an eight-week period of high-fat diet (HFD) consumption, TMZ (MCE, HYB0968A) was given via intraperitoneal injection at a daily dose of 7 mg/kg for four weeks. 30% of the calories from fat were derived from palm oil.

### Programmed electrical stimulation

Through the right jugular vein, electrodes were inserted into the high right atrium. S1S2 electrical stimulation was administered using a multichannel electrical stimulator with a frequency ratio of 8:1, an S1S1 interval of 120ms, and an S1S2 initial interval of 60ms, decreasing by 2ms each time. The longest interval between S1 and S2 that could not induce atrial stimulation was known as the atrial effective refractory period (AERP). A fast, irregular atrial rate lasting at least 2s was considered successfully inducing AF.

### Measurement of biochemical blood indexes

Blood was collected from the apical part of the heart after the animals were anesthetized. After being spun at a speed of 2000 revolutions per minute for 20 min at ambient temperature, the serum was subsequently preserved at a temperature of -80 °C. Cholesterol levels, including HDL, LDL, and total cholesterol, were assessed with a fully automated biochemical analyzer from Roche in Switzerland. Additionally, triglycerides and malondialdehyde (MDA) levels were measured using a commercial kit provided by Elabscience in Wuhan, China.

### Immunohistochemistry

Following the collection of tissue, the samples were immersed in 4% paraformaldehyde for 48 h. Paraffin wax was used to embed them, which was then cut into sections of 5 μm. These sections underwent a gradient dewaxing and antigen retrieval using a citric acid solution. The activity of endogenous peroxidase was inhibited by applying 3% hydrogen peroxide for 15 min. Following rinsing, the sections were obstructed using a 3% BSA solution for 30 min. Subsequently, they were incubated with the primary antibody at 4 °C overnight. The next day, paraffin sections were brought to room temperature for 30 min and rinsed with PBS three times, each lasting 5 min. Afterwards, the second antibody was added gradually and left to incubate at ambient temperature for 50 min. After washing, DAB was added dropwise, and the reaction was terminated by microscopic control of color development time and rinsing after the appearance of positivity. Hematoxylin staining was rinsed after 1 min, and the hematoxylin differentiation solution was applied for several seconds. After dehydration and sealing, microscopic observation was performed, and data were collected. The following antibodies were used: PGP9.5(1:200, Cat# ab108986, Abcam), TH (1:200, Cat# sc25269, Santa Cruz), CHAT (1:200, Cat# ab137869, Abcam).

### Statistical methods

Each experiment was conducted with triplicate independent samples. Statistical analysis was performed using unpaired t-tests for comparisons between two groups, and one-way ANOVA was employed for pairwise comparisons among three or more groups. GraphPad Pro 9.0, located in San Diego, CA, was utilized for the statistical analyses. The mean ± SD values were used to present the results, and statistical significance was determined at a threshold of *P* < 0.05. In all figures, the symbols indicate statistical significance levels as follows: * = *P* < 0.05, ** = *P* < 0.01, *** = *P* < 0.001, and **** = *P* < 0.0001.

## Results

### PA increases ROS in cardiomyocytes

**Fig. 1:**
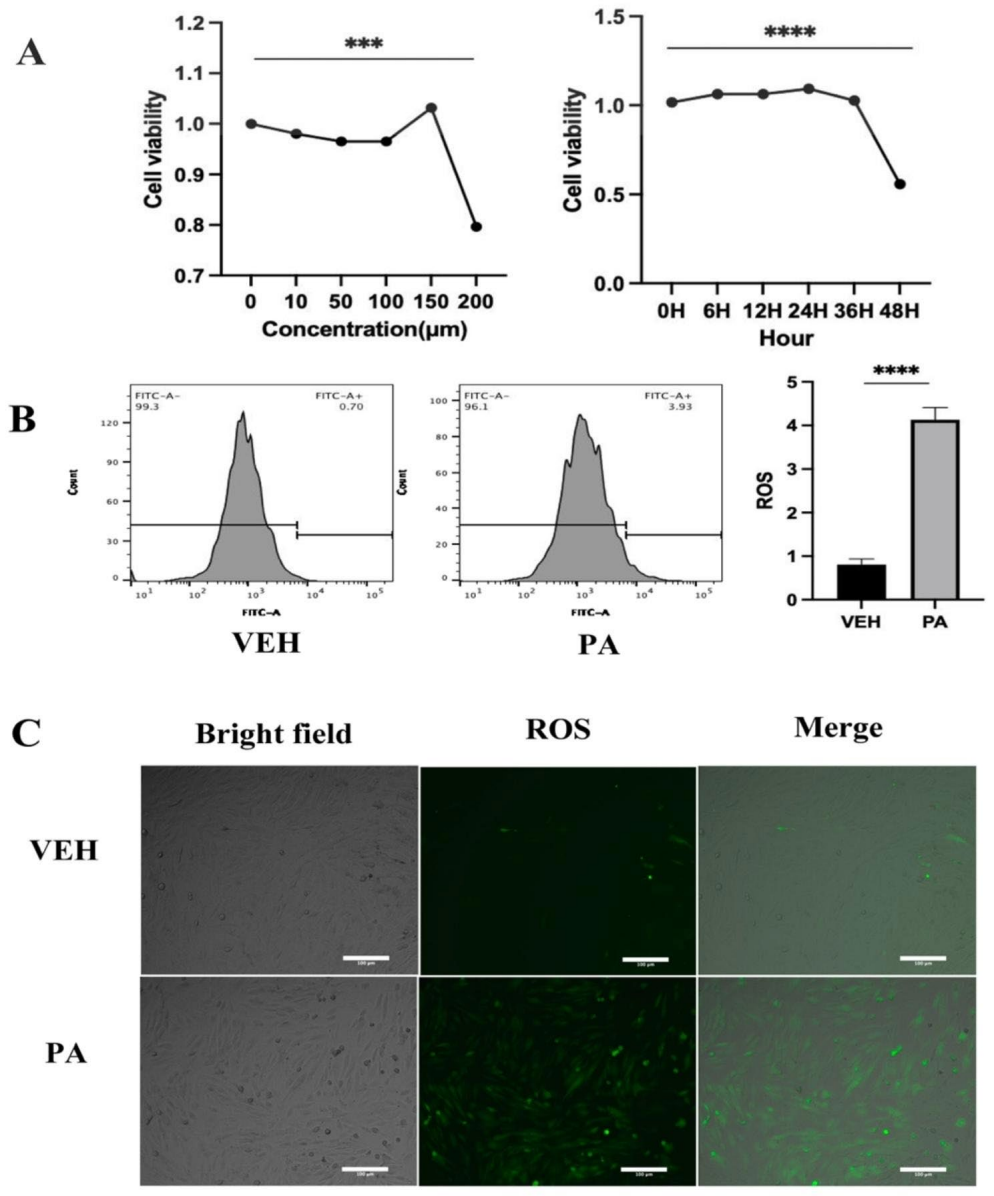
PA increases ROS levels in cardiomyocytes. (**A**) The CCK-8 assay was conducted following stimulation with PA at concentrations of 0, 10, 50, 100, 150, or 200 µM, or after 150 µM PA stimulation for durations of 0, 6, 12, 24, 36, or 48 h. (**B**) Flow cytometry was employed to measure ROS levels following PA stimulation. (**C**) ROS production following PA stimulation was detected using fluorescence microscopy(20X). Scale bar = 100 μm

The CCK-8 method was used to evaluate cell viability after stimulating H9C2 cells with various concentrations of PA (ranging from 0 to 200µM). There was no notable disparity in cell viability when exposed to PA concentrations ranging from 0 to 150µM, but a decline in cell viability was observed at a PA concentration of 200µM. For the time-dependent cell viability assay, we chose 150µM. There was no notable variation in cell viability within 36 h, but a decline in cell viability was observed after 48 h (Fig. 1A). Therefore, we used a PA concentration of 150µM and a stimulation time of 36 h for subsequent experiments. We used the DCFH-DA fluorescent probe to detect intracellular ROS production after PA stimulation and found that PA stimulation increased intracellular ROS production by flow cytometry and cytofluorimetry (Fig. 1B **and C**).

### PA impaired mitochondrial morphology and function

**Fig. 2:**
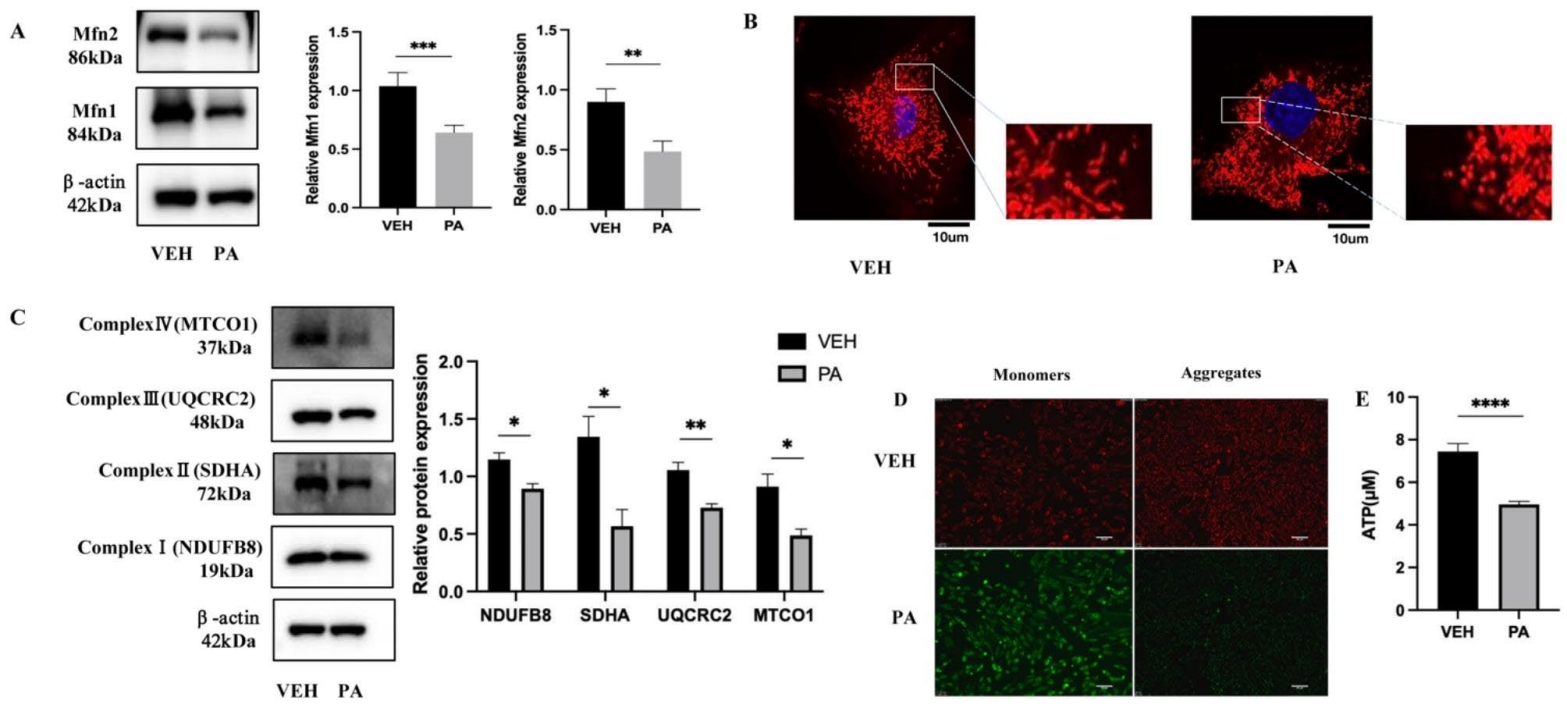
PA impairs mitochondrial morphology and function. (**A**) WB detection of Mfn1 and Mfn2 protein expression after PA stimulation. After PA stimulation, MitoTracker Red staining revealed the morphology of the mitochondria(100X). Scale bar = 10 μm. (**C**) WB detection of the protein expression of mitochondrial respiratory chain complexes after PA stimulation. JC-1 detected the mitochondrial membrane potential following stimulation with PA (20X). Scale bar = 50 μm. (**E**) Myocardial ATP content after PA stimulation

ROS are mainly produced by mitochondria. Thus, we applied MitoTracker to label mitochondria, which typically exhibit an elongated and thread-like morphology under normal conditions. After stimulation with 150µM PA for 36 h, mitochondrial fracture increased, and fragmentation became more apparent (Fig. 2B). Western blotting showed that mitofusin1(Mfn1) and mitofusin 2(Mfn2) protein expression decreased (Fig. 2A). These findings indicate that PA stimulation can induce alterations in mitochondrial fusion morphology and increase the proportion of damaged mitochondria. To further verify the function of mitochondria, Western blotting was used to detect decreased mitochondrial complex I, II, III, and IV protein expression (Fig. 2C). JC-1 staining showed reduced mitochondrial membrane potential (Fig. 2D) and decreased ATP levels (Fig. 2E). These results indicate that mitochondrial function was impaired.

### PA-stimulated generation of ROS regulates SIRT1/eNOS expression

**Fig. 3:**
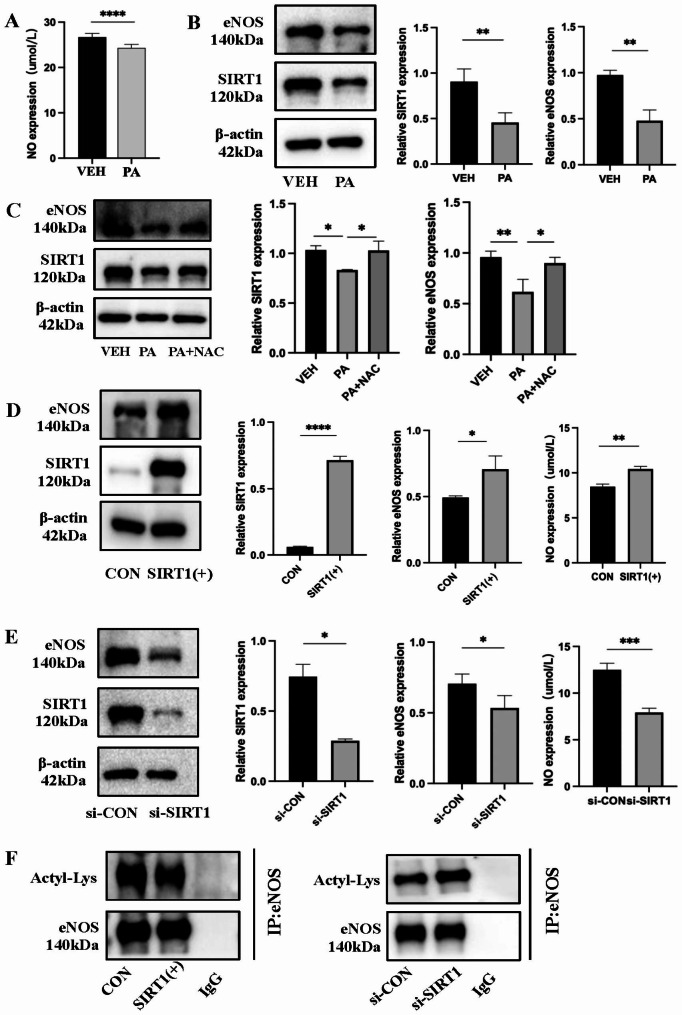
PA-stimulated generation of ROS regulates SIRT1/eNOS expression. (**A**) NO content in the supernatant after PA stimulation. Detection of SIRT1 and eNOS protein levels after PA stimulation using WB. Detection of SIRT1 and eNOS protein levels was performed using WB after scavenging ROS with NAC (5 mM). (**D**) After overexpressing SIRT1, the levels of SIRT1 and eNOS proteins were detected, along with the measurement of NO levels in the supernatant. After silencing SIRT1, an analysis was conducted on the levels of SIRT1 and eNOS proteins, and the levels of NO in the supernatant. (**F**) Levels of eNOS acetylation after overexpression of SIRT1 or silencing of SIRT1

Under stimulation with 150 µM PA for 36 h, the NO content in the cell supernatant decreased (Fig. 3A), and a decrease was observed in the expression of sirtuin1 (SIRT1) and endothelial nitric oxide synthase (eNOS) proteins (Fig. 3B). After using NAC to scavenge ROS, the PA-induced downregulation of SIRT1 and eNOS was partially reversed (Fig. 3C). To confirm the correlation between SIRT1 and eNOS, we introduced the SIRT1 overexpression plasmid and siRNA into H9C2 cardiomyocytes. As a result of silencing SIRT1, the expression of eNOS decreased, whereas SIRT1 overexpression led to an increase in eNOS expression (Fig. 3D **and E**). SIRT1, a protein deacetylase dependent on nuclear NAD+, controls protein expression by deacetylating them. Therefore, we also examined the acetylation level of eNOS. Overexpressing SIRT1 decreased eNOS acetylation, whereas silencing SIRT1 resulted in an increase in eNOS acetylation (Fig. 3F).

### PA regulates SIRT1/eNOS expression through CRIF1 and affects NO production

**Fig. 4:**
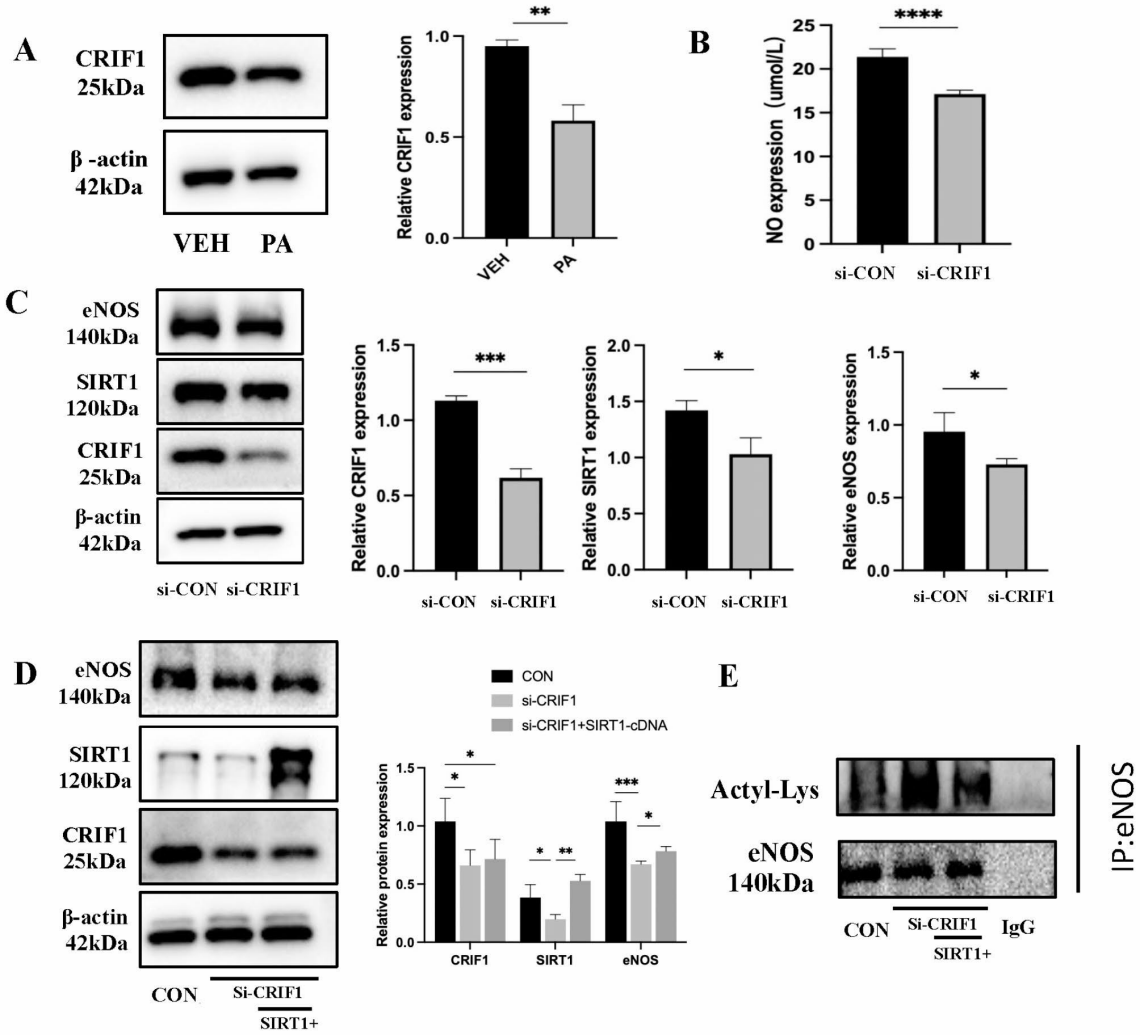
PA regulates SIRT1/eNOS expression through CRIF1 and affects NO production. (**A**) WB detection of CRIF1 protein expression after PA stimulation. (**B**) NO content in the supernatant after silencing CRIF1. (**C**) WB detection of CRIF1, SIRT1, and eNOS protein expression after silencing CRIF1. (**D**) WB detection of CRIF1, SIRT1, and eNOS protein expression after silencing CRIF1 and overexpressing SIRT1 again. (**E**) eNOS acetylation levels after silencing CRIF1 again overexpressing SIRT1

CR6-interacting factor 1 (CRIF1), an essential mitochondrial protein, plays a key role in assembling mitochondrial oxidative phosphorylation complexes. In response to PA stimulation, CRIF1 protein expression was reduced (Fig. 4A). Following the use of siRNA to silence CRIF1, the supernatant exhibited a decline in NO levels, along with a decrease in the protein expression of SIRT1 and eNOS (Fig. 4B **and C**). As a result, our hypothesis was that PA stimulation caused harm to the mitochondria. This decreased CRIF1 expression, reducing cellular NO production through the SIRT1/eNOS axis. To confirm the connection between CRIF1, SIRT1, and eNOS, we proceeded to enhance the expression of SIRT1 following the suppression of CRIF1. Our findings revealed that the increased expression of SIRT1 partially counteracted the reduction in eNOS protein levels resulting from the suppression of CRIF1 (Fig. 4D) and decreased the acetylation level of eNOS (Fig. 4E).

### PA induces PC12 cell proliferation in a coculture model of H9C2 cardiomyocytes and PC12 neuronal cells

**Fig. 5:**
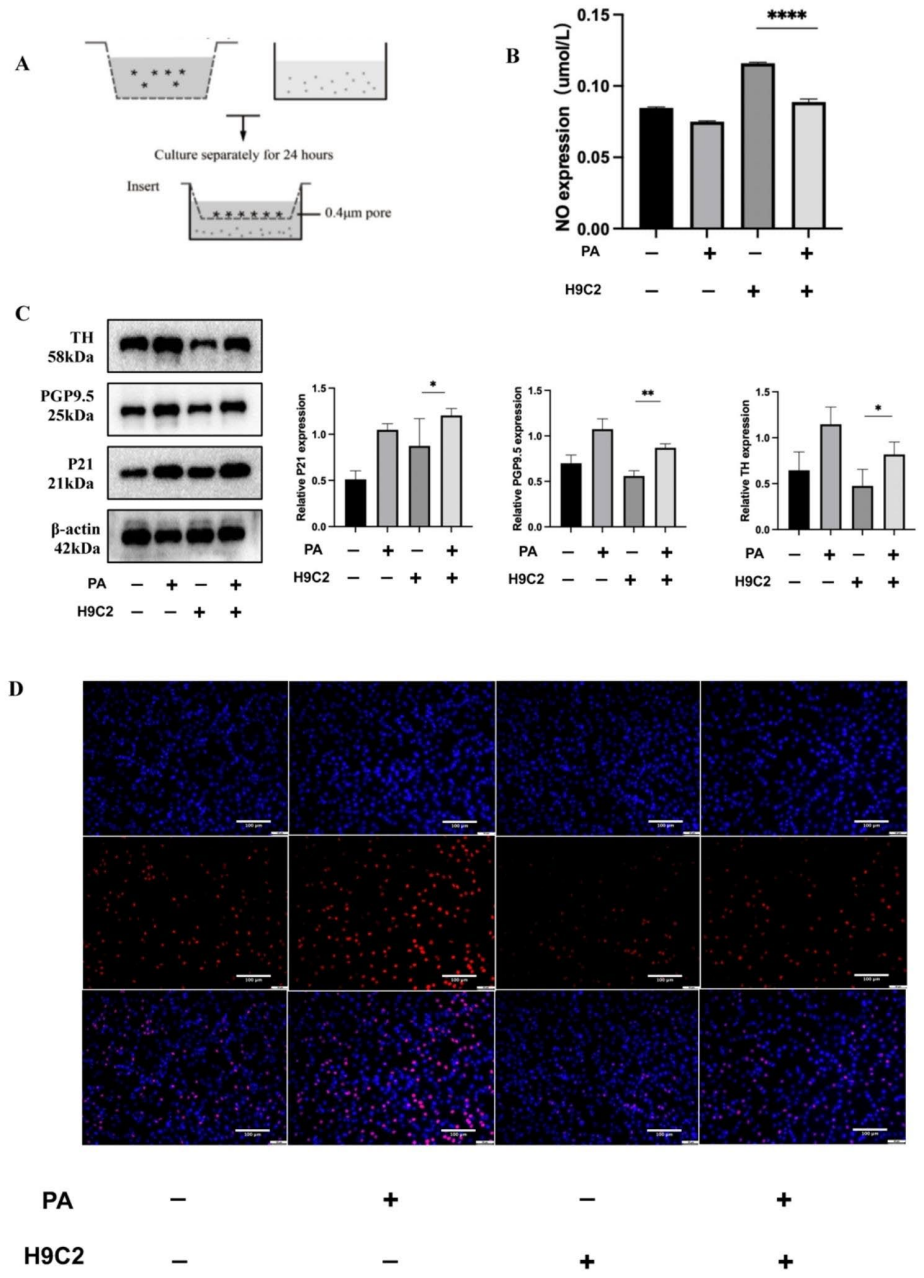
PA induces PC12 cell proliferation in a coculture model of H9C2 cardiomyocytes and PC12 neuronal cells. (**A**) Schematic diagram of the coculture model. (**B**) NO content in the supernatant of the coculture model after PA stimulation. (**C**) WB detection of P21, PGP9.5, and TH protein expression in lower PC12 neuronal cells in the coculture model after PA stimulation. (**D**) EdU assay of proliferation levels of lower layer PC12 neuronal cells in the coculture model after PA stimulation (20X). Scale bar = 100 μm

In order to evaluate the influence of cardiomyocytes on neuronal cells stimulated by PA, we established a coculture model by culturing H9C2 cells and PC12 cells together in a Transwell system (Fig. 5A). Following the addition of PA, there was a reduction in the NO content found in the supernatant (Fig. 5B), while the protein expression of TH and PGP9.5 increased in PC-12 cells. Furthermore, the expression of the P21 protein was observed to rise (Fig. 5C), and the EdU assay indicated an augmentation in the percentage of PC12 cells undergoing proliferation (Fig. 5D).

### Overexpression of eNOS in cardiomyocytes increased the NO content and inhibited the proliferation of lower neuronal cells

**Fig. 6:**
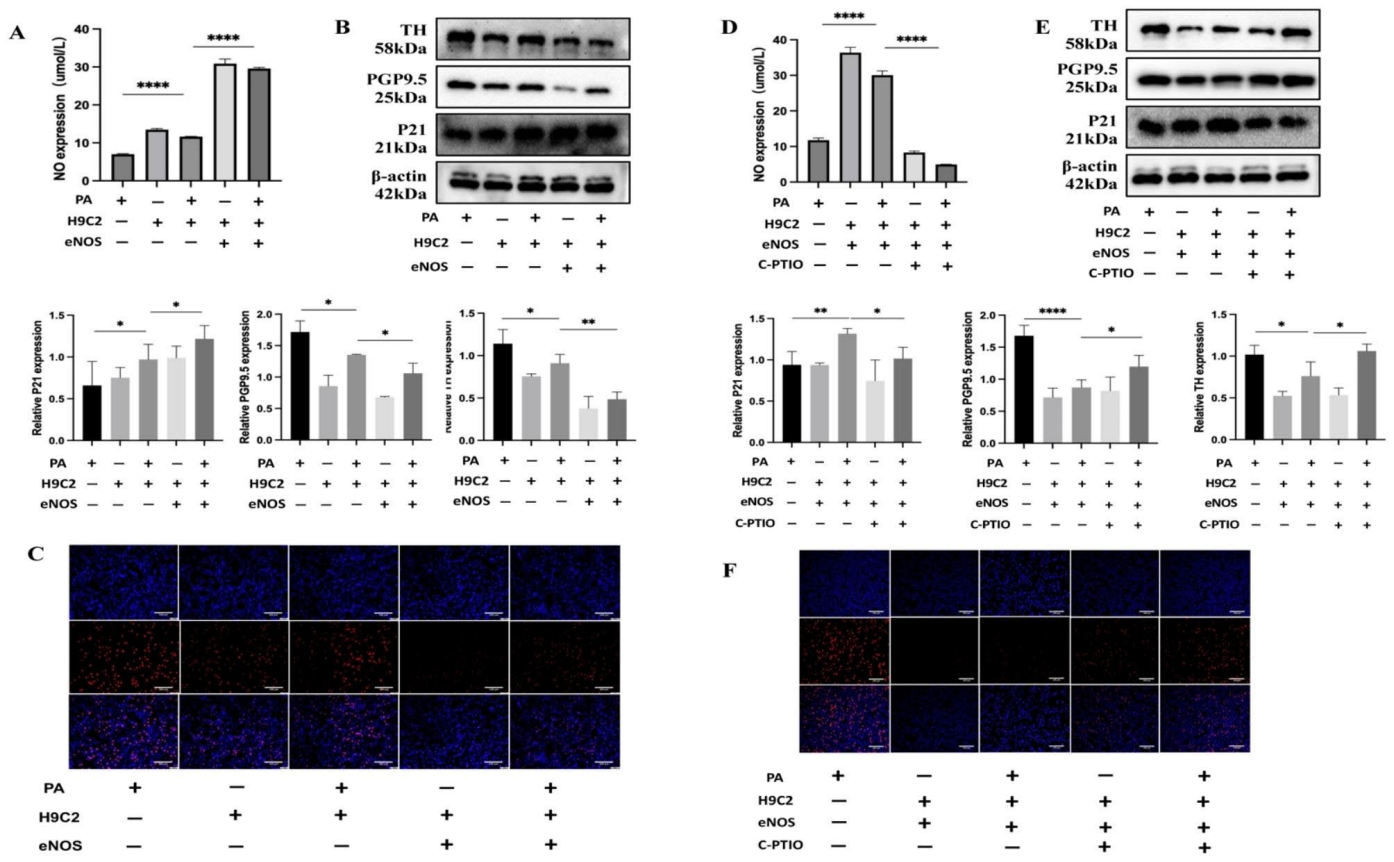
Overexpression of eNOS in cardiomyocytes increased NO content and inhibited the proliferation of lower neuronal cells. (**A**) NO content in the supernatant of the coculture model after PA stimulation in the upper cardiomyocytes overexpressing eNOS. (**B**) WB detection of P21, PGP9.5, and TH protein expression in lower layer PC12 neurons after PA stimulation in upper layer cardiomyocytes overexpressing eNOS. (**C**) EdU assay of proliferation levels of lower layer PC12 neuronal cells after PA stimulation in an upper layer cardiomyocyte coculture model overexpressing eNOS. Scale bar = 100 μm. (**D**) NO content in the supernatant of the coculture model after PA stimulation in the upper layer of cardiomyocytes overexpressing eNOS following the addition of C-PTIO to remove NO. (**E**, **F**) Following the addition of PA to the coculture system, eNOS was observed to be overexpressed in the upper chamber of H9C2 cells. Afterwards, CPTIO was introduced to eliminate NO, and WB analysis was conducted to measure the protein expression levels of P21, PGP9.5, and TH in PC12 cells located in the lower chamber. PC12 cell proliferation was evaluated using the EdU assay (20X). Scale bar = 100 μm

Following the upregulation of eNOS in the upper layer of H9C2 cardiomyocytes, Supernatant nitric oxide (NO) concentration increased (Fig. 6A). Furthermore, the protein levels of P21 in the neuronal cells located in the lower compartment of the coculture setup exhibited an increase, while the expression of TH and PGP9.5 showed a decline (Fig. 6B). The EdU assay showed a decrease in the proportion of proliferating cells (Fig. 6C). In order to confirm if NO has a suppressive impact on neural remodeling, we introduced a nitric oxide scavenger (carboxy-PTIO) to the upper layer’s supernatant after the overexpression of eNOS, which resulted in a decrease in the NO level (Fig. 6D). Moreover, in the lower chamber’s neuronal cells, the expression of P21 decreased, while the expression of tyrosine hydroxylase (TH) and protein gene product 9.5 (PGP9.5) increased (Fig. 6E). Additionally, the EdU assay indicated an increase in the proportion of proliferating cells (Fig. 6F).

### NO inhibits proliferation by nitrosylating Keap1 to promote Nrf2 entry into the nucleus to agonize P21 transcription

**Fig. 7:**
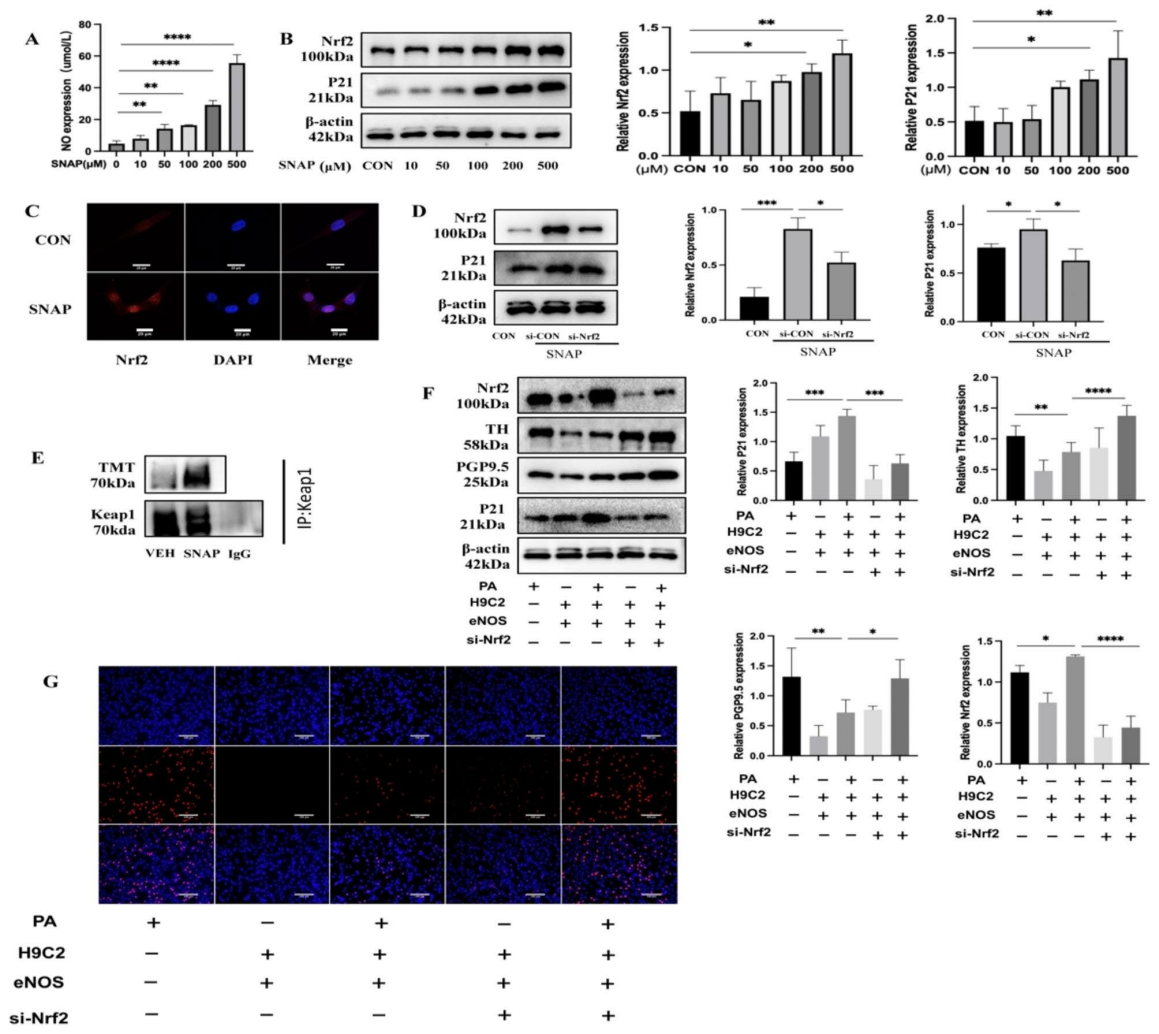
NO inhibits proliferation by nitrosylating Keap1 to promote Nrf2 entry into the nucleus to induce P21 transcription. (**A**, **B**) NO content and protein expression of Nrf2 and P21 in the supernatant after stimulation of PC12 neuronal cells using 0, 10, 50, 100, 200, and 500 µM SNAP. (**C**) Immunofluorescence detection of Nrf2 protein expression after the addition of 500µM SANP. Scale bar = 20 μm. (**D**) Protein expression of Nrf2 and P21 after silencing the expression of Nrf2 after adding 500µM SNAP. (**E**) Detection of nitrosylation of Keap1 after adding 500µM SNAP. (**F**, **G**) Upon PA stimulation, after overexpression of eNOS in upper cardiomyocytes and silencing of Nrf2 in lower neuronal cells, WB detected P21, PGP9.5, and TH protein expression, and EdU showed the proportion of neuronal cell proliferation (20X). Scale bar = 100 μm

We preliminarily demonstrated that NO could promote the protein expression of P21 to inhibit neuronal cell proliferation, but the exact underlying mechanism of this effect remains unknown. Hence, in order to clarify the molecular mechanism through which NO enhances the expression of the P21 protein, we employed the PROMO database (http://alggen.lsi.upc.es/cgi-bin/promo_v3/promo/promoinit.cgi?dirDB=TF_8.3)and the JASPER database(http://jaspar.genereg.net/) for the prediction of potential transcription factors (TFs) that bind to the P21 promoter. The P21 promoter region was predicted to be bound by nuclear factor E2-related factor 2 (Nrf2) with strong affinity, resulting in positive regulation of P21 **(Supplementary Figure A and B)**. We hypothesized that NO regulates the expression of P21 by agonizing Nrf2. We treated PC12 neuronal cells with SNAP (an NO donor) to test this conjecture. SNAP increased the NO concentration in the supernatant (Fig. 7A) in a concentration-dependent manner to activate the expression of Nrf2 and P21 (Fig. 7B). Nrf2 entry into the nucleus was increased after SNAP stimulation (Fig. 7C). Following the addition of SNAP, we suppressed Nrf2 expression by utilizing si-Nrf2, resulting in a decrease in the protein expression of P21 (Fig. 7D). Nrf2 expression is tightly controlled through various mechanisms, with Keap1 serving as the primary regulator. After the introduction of SNAP (500 µM) to PC-12 neuronal cells, we observed a notable rise in the nitrosylation of the Kelch-like ECH-associated protein 1 (Keap1) protein (Fig. 7E). Hence, it can be inferred that NO enhances the nitrosylation of Keap1, preventing Keap1 from degrading Nrf2. As a result, Nrf2 gains access to the nucleus and stimulates the transcription of P21, leading to an antiproliferative impact. In a coculture model, we proceeded to confirm the functions of NO and Nrf2. Following the overexpression of eNOS in the upper stratum of H9C2 cells, si-Nrf2 was administered in the lower stratum of PC-12 cells to suppress the Nrf2 expression. In PC12 cells, the findings indicated a reduction in P21 expression and a rise in TH and PGP9.5 expression (Fig. 7F). EdU showed an increase in the proportion of proliferating neuronal cells (Fig. 7G).

### Trimetazidine inhibits neural remodeling by promoting the protein expression of eNOS

**Fig. 8:**
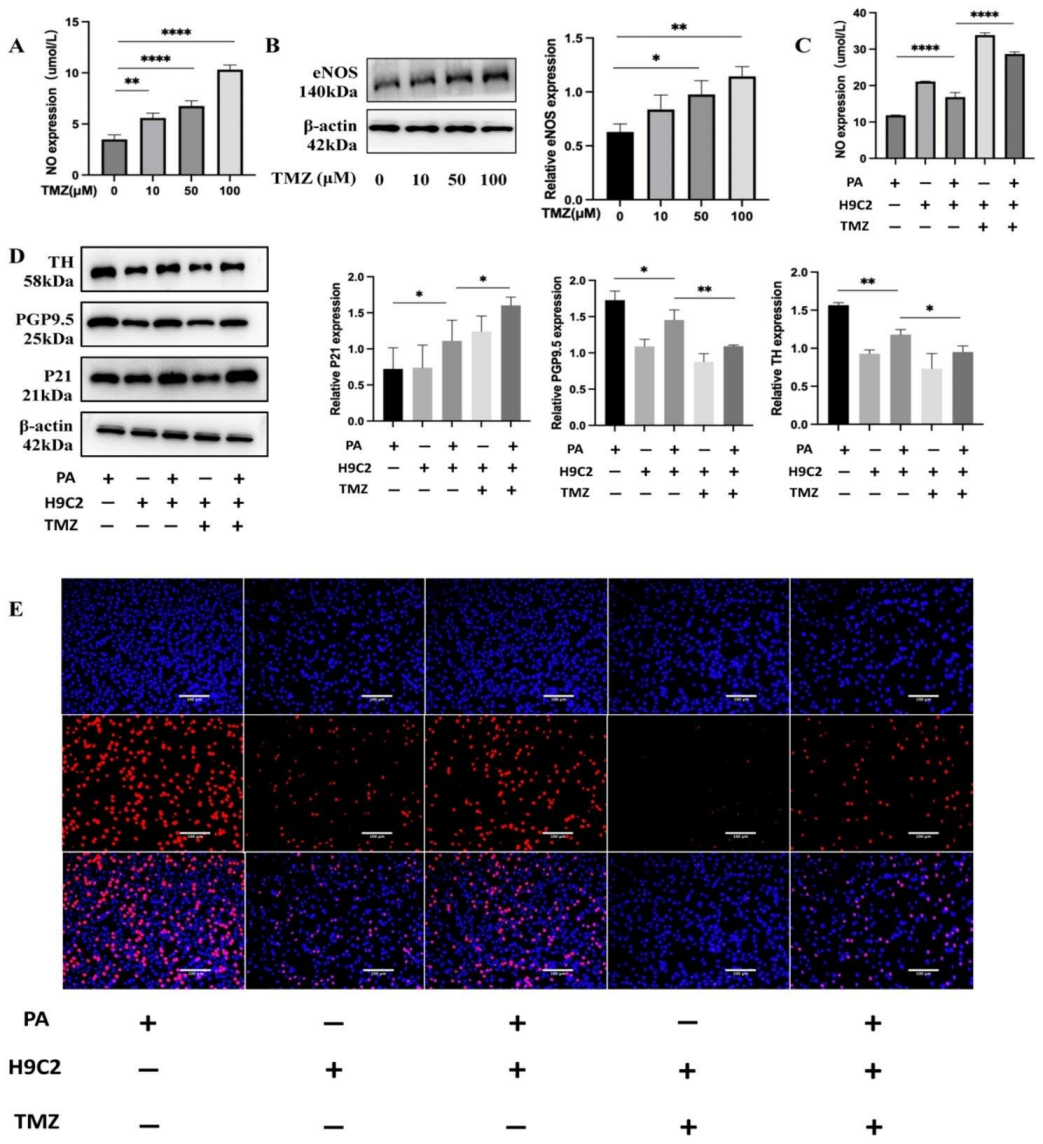
Trimetazidine inhibits neural remodeling by promoting eNOS protein expression. (**A**, **B**) NO content in the supernatant and protein expression of eNOS after stimulation of cardiomyocytes using 0, 10, 50, and 100 µM TMZ. (**C**) NO content in the supernatant after PA stimulation in a culture model with the addition of TMZ. (**D**, **E**) WB detected the protein expression of P21, PGP9.5, and TH in lower-layer PC12 neuronal cells after PA stimulation in a culture model with the addition of TMZ; EdU assay of the proliferation level of lower layer PC12 neuronal cells (20X). Scale bar = 100 μm

We added TMZ to H9C2 cardiomyocytes. TMZ increased the NO content in the supernatant (Fig. 8A) and upregulated the protein expression of eNOS in a concentration-dependent manner (Fig. 8B). In order to confirm the role of TMZ in neural remodeling induced by a high-fat environment, we added TMZ to a high-fat conditioned coculture model. We observed that TMZ increased the NO content in the supernatant (Fig. 8C), increased the expression of P21, and decreased the expression of TH and PGP9.5 in PC12 cells (Fig. 8D). The EdU assay indicated a decrease in the proportion of proliferating cells (Fig. 8E).

### HFD induced atrial neural remodeling and increased the induction rate of AF, and TMZ improved neural remodeling and decreased the induction rate of AF

**Fig. 9:**
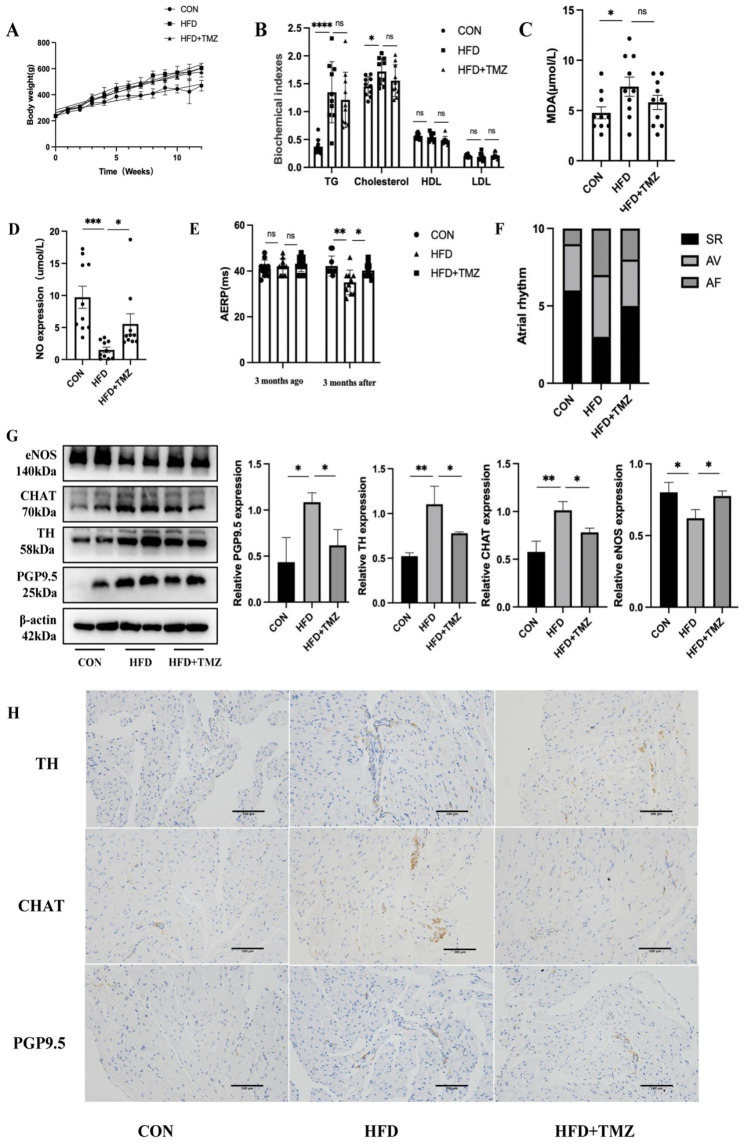
HFD induced atrial neural remodeling and increased the induction rate of AF, and TMZ improved neural remodeling and decreased the induction rate of AF. (**A**) Body weight trends of rats in the CON, HFD, and HFD + TMZ groups. (**B**) Blood biochemical indexes of rats in the CON, HFD, and HFD + TMZ groups. (**C**) MDA levels of rats in the CON, HFD, HFD + TMZ groups. (**D**) NO levels of rats in the CON, HFD, HFD + TMZ groups. (**E**, **F**) Cardiac electrophysiological indexes of the hearts of rats in the CON, HFD, and HFD + TMZ groups. (**G**) WB was used to detect the protein expression of PGP9.5, TH, CHAT, and eNOS in rats in the CON, HFD, and HFD + TMZ groups. (**H**) Immunohistochemistry of PGP9.5, TH, and CHAT in rats of the CON, HFD, and HFD + TMZ groups (20X). Scale bar = 100 μm

In order to validate the neuroprotective effect of TMZ against high-fat-induced neural remodeling, we created a rat model induced by a high-fat diet and a model for TMZ treatment. A comparison between the HFD group and control group showed significant weight gain after 12 weeks on a high-fat diet (Fig. 9A). Plasma triglycerides increased significantly, and total cholesterol levels and nonsignificant trends towards elevated LDL levels and decreased HDL levels (Fig. 9B). Following treatment with TMZ, the blood lipid levels of the rats did not show a significant reduction. The control group had a significantly ower MDA level compared to the HFD group, suggesting an increase in oxidation level among the HFD rats. Additionally, the MDA level decreased after TMZ treatment, but this difference was not statistically significant (Fig. 9C). NO content decreased in the high-fat diet group, and increased after TMZ treatment (Fig. 9D). Electrophysiological measurements were conducted on three groups of rats. In comparison to the control group, the AERP decreased significantly in the HFD group, leading to an elevated incidence of AF. Following TMZ therapy, there was an elongation of the AERP and a reduction in the rate of AF induction (Fig. 9E **and F**). We used atrial tissue for Western blotting and observed a decrease in eNOS expression in the HFD group when compared to the control group. Furthermore, the addition of TMZ significantly increased the expression of eNOS. TH, CHAT, and PGP9.5 protein expression was elevated in the HFD group and decreased after TMZ treatment (Fig. 9G). In the high-fat diet group, immunohistochemistry showed a significant increase in the positive density of TH, CHAT, and PGP9.5. However, after TMZ treatment, the positive density decreased significantly (Fig. 9H).

## Discussion

In this investigation, we discovered that PA increased ROS in cardiomyocytes, damaged mitochondria, caused mitochondrial morphology and function changes, and affected NO production through the CRIF1/SIRT1/eNOS axis. Under normal conditions, NO is maintained at a certain level and exerts an inhibitory effect on neuron proliferation by inducing increased nitrosylation of Keap1 to promote transcriptional expression of Nrf2 into the nucleus agonist P21. In response to high-fat stimulation, the NO content decreased. Thus, its inhibitory effect on nerve cells is lost, and remodeling is induced. These effects increase susceptibility to AF. Finally, we found that TMZ supplementation attenuated high-fat-induced neural remodeling and reduced the induction of AF by promoting eNOS expression.

The cardiac autonomic nervous system (ANS) can be divided into extrinsic and intrinsic components [17, 18] and consists of sympathetic and vagus nerves. The sympathetic nerve originates from the interior of the spinal column, while the vagus nerve originates from the medulla. Together, they form ganglia at the fat pad of the pulmonary vein orifice and regulate the balance between the extrinsic and intrinsic nervous systems of the heart [18]. The joint activation of the sympathetic and vagal systems constitutes the substrate for AF episodes [19]. Disturbances in the activity of the intrinsic nerves of the heart have been shown to enhance AF of pulmonary venous origin [20, 21], whereas elimination of the autonomic nerves can suppress AF episodes from the pulmonary veins [22]. The activation of the ANS is associated with many of the risk factors for AF, which may be related to altered atrial electrophysiology with excessive regeneration and inhomogeneity of the distribution of nerves [23]. Intrinsic autonomic modulation can also be the sole trigger for AF [24]. Vagal activity enhances acetylcholine-dependent K currents [25]. β-adrenergic receptor activation promotes Ca2 + inwards flow and facilitates DAD-related ectopic firing by hyperphosphorylation of RyR2 [26]. In dogs with AF simulated by rapid atrial pacing, there is atrial regeneration with hyperinnervation [27]. In our experiments, we confirmed the presence of abnormal nerve proliferation and over-innervation in the atria of rats in the HFD group. Additionally, the AERP was shortened, and the incidence of AF induction increased.

In the present study, we found that high-fat stimulation impaired mitochondrial morphological functions, and the ATP content was decreased in cardiomyocytes. Because of the high energy demand of the heart, mitochondria are abundant in cardiomyocytes and generate significant energy through oxidative phosphorylation [28]. Some studies have confirmed that mitochondrial complexes are damaged in the atrial tissue of patients with AF [29]. A dysfunctional mitochondrial system leads to an overabundance of reactive oxygen species (ROS), which eventually leads to oxidative stress [30]. Dysfunctional mitochondria affect cellular respiration and excess ROS are generated during energy production, causing oxidative stress [31]. Maintaining normal physiological functions requires a specific amount of ROS. However, an excess of ROS can cause oxidative damage to proteins, lipids, and nucleic acids [32]. This exacerbates mitochondrial damage and creates a vicious cycle [33]. In AF, the atria undergo rapid and irregular beating, which increases energy demand. Damage to mitochondrial function can lead to an untimely energy supply to the atrial muscle, further aggravating the injury to cardiac function. In addition, when mitochondrial damage occurs, oxidative phosphorylation does not proceed correctly, which can contribute to cellular metabolism shifting towards glycolysis [34, 35]. The adult heart primarily derives its energy primarily from fatty acid oxidation. When the energy supply mode shifts from fatty acid β-oxidation to glycolysis, increased lactate production leads to acidosis. Excessive acidosis results in an overload of Na + due to the exchange of Na+-H + and subsequently leads to an overload of Ca+. This can be secondary to cardiac electrical remodeling and promote the development and progression of AF [36].

This study confirms that high fatty acid stimulation reduces cardiomyocyte NO production. NO, a diffusible and highly reactive free radical, is produced by NOS with arginine as the substrate under the action of cofactor BH4 [10]. NOS has three isoforms: nNOS (neuronal NOS), iNOS (inducible NOS), and eNOS (endothelial NOS). nNOS and eNOS are constitutively expressed in the myocardium, and iNOS is expressed only during inflammatory or pathological states [37]. Studies have demonstrated that the SIRT1/eNOS pathway has a beneficial effect in conditions like ischemia reperfusion [38, 39], atherosclerotic processes [40], and cardiomyopathy [41]. We verified that high-fat stimulation also affects NO production through the SIRT1/eNOS axis. ROS can regulate SIRT1 expression [42], and we reversed the decrease in protein expression of SIRT1 and eNOS caused by high-fat stimulation after the removal of ROS with NAC. CRIF1 can affect mitochondrial respiratory chain oxidative phosphorylation, thereby regulating ROS production [43]. Our previous experiments revealed that high-fat stimulation could damage the mitochondrial respiratory chain in cardiomyocytes. Therefore, we further verified the relationship between CRIF1 and the SIRT1/eNOS axis. The results suggest that CRIF1 plays a role in controlling the acetylation of eNOS through the regulation of SIRT1 expression in response to PA stimulation, thereby influencing the production of NO.

PC12 is a cell line derived from rat adrenal tissue that can mimic multiple functions of primary neurons and has been widely used as a neuronal model [44]. We established a coculture system using H9C2 cardiomyocytes and PC12 neuronal cells in a high-fat environment to investigate the interaction between cardiomyocytes and neuronal cells under high-fat stimulation. We found that high-fat stimulation increased neuronal cell proliferation. In contrast, overexpression of eNOS in cardiomyocytes inhibited neuronal cell proliferation. Several studies have shown that NO negatively regulates cell proliferation [45–47]. As a result, we speculated that NO produced by cardiomyocytes inhibits neuronal cell proliferation. Therefore, we added NO scavengers to the culture medium after overexpression of eNOS in cardiomyocytes, and the inhibition of neuronal cell proliferation after overexpression of cardiomyocyte eNOS was partially counteracted. This finding further confirms that NO plays a bridging role in the high lipid stimulation of cardiomyocytes to affect neuronal cell remodeling.

NO, a typical gas signaling molecule, activates guanylate cyclase (sGC). sGC produces cGMP and activates cGMP-dependent protein kinase (PKG) [48]. Recently, a growing body of research has verified that nitrogen monoxide can chemically attach to the sulfur-containing compound cysteine, resulting in the formation of S-nitrosothiols, a process referred to as S-nitrosylation [49]. This reversible and ubiquitous post-translational modification of proteins regulates various biological activities [11] and can also be regulated by enzymatic degradation. The regulation of S-nitrosylation is significantly influenced by GSNOR, which is an important factor in multiple cardiovascular disorders and can exert anti-inflammatory effects by controlling endothelial protein transport and by inhibiting the expression of proinflammatory factors [50]. Estrogen can exert cardioprotective effects by nitrosylating mitochondria-associated proteins [51]. In addition to the protective effects, one study confirmed that SNO-Hsp90 could exacerbate cardiac hypertrophy [52]. This suggests that S-nitrosylation is a double-edged sword that can have both cardioprotective impacts and exacerbate myocardial damage. The present study confirmed that NO could inhibit neuronal cell proliferation, but the exact underlying mechanism of this effect remains to be further explored.

P21^WAF1/CIP1^ is a broadly acting cell cycle protein-dependent kinase inhibitor that can be regulated by various transcription factors. Maintaining its stability is crucial for the correct progression of the cell cycle and the determination of cellular outcomes [53]. Earlier investigations have indicated that P21 can impede the cell cycle progression in diverse types of tumor cells [54] and suppress the process of skeletal muscle and bone tissue regeneration [55]. P21 also exerts inhibitory effects on proliferation in mammalian cardiac tissue [56, 57]. Prior research has substantiated that in neuronal cells, the expression of P21 could be elevated through the influence of NO, thereby inhibiting proliferation. We predicted transcription factors based on the promoter of P21. Our discovery reveals that Nrf2 effectively binds to the P21 promoter, thereby exerting precise control over the transcriptional regulation of P21. Nrf2, a transcription factor expressed in nearly all tissues, plays a crucial role in the body’s antioxidative processes [58, 59]. Our previous study revealed that Nrf2 could regulate the proliferation of fibroblasts. Therefore, we hypothesized that NO regulates the expression of P21 through Nrf2. Consequently, we applied SNAP to increase NO in cultured neuronal cells. In a dose-dependent manner, we observed that SNAP enhanced the protein levels of Nrf2 and P21. Additionally, the protein expression of P21 decreased upon Nrf2 inhibition through siRNA.

The expression of Nrf2 is tightly regulated in multiple ways, and Keap1, a major regulator of Nrf2 [60], is a zinc finger protein consisting of 624 amino acids with a high cysteine content, forming a homodimer [61]. At the physiological level, Keap1 engages with Nrf2 within the cytoplasm, promoting the ubiquitination and subsequent degradation of Nrf2, thereby maintaining Nrf2 at a reduced concentration [62]. When stimulated, Keap1 dissociates from Nrf2 to allow its entry into the nucleus to produce an effect [63]. S-nitrosylation is a post-translational protein modification, which is the reaction of endogenous NO with thiols of cysteine to form SNO. It has a crucial role in maintaining cardiac function and regulating oxidative stress homeostasis [64]. Keap1 is rich in cysteine, providing a potential target for regulating thiol-responsive chemicals [65]. We verified in vitro that NO could regulate neuronal cell proliferation by increasing Keap1 nitrosylation and thus Nrf2 entry into the nucleus to increase agonistic P21 transcription.

TMZ is a cardioprotective drug that improves myocardial function by modifying myocardial energy metabolism [66]. It has been observed that TMZ exhibits a protective effect in patients with angina pectoris, post-stent placement, and heart failure [67]. Due to its minimal side effects, it has been widely applied in clinical practice [68]. TMZ reduces oxidative stress and improves myocardial ultrastructural remodeling in dogs with AF by activating eNOS. This reduces the rate of AF induction and duration of AF [69]. It has been reported that TMZ may exert antioxidant effects and improve cardiac function by increasing the expression of eNOS [70, 71]. In our study, we constructed a high-fat environmental coculture model and the HFD rat model. We verified that TMZ could partially reverse high-fat-induced atrial neural remodeling by restoring myocardial eNOS expression and NO levels. TMZ supplementation may be considered part of the treatment regimen for high-risk patients with elevated lipid levels. However, it is essential to emphasize that further research is necessary to fully understand its effectiveness. Our study provides a foundation for the potential investigation of TMZ as an adjunct therapy for cardiac protection in high-lipid patients, acknowledging that further extensive research is needed to validate its efficacy.

### Strengths and limitation

This study effectively validates the significance of the CRIF1/eNOS/P21 pathway in atrial neural remodeling triggered by high-fat stimulation. Additionally, it firmly establishes the impact of TMZ on the modulation of atrial neural remodeling resulting from high-fat stimulation. These findings provide valuable new perspectives that could potentially contribute to the advancing clinical approaches for managing atrial fibrillation.

## Conclusions

In this study, it became evident that high-fat stimulation damaged cardiomyocyte mitochondria, affected mitochondrial morphology and function, and decreased NO production through the CRIF1/SIRT1/eNOS axis, thereby allowing neuronal cell proliferation and leading to remodeling. Increasing NO levels increased Keap1 nitrosylation, promoting Nrf2 entry into the nucleus to activate P21 expression. Therefore, neuronal cell proliferation is inhibited in the high-fat stimulated myocardial microenvironment. TMZ inhibits neural remodeling caused by high-fat stimulation by increasing the protein expression of eNOS and decreasing the rate of AF induction. Therefore, we believe that considering the addition of TMZ for myocardial protection may be appropriate when treating high-risk patients with elevated lipid levels.

### Electronic supplementary material

Below is the link to the electronic supplementary material.

Supplementary Material 1

Supplementary Material 2

Supplementary Material 3

Supplementary Material 4

## Data Availability

The data presented in this study are available in the article and Supplementary Materials.
