# Supplementary figures and images for: High-fat stimulation induces atrial neural remodeling by reducing NO production via the CRIF1/eNOS/P21 axi
Source: Lipids Health Dis. 2023 Nov 6;22:189. doi: 10.1186/s12944-023-01952-7 (PMC10629039; doi:10.1186/s12944-023-01952-7)

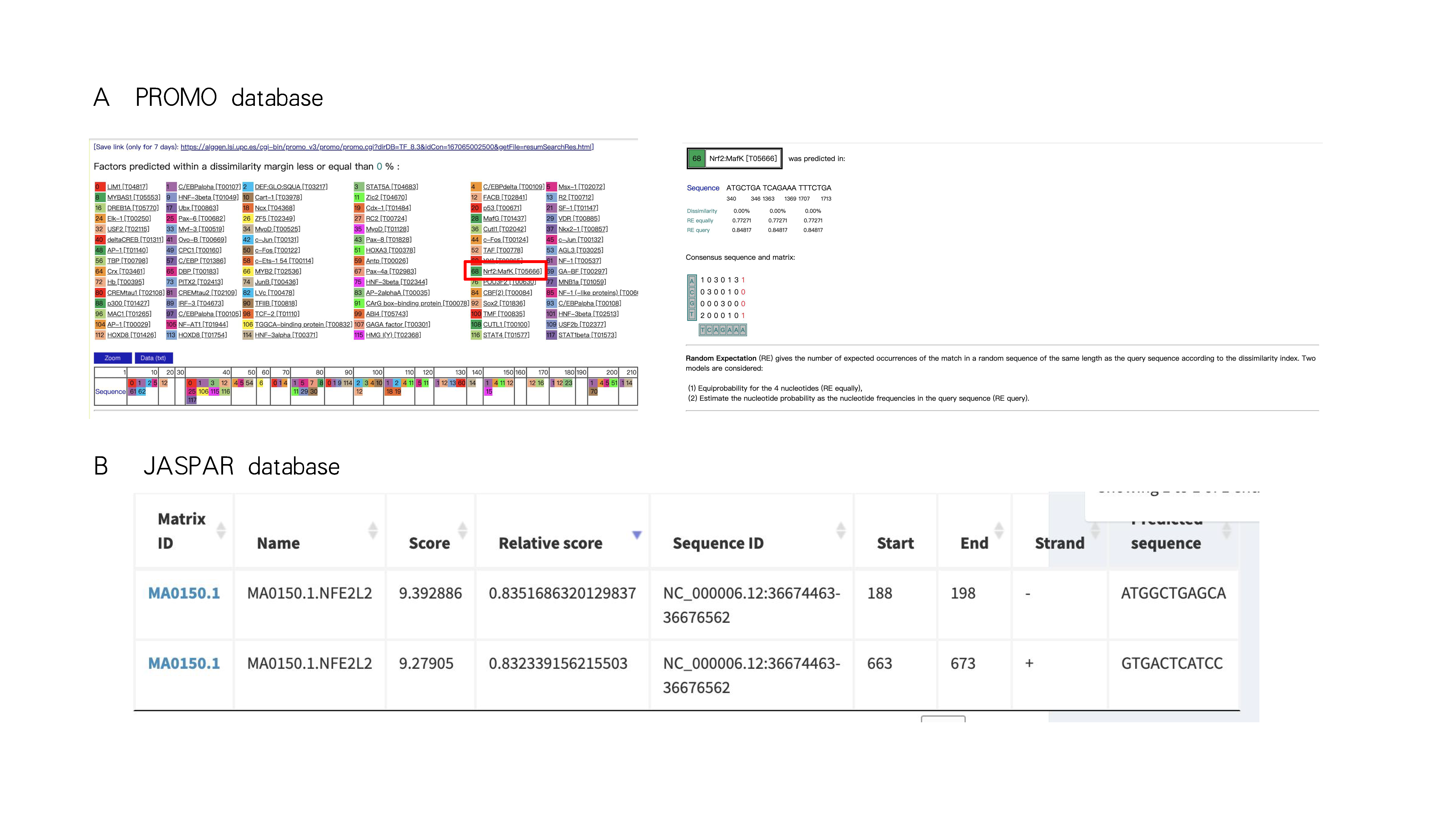

Supplement: Supplementary file 1 — Supplementary Material 1 [file 12944_2023_1952_MOESM1_ESM.png]
