# Supplementary material for: High-fat stimulation induces atrial neural remodeling by reducing NO production via the CRIF1/eNOS/P21 axi
Source: Lipids Health Dis. 2023 Nov 6;22:189. doi: 10.1186/s12944-023-01952-7 (PMC10629039; doi:10.1186/s12944-023-01952-7)
Supplement: Supplementary file 2 — Supplementary Material 2 [file 12944_2023_1952_MOESM2_ESM.pdf]

202309101314388982370975250l4528

WORD COUNT 9164

TIME SUBMITTED 10-SEP-2023 01:15PM

PAPER ID 102616805

Article

High-fat stimulation induces atrial neural remodelling by reducing NO production via the CRIF1/eNOS/P21 axis

Abstract

Background

Atrial autonomic restructuring is a crucial underlying mechanism of atrial fibrillation (AF) has a significant impact on the progression of AF. Hyperlipidemia is a predisposing factor for AF, but its effect on atrial nerve remodelling is unclear. The objective of this study was to explore the possible mechanisms through which consumption of a high-fat diet (HFD) induces remodeling of atrial nerves, and to identify novel targets for clinical intervention.

Methods

Cell models were created in vitro by subjecting cells to palmitic acid (PA), whereaswhile rat models were establishedformed by feeding a high-fat diet. To examine the interaction between cardiomyocytes and nerve cells in a coculture system, Transwell cell culture plates with a pore size of 0.4 μm were employed. The CCK-8 assay was employed to determine cell viability, fluorescent probe DCFH-DA and flow cytometry were utilized for measuring ROS levels, JC-1 was used to assess the mitochondrial membrane potential, the Griess method was employed to measure the nitric oxide (NO) level in the supernatant, a fluorescence-based method was used to measure ATP levels, and MitoTracker was utilized for assessing mitochondrial morphology. The expression of pertinent proteins was evaluated using wWestern blotting (WB) and immunohistochemistry techniques. SNAP was used to treat nerve cells in order to replicate a high-NO atmosphere, and the level of nitroso was assessed using the iodoTMT reagent labeling method.

Results

The study found that cardiomyocytes' mitochondrial morphology and function were impaired under high-fat stimulation, affecting nitric oxide (NO) production through the CRIF1/SIRT1/eNOS axis. In a coculture model, overexpression of eNOS in cardiomyocytes increased NO expression. Moreover, the increased Keap1 nitrosylation within neuronal cells facilitated the entry of Nrf2 into the nucleus, resulting in an augmentation of P21 transcription and a suppression of proliferation. Atrial neural remodelling occurred in the HFD rat model and was ameliorated by increasing myocardial tissue eNOS protein expression with trimetazidine (TMZ).

Conclusions

Neural remodeling is triggered by high-fat stimulation, which decreases the production of NO through the CRIF1/eNOS/P21 axis. Additionally, TMZ prevents neural remodeling and reduces the occurrence of AF by enhancing eNOS expression.

Keywords: Atrial fibrillation; High-fat; Nitric oxide; Nitrosylation; Neural remodelling

Introduction

Atrial fibrillation (AF) is the prevailing clinical irregular heartbeat [1], and in recent times, its frequency has been progressively rising annually[2]. AF is marked by a high occurrence of other medical conditions and an elevated likelihood of mortality, yet the factors causing it are not thoroughly comprehended. The main pathogenesis of AF includes electrical, structural, neural, and metabolic remodelling[3]. Certain research has indicated that neural restructuring plays a crucial part in the growth and sustenance of AF. Neurological remodelling includes the overproliferation of nerves, heterogeneity of distribution, and enhanced activity. Hyperlipidemia is an elevation of lipids in the blood due to abnormal metabolic function[4]. Hyperlipidemia can affect cardiac function in several ways, and it is well known to cause atherosclerosis. Research has indicated that hyperlipidemia has the potential to cause oxidative stress and trigger proinflammatory reactions, resulting in the disturbance of myocardial homeostasis[5]. Myocardial remodeling and heightened vulnerability to atrial fibrillation occur due to mitochondrial damage, mast cell activation, and subsequent degranulation[6, 7]. In excess, reactive oxygen species (ROS) can harm mitochondria [8, 9], and disturb the organism's redox balance. There is evidence that excessive lipids can cause oxidative stress that increases the risk of AF [10]. Nitric oxide (NO) can also be directly reduced when ROS levels are elevated [11]. NO is a gas that acts as a signaling molecule and is produced by nitric oxide synthase (NOS)[12]. It is involved in regulating cell growth, metabolism, and exerting anti-inflammatory effects. Regulating cardiovascular homeostasis is a crucial element. Furthermore, NO groups have the ability to covalently alter cysteine thiols, resulting in the production of S-nitrosylated thiols (SNO)[13], which is referred to as S-nitrosylation, in addition to activating the conventional cyclic guanosine monophosphate (cGMP)-dependent signaling pathway. In persistent AF animal models, reduced eNOS expression and decreased circulating NO levels have been demonstrated[14, 15]. Under normal physiological conditions, NO inhibits the proliferation of neuronal cells[16]. Some studies have confirmed that increasing atrial muscle NO content in dogs with AF can inhibit autonomic remodelling, but the exact mechanism is unknown[17].

In our prior research, it was discovered that the stimulation of a diet rich in fats could enhance the development of atrial fibrillation caused by myocardial fibrosis through the initiation of programmed cell death in the heart muscle cells. In order to explore the potential mechanisms through which high-fat conditions contribute to the progression of AF, the present study hypothesizes that high-fat stimulation induce atrial fibrillation (AF) by disrupting the balance of nitric oxide (NO) and promoting atrial nerve remodeling through the CRIF1/eNOS/P21 axis via mitochondrial damage.

Materials and Methods

Cell culture

Fu Heng Biotechnology (Shanghai, China) provided rat cardiomyocytes (H9C2) and rat adrenal pheochromocytoma cells (PC12). Cell lines were cultured in DMEM (Invitrogen, Waltham, USA) supplemented with 10% FBS at 37°C under 5% CO_2.Sigma (St. Louis, MO) was where the purchase of Palmitic acid (PA) took place. Unless otherwise noted, all drugs used in this experiment were purchased from MCE

Cell viability assay

Once the cell model was built, we added 10μL of Cell Counting Kit-8 (CCK-8) solution to every well. This was followed by a 2-hour incubation period in a cell incubator. Subsequently, we assessed the absorbance at 450 nm.

Western blotting

The lysate mixture was made by combining RIPA lysis buffer and PMSF (Beyotime, Shanghai, China). Next, the lysate mixture was introduced to the samples, subjected to lysis on ice, and then centrifuged at a speed of 12000 revolutions per minute for a duration of 5 minutes at a temperature of four degrees. The liquid above the sediment was combined with 5× SDS–PAGE sample loading buffer and incubated for 10 minutes at 95°C in a metal bath before being frozen at -20°C.Proteins were isolated by employing SDS–PAGE (EpiZyme, Shanghai, China) on a 15*15 gel containing 15 μg protein in each lane. The proteins were transferred onto PVDF membranes, followed by a 1-hour blocking step using skimmed milk powder. Afterwards, the primary antibody was incubated with them overnight at a temperature of 4°C.The membrane was subjected to three successive 5-minute rinses with TBST, then incubated for 1 hour with horseradish enzyme-conjugated goat anti-mouse/rabbit IgG (H+L) (ZSGB-BIO, China). Subsequently, it underwent an additional series of three 5-minute TBST rinses. The membrane was coated with a uniform layer of ECL luminescent solution obtained from Millipore in Germany, and then it was exposed.

Table1. List of all antibodies used in the study:

Name	Article number	Dilution ratio	Company
β-actin	3700	1:1000	CST
CHAT	ab13786	1:1000	Abcam
eNOS	ab199956	1:1000	Abcam
Keap1	10503-2-AP	1:2000	Proteintech
Mfn1	ab221661	1:1000	Abcam
Mfn2	12186-1-AP	1:2000	Proteintech
MTCO1	ab203912	1:1000	Abcam
NDUFB8	ab192878	1:1000	Abcam
Nrf2	ab109199	1:1000	Abcam
PGP9.5	ab108986	1:1000	Abcam
SDHA	ab137040	1:1000	Abcam
SIRT1	9475	1:1000	CST
TH	sc25269	1:1000	Santa cruz
UQCRC2	ab203832	1:1000	Abcam

ROS and fluorescence measurement

The ROS fluorescent probe (DCFH-DA) was diluted 1:2000 in DMEM. Then, the medium was aspirated and discarded. The probe was introduced and left to incubate for a duration of 30 minutes at a temperature of 37 degrees Celsius. The probe was then discarded, and DMEM was used three times to wash the cells. Next, the fluorescence intensity was measured using a Leica fluorescence microscope, with an excitation wavelength of 488 mm. Establish a suitable threshold for fluorescence intensity and compare the ratio of cells

exhibiting a positive signal at the designated threshold to the overall cell count.

JC-1 assay

The JC-1 assay was performed using the JC-1 kit (Beyotime, Shanghai, China) The JC-1 working solution was diluted 1:200 with JC-1 buffer. Following incubation, the liquid above the sediment was removed, and the cells were washed three times using JC-1 buffer. Next, a sufficient amount of medium was introduced, and the cells were examined using a fluorescence microscope (Leica, Wetzlar, Germany). It was detected using a 490 nm excitation wavelength and a 530 nm emission wavelength for JC-1 monomers, and a 525 nm excitation wavelength and a 590 nm emission wavelength for JC-1 aggregates.

ATP detection

The cells were lysed on ice using the improved ATP assay kit (Beyotime, Shanghai, China), then centrifuged at 12,000×g for 5 minutes at 4°C.Afterwards, the liquid above was combined with the ATP assay solution, and the resulting intensity of chemiluminescence (measured in RLU) was documented using a chemiluminescence device.

NO content measurement

The cells were lysed on ice using the improved ATP assay kit (Beyotime, Shanghai, China), then centrifuged at 12,000 × g for 5 minutes at 4°C.Afterwards, the liquid above was combined with the ATP assay solution, and the resulting intensity of chemiluminescence (measured in RLU) was documented using a chemiluminescence device.

Cell transfection

Transfection was performed when the cell confluence was 60-70%. Opti-MEM (Gibco, NY, USA) was used to replace the culture medium. Lipo2000 (Invitrogen, Waltham, USA) was mixed with plasmid or siRNA (GenePharma, Shanghai, China). The blend was slowly introduced into the cells and subsequently replaced with complete medium for a duration of 4-6 hours.

Expression of eNOS acetylation

Using the immunoprecipitation technique, we observed the presence of eNOS acetylation. The precipitation of proteins was achieved by utilizing an anti-eNOS antibody (Santa Cruz, Cat#sc-376751) that was cross-linked to Protein A/G magnetic beads (MCE, HY-K0202). Detection of the proteins was carried out through Western blotting using an anti-acetyl-lysine antibody (CST, Cat#9441).

Immunofluorescence

After removing the medium, the cells were rinsed three times with PBS. Afterwards, we introduced 4% paraformaldehyde into the mixture and allowed it to react for a duration of 15 minutes. Afterward, the cells underwent another PBS wash, and permeabilization buffer was applied for 10 minutes. After another wash, the cells were incubated with blocking buffer for 10 minutes.

After incubating overnight at 4°C, the primary antibody was removed by washing the next day. Next, the luminescent secondary antibody was introduced and left to incubate for 1.5 hours in a lightless environment. This was followed by DAPI (Beyotime, Shanghai, China) staining. Subsequently, an anti-fluorescence quenching reagent was employed to encapsulate the film, followed by examination of the outcomes using a confocal microscope (Nikon, Tokyo, Japan). The antibodies utilized included Nrf2 (Proteintech, Cat#16396-1-AP).

Nitrosylation assay

Nitrosylation was detected using the S-nitrosylation assay kit (Thermo, 90105). The protein was enriched using a Keap1 antibody (Proteintech, Cat#10503-2-AP) cross-linked with magnetic beads. Next, the protein was extracted, and the concentration of the protein sample was modified to 1 mg/ml by utilizing lysate. Next, half of the total was combined with 5 times the loading buffer and heated at a temperature of 95°C for a duration of 10 minutes. The other 1/2 was added to a corresponding volume of MMTS and shaken vigorously to block free sulfhydryl acetone to precipitate the protein and remove excess MMTS. This was then resuspended using HENS buffer, and sodium ascorbate was added to reduce S-nitrosocysteine. Then, it was labelled using iodoTMT reagent, and an anti-TMT antibody was used for Western blot detection.

EdU

We performed EdU detection using the kit purchased from Beyotime(Cat# C0075S). After completing the stimulation, an equal volume of EdU reaction solution was added to the culture medium, and the cells were incubated at 37°C for 2 hours. The reaction solution was prepared as per the manufacturer's instructions and added after washing and fixation, followed by a light-protected incubation for 20 minutes. Cell nuclei were labeled with Hoechst staining after washing with PBS. Fluorescence microscopy was utilized for detection, with a maximum excitation wavelength of 346nm and a maximum emission wavelength of 565nm.

Animal grouping and modelling

Male Sprague–Dawley (SD) rats, aged eight weeks and weighing approximately 200 g (±20 g), were acquired from Viton Lever Laboratory Animal Technology Co. The rodents were kept in a chamber where the temperature was controlled at (20±2) °C and exposed to a 12-hour period of light followed by a 12-hour period of darkness. A total of thirty rats were divided into three groups: control (n=10), HFD(n=10), and HFD+TMZ (n=10). Throughout the 12-week study duration, the control group was provided with a standard diet. Following an eight-week period of high-fat diet (HFD) consumption, TMZ (MCE, HYB0968A) was given via intraperitoneal injection at a daily dose of 7 mg/kg for a duration of four weeks. Thirty percent of the calories from fat were derived from palm oil.

Programmed electrical stimulation

Through the right jugular vein, electrodes were inserted into the high right atrium. S1S2 electrical stimulation was administered using a multichannel electrical stimulator with a frequency ratio of 8:1, an S1S1 interval of 120ms, and an S1S2 initial interval of 60ms, decreasing by 2ms each time. The longest interval between S1 and S2 that could not induce atrial stimulation was known as the atrial effective refractory period (AERP). A fast irregular atrial rate lasting at least 2s was considered successfully inducing AF.

Measurement of biochemical blood indexes

Blood was collected from the apical part of the heart after the animals were anaesthetized. After being spun at a speed of 2000 revolutions per minute for a duration of 20 minutes at ambient temperature, the serum was subsequently preserved at a temperature of -

80°C.Cholesterol levels, including HDL, LDL, and total cholesterol, were assessed with a fully automated biochemical analyzer from Roche in Switzerland. Additionally, triglycerides and malondialdehyde (MDA) levels were measured using a commercial kit provided by Elabscience in Wuhan, China.

Immunohistochemistry

Following the collection of tissue, the samples were immersed in 4% paraformaldehyde for a duration of 48 hours. Paraffin wax was used to embed them, which was then cut into sections of 5μm. These sections underwent a gradient dewaxing process and antigen retrieval using citric acid solution. The activity of endogenous peroxidase was inhibited by applying 3% hydrogen peroxide for a duration of 15 minutes. Following rinsing, the sections were obstructed using a 3% BSA solution for a duration of 30 minutes. Subsequently, they were incubated with the primary antibody at a temperature of 4°C overnight. On the next day, paraffin sections were brought to room temperature for 30 minutes and rinsed with PBS three times, with each rinse lasting 5 minutes. Afterwards, the second antibody was added gradually and left to incubate at ambient temperature for a duration of 50 minutes. After washing, DAB was added dropwise, and the reaction was terminated by microscopic control of colour development time and rinsing after the appearance of positivity. Haematoxylin staining was rinsed after 1 min, and the haematoxylin differentiation solution was applied for several seconds. After dehydration and sealing, microscopic observation was performed, and data were collected. The following antibodies were used: PGP9.5(1:200, Cat# ab108986, Abcam), TH (1:200, Cat# sc25269, Santa cruz), CHAT (1:200, Cat# ab137869, Abcam).

Statistical methods

Each experiment was conducted with triplicate independent samples. Statistical analysis was performed using unpaired t-tests for comparisons between two groups, and one-way ANOVA was employed for pairwise comparisons among three or more groups. GraphPad Pro 9.0, located in San Diego, CA, was utilized for the statistical analyses. The mean ± SD values were used to present the results, and statistical significance was determined at a threshold of $P < 0.05$. In all figures, the symbols indicate statistical significance levels as follows: * = p<0.05, ** = p<0.01, *** = p<0.001, and **** = p<0.0001.

Results

PA increases ROS in cardiomyocytes.

The CCK-8 method was used to evaluate cell viability after stimulating H9C2 cells with various concentrations of PA (ranging from 0 to 200μM). There was no notable disparity in cell viability when exposed to PA concentrations ranging from 0 to 150μM, but a decline in cell viability was observed at a PA concentration of 200μM.For the time-dependent cell viability assay, we chose 150μM. There was no notable variation in cell viability within 36 hours, but a decline in cell viability was observed after 48 hours (Fig.1A). Therefore, we used a PA concentration of 150μM and a stimulation time of 36 h for subsequent experiments. We used the DCFH-DA fluorescent probe to detect intracellular ROS production after PA stimulation and found that PA stimulation increased intracellular ROS production by flow cytometry and cytofluorimetry.

Fig. 1 PA increases ROS levels in cardiomyocytes. The CCK-8 assay was conducted following stimulation with PA at concentrations of 0, 10, 50, 100, 150, or 200 μM, or after 150 μM PA stimulation for durations of 0, 6, 12, 24, 36, or 48 h. Flow cytometry was employed to measure ROS levels following PA stimulation. ROS production following PA stimulation was detected using fluorescence microscopy. Scale bar=50μm. *= p<0.05, ** = p<0.01, *** = p<0.001, and **** = p<0.0001.

PA impaired mitochondrial morphology and function

ROS are mainly produced by mitochondria. Thus, we applied MitoTracker to label mitochondria, which are elongated and thread-like under normal conditions. After stimulation with 150μM PA for 36 h, mitochondrial fracture increased, and fragmentation became more apparent (**Fig. 2B**). Western blotting showed that mitofusin1(Mfn1) and mitofusin 2(Mfn2) protein expression decreased (**Fig. 2A**). These findings indicate that PA stimulation could cause changes in mitochondrial fusion morphology and increase the proportion of damaged mitochondria. To further verify the function of mitochondria, Western blotting was used to detect decreased mitochondrial complex I, II, III, and IV protein expression (**Fig. 2C**). JC-1 staining showed reduced mitochondrial membrane potential (**Fig. 2D**) and decreased ATP levels (**Fig. 2E**). These results indicate that mitochondrial function was impaired.

Fig. 2 PA impairs mitochondrial morphology and function. (A) WB detection of Mfn1 and Mfn2 protein expression after PA stimulation. After PA stimulation, MitoTracker Red staining revealed the morphology of the mitochondria. The scale bar measures 100 micrometers. (C) WB detection of the protein expression of mitochondrial respiratory chain complexes after PA stimulation. JC-1 detected the mitochondrial membrane potential following stimulation with PA. Scale bar=50μm.(E) Myocardial ATP content after PA stimulation. *= p<0.05, ** = p<0.01, *** = p<0.001, and **** = p<0.0001.

PA-stimulated generation of ROS regulates SIRT1/eNOS expression.

Under stimulation with 150 μM PA for 36 h, the NO content in the cell supernatant decreased (**Fig. 3A**), and a decrease was observed in the expression of sirtuin1(SIRT1) and endothelial nitric oxide synthase(eNOS) proteins (**Fig. 3B**). After using NAC to scavenge ROS, the PA-induced downregulation of SIRT1 and eNOS was partially reversed (**Fig. 3C**). In order to confirm the correlation between SIRT1 and eNOS, we introduced the SIRT1 overexpression plasmid and siRNA into H9C2 cardiomyocytes. As a result of silencing SIRT1, the expression of eNOS decreased, whereas SIRT1 overexpression led to an increase in eNOS expression (**Fig. 3D and E**). SIRT1, a protein deacetylase dependent on nuclear NAD+, controls protein expression by deacetylating them. Therefore, we also examined the acetylation level of eNOS. Overexpressing SIRT1 decreased eNOS acetylation, whereas silencing SIRT1 resulted in an increase in eNOS acetylation (**Fig. 3F**).

Fig. 3 PA-stimulated generation of ROS regulates SIRT1/eNOS expression. (A) NO content in the supernatant after PA stimulation. Detection of SIRT1 and eNOS protein levels after PA stimulation using WB. Detection of SIRT1 and eNOS protein levels was performed using WB after scavenging ROS with NAC (5 mM). (D) After overexpressing SIRT1, the levels of SIRT1 and eNOS proteins were detected, along with the measurement of NO levels in the supernatant. After silencing SIRT1, an analysis was conducted on the levels of SIRT1 and eNOS proteins, as well as the levels of NO in the supernatant.(F) Levels of eNOS acetylation after overexpression of SIRT1 or silencing of SIRT1.

PA regulates SIRT1/eNOS expression through CRIF1 and affects NO production.

CR6-interacting factor 1 (CRIF1), an essential mitochondrial protein, plays a key role in assembling mitochondrial oxidative phosphorylation complexes. In response to PA stimulation, CRIF1 protein expression was reduced (**Fig. 4A**). Following the utilization of siRNA to silence CRIF1, the supernatant exhibited a decline in NO levels, along with a decrease in the protein expression of SIRT1 and eNOS (**Fig. 4B and C**). As a result, our hypothesis was that PA stimulation caused harm to the mitochondria. This decreased CRIF1 expression, reducing cellular NO production through the SIRT1/eNOS axis. In order to confirm the connection between CRIF1, SIRT1, and eNOS, we proceeded to enhance the expression of SIRT1 following the suppression of CRIF1.In our findings, it was discovered that the increased expression of SIRT1 partially counteracted the reduction in eNOS protein levels resulting from the suppression of CRIF1 (**Fig. 4D**) and decreased the acetylation level of eNOS (**Fig. 4E**).

Fig. 4 PA regulates SIRT1/eNOS expression through CRIF1 and affects NO production. (A) WB detection of CRIF1 protein expression after PA stimulation.(B) NO content in the supernatant after silencing CRIF1.(C) WB detection of CRIF1, SIRT1, and eNOS protein expression after silencing CRIF1.(D) WB detection of CRIF1, SIRT1, and eNOS protein expression after silencing CRIF1 and overexpressing SIRT1 again.(E) eNOS acetylation levels after silencing CRIF1 again overexpressing SIRT1.

PA induces PC12 cell proliferation in a coculture model of H9C2 cardiomyocytes and PC12 neuronal cells.

In order to assess the impact of cardiomyocytes on PA-stimulated neuronal cells, a coculture model consisting of H9C2 cells and PC12 cells was established by culturing them together in a Transwell system (**Fig. 5A**). Following the addition of PA, there was a reduction in the NO content found in the supernatant (**Fig. 5B**), while the protein expression of TH and PGP9.5 increased in PC-12 cells. Furthermore, the expression of P21 protein was observed to rise (**Fig. 5C**), and the EdU assay indicated an augmentation in the percentage of PC12 cells undergoing proliferation (**Fig. 5D**).

Fig. 5 PA induces PC12 cell proliferation in a coculture model of H9C2 cardiomyocytes and PC12 neuronal cells. (A) Schematic diagram of the coculture model. (B) NO content in the supernatant of the coculture model after PA stimulation. (C) WB detection of P21, PGP9.5, and TH protein expression in lower PC12 neuronal cells in the coculture model after PA stimulation. (D) EdU assay of proliferation levels of lower layer PC12 neuronal cells in the coculture model after PA stimulation. Scale bar=100μm.

Overexpression of eNOS in cardiomyocytes increased the NO content and inhibited the proliferation of lower neuronal cells.

After the upregulation of eNOS in the upper layer of H9C2 cardiomyocytes, Supernatant nitric oxide (NO) concentration increased (**Fig. 6A**). Furthermore, the protein levels of P21 in the neuronal cells located in the lower compartment of the coculture setup exhibited an increase, while the expression of TH and PGP9.5 showed a decline (**Fig. 6B**). EdU showed a decrease in the proportion of proliferating cells (**Fig. 6C**). n order to confirm if NO has a suppressive impact on neural remodeling, we introduced a nitric oxide scavenger (carboxy-PTIO) to the upper layer's supernatant subsequent to the overexpression of eNOS, resulting in a decrease in the NO level (**Fig. 6D**). Furthermore, in the lower chamber's

neuronal cells, there was a decrease in the expression of P21, while the expression of tyrosine hydroxylase (TH) and protein gene product 9.5 (PGP9.5) showed an increase (**Fig. 6E**), and EdU showed an increase in the proportion of proliferating cells (**Fig. 6F**).

Fig. 6 Overexpression of eNOS in cardiomyocytes increased NO content and inhibited the proliferation of lower neuronal cells. (A) NO content in the supernatant of the coculture model after PA stimulation in the upper cardiomyocytes overexpressing eNOS. (B) WB detection of P21, PGP9.5, and TH protein expression in lower layer PC-12 neurons after PA stimulation in upper layer cardiomyocytes overexpressing eNOS. (C) EdU assay of proliferation levels of lower layer PC-12 neuronal cells after PA stimulation in an upper layer cardiomyocyte coculture model overexpressing eNOS. Scale bar=100μm. (D) NO content in the supernatant of the coculture model after PA stimulation in the upper layer of cardiomyocytes overexpressing eNOS following the the addition of C-PTIO to remove NO. (E, F) Following the addition of PA to the coculture system, eNOS was observed to be overexpressed in the upper chamber of H9C2 cells. Afterwards, CPTIO was introduced to eliminate NO, and WB analysis was conducted to measure the protein expression levels of P21, PGP9.5, and TH in PC12 cells located in the lower chamber. PC12 cell proliferation was evaluated using the EdU assay. Scale bar=100μm.

NO inhibits proliferation by nitrosylating Keap1 to promote Nrf2 entry into the nucleus to agonize P21 transcription.

We preliminarily demonstrated that NO could promote the protein expression of P21 to inhibit neuronal cell proliferation, but the exact underlying mechanism of this effect remains unknown. Hence, in order to clarify the molecular mechanism through which NO enhances the expression of P21 protein, we employed the PROMO database (http://alggen.lsi.upc.es/cgi-bin/promo_v3/promo/promoinit.cgi?dirDB=TF_8.3)and the JASPER database(https://jaspar.genereg.net/) for the prediction of potential transcription factors (TFs) that bind to the P21 promoter. The P21 promoter region was predicted to be bound by Nuclear factor E2-related factor 2 (Nrf2) with strong affinity, resulting in positive regulation of P21 **(Supplementary Figure1 A and B)**. We hypothesized that NO regulates the expression of P21 by agonizing Nrf2. We treated PC12 neuronal cells with SNAP (an NO donor) to test this conjecture. SNAP increased the NO concentration in the supernatant (**Fig. 7A**) in a concentration-dependent manner to activate the expression of Nrf2 and P21 (**Fig. 7B**). Nrf2 entry into the nucleus was increased after SNAP stimulation (**Fig. 7C**). Following the addition of SNAP, we suppressed Nrf2 expression by utilizing si-Nrf2, resulting in a decrease in the protein expression of P21 (**Fig. 7D**). Nrf2 expression is tightly controlled through various mechanisms, with Keap1 serving as the primary regulator.After the introduction of SNAP (500 μM) to PC-12 neuronal cells, we observed a notable rise in the nitrosylation of the Kelch-like ECH-associated protein 1 (Keap1) protein (**Fig. 7E**). THence, it can be inferred that NO enhances the nitrosylation of Keap1, preventing Keap1 from degrading Nrf2. As a result, Nrf2 gains access to the nucleus and stimulates the transcription of P21, leading to an antiproliferative impact.In a coculture model, we proceeded to confirm the functions of NO and Nrf2.Following the overexpression of eNOS in the upper stratum of H9C2 cells, si-Nrf2 was administered in the lower stratum of PC-12 cells to suppress the Nrf2 expression.In PC12 cells, the findings indicated a reduction in P21 expression and a rise in TH and PGP9.5 expression (**Fig. 7F**). EdU showed an increase in the proportion of proliferating neuronal cells (**Fig. 7G**).

Fig. 7 NO inhibits proliferation by nitrosylating Keap1 to promote Nrf2 entry into the nucleus to induce P21 transcription. (A, B) NO content and protein expression of Nrf2 and P21 in the supernatant after stimulation of PC12 neuronal cells using 0, 10, 50, 100, 200, and 500μM SNAP. (C) Immunofluorescence detection of Nrf2 protein expression after the addition of 500μM SANP. Scale bar=20μm. (D) Protein expression of Nrf2 and P21 after silencing the expression of Nrf2 after adding 500μM SNAP. (E) Detection of nitrosylation of Keap1 after adding 500μM SNAP. (F, G) Upon PA stimulation, after overexpression of eNOS in upper cardiomyocytes and silencing of Nrf2 in lower neuronal cells, WB detected P21, PGP9.5, and TH protein expression, and EdU showed the proportion of neuronal cell proliferation. Scale bar=100μm.

Trimetazidine inhibits neural remodelling by promoting the protein expression of eNOS.

We added TMZ to H9C2 cardiomyocytes. TMZ increased the supernatant NO content (**Fig. 8A**) and upregulated the protein expression of eNOS (**Fig. 8B**) in a concentration-dependent manner. In order to confirm the role of TMZ in neural remodeling induced by high-fat, we added TMZ to a high-fat conditioned coculture model. We found that TMZ increased the NO content in the supernatant (**Fig. 8C**), increased the expression of P21, and decreased the expression of TH and PGP9.5 in PC12 cells (**Fig. 8D**). Edu showed a decrease in the proportion of proliferating cells (**Fig. 8E**).

Fig 8. Trimetazidine inhibits neural remodelling by promoting eNOS protein expression. (A, B) NO content in the supernatant and protein expression of eNOS after stimulation of cardiomyocytes using 0, 10, 50, and 100μM TMZ. (C) NO content in the supernatant after PA stimulation in a culture model with the addition of TMZ. (D, E) WB detected the protein expression of P21, PGP9.5, and TH in lower-layer PC12 neuronal cells after PA stimulation in a culture model with the addition of TMZ; EdU assay of the proliferation level of lower layer PC12 neuronal cells. Scale bar=100μm. Each experiment was repeated at least three times. *= $p<0.05$, ** = $p<0.01$, *** = $p<0.001$, and **** = $p<0.0001$.

HFD induced atrial neural remodelling and increased the induction rate of AF, and TMZ improved neural remodelling and decreased the induction rate of AF.

In order to validate the neuroprotective effect of TMZ against high-fat-induced neural remodeling, we created a rat model induced by a high-fat diet and a model for TMZ treatment. A comparison between the HFD group and control group showed significant weight gain after 12 weeks on a high-fat diet (**Fig. 9A**). Plasma triglycerides increased significantly and total cholesterol levels and nonsignificant trends towards elevated LDL levels and decreased HDL levels (**Fig. 9B**). After treatment with TMZ, the blood lipid levels of the rats were not significantly reduced. The control group had a significantly lower MDA level compared to the HFD group, suggesting an increase in oxidation level among the HFD rats. Additionally, the MDA level decreased after TMZ treatment, but this difference was not significant (**Fig. 9C**). NO content decreased in the high-fat diet group, and increased after TMZ treatment (**Fig. 9D**). Electrophysiological measurements were conducted on three groups of rats. In comparison to the control group, the AERP decreased significantly in the HFD group, leading to an elevated incidence of AF. Following TMZ therapy, there was an elongation of the AERP and a reduction in the rate of AF induction (**Fig. 9E and F**). We used atrial tissue for Western blotting and observed a decrease in eNOS expression in the HFD group when compared to the control group. Furthermore, the

addition of TMZ significantly increased the expression of eNOS. TH, CHAT, and PGP9.5 protein expression was elevated in the HFD group and decreased after TMZ treatment **(Fig. 9G)**. In the high-fat diet group, immunohistochemistry showed a significant increase in positive density of TH, CHAT, and PGP9.5. However, after TMZ treatment, the positive density decreased significantly (**Fig. 9H**).

Fig. 9 HFD induced atrial neural remodelling and increased the induction rate of AF, and TMZ improved neural remodelling and decreased the induction rate of AF. (A) Body weight trends of rats in the CON, HFD, and HFD+TMZ groups. (B) Blood biochemical indexes of rats in the CON, HFD, and HFD+TMZ groups. (C) MDA levels of rats in the CON, HFD, HFD+TMZ groups. (D) NO levels of rats in the CON, HFD, HFD+TMZ groups. (E, F) Cardiac electrophysiological indexes of the hearts of rats in the CON, HFD, and HFD+TMZ groups. (G) WB was used to detect the protein expression of PGP9.5, TH, CHAT, and eNOS in rats in the CON, HFD, and HFD+TMZ groups. (H) Immunohistochemistry of PGP9.5, TH, and CHAT in rats of the CON, HFD, and HFD+TMZ groups. Scale bar=100μm.

Discussion

In this particular investigation, we discovered that PA increased ROS in cardiomyocytes, damaged mitochondria, caused mitochondrial morphology and function changes, and affected NO production through the CRIF1/SIRT1/eNOS axis. Under normal conditions, NO is maintained at a certain level and exerts an inhibitory effect on neuron proliferation by inducing increased nitrosylation of Keap1 to promote transcriptional expression of Nrf2 into the nucleus agonist P21. In response to high-fat stimulation, the NO content decreased. Thus, its inhibitory effect on nerve cells is lost, and remodelling is induced. These effects increase susceptibility to AF. Finally, we found that TMZ supplementation attenuated high-fat-induced neural remodelling and reduced the induction of AF by promoting eNOS expression.

The cardiac autonomic nervous system (ANS) can be divided into extrinsic and intrinsic components[18, 19] and consists of both sympathetic and vagus nerves. The sympathetic nerve originates from the interior of the spinal column, and the vagus nerve originates from the medulla. Together, they form ganglia at the fat pad of the pulmonary vein orifice and regulate the balance between the extrinsic and intrinsic nervous systems of the heart[19]. Joint sympathetic-vagal activation constitutes the substrate for AF episodes[20]. Disturbances in the activity of the intrinsic nerves of the heart have been shown to enhance AF of pulmonary venous origin[21, 22], whereas elimination of the autonomic nerves can suppress AF episodes from the pulmonary veins[23]. The activation of the ANS is associated with many of the risk factors for AF, which may be associated with altered atrial electrophysiology with excessive regeneration and inhomogeneity of distribution of nerves[24]. Intrinsic autonomic modulation can also be the sole trigger for AF[25]. Vagal activity enhances acetylcholine-dependent K currents[26]. β-adrenergic receptor activation promotes Ca2+ inwards flow and facilitates DAD-related ectopic firing by hyperphosphorylation of RyR2[27]. In dogs with AF simulated by rapid atrial pacing, there is atrial regeneration with hyperinnervation[28]. In our experiments, we confirmed the presence of abnormal nerve proliferation and over-innervation in the atria of rats in the HFD group. Additionally, the AERP was shortened, and the induction of AF was increased.

In the present study, we found that high-fat stimulation impaired mitochondrial morphological functions, and the ATP content was decreased in cardiomyocytes. Due to the high energy demand of the heart, mitochondria are highly present in cardiomyocytes and provide large amounts of energy through oxidative phosphorylation[29]. Some studies have confirmed that mitochondrial complexes are damaged in the atrial tissue of patients with AF[30]. A dysfunctional mitochondrial system leads to an overabundance of reactive oxygen species (ROS), which eventually leads to oxidative stress[31]. Dysfunctional mitochondria affect cellular respiration and excess ROS are generated during energy production, causing oxidative stress[32]. Maintaining normal physiological functions requires a specific amount of ROS, however, an excess of ROS can cause oxidative harm to proteins, lipids, and nucleic acids[33]. This exacerbates mitochondrial damage and creates a vicious cycle[34]. In AF, the atria undergo rapid and irregular beating, which increases energy demand. Damage to mitochondrial function can lead to an untimely energy supply to the atrial muscle, further aggravating the injury to cardiac function. In addition, when mitochondrial damage occurs, oxidative phosphate does not proceed correctly, which can contribute to cellular metabolism shifting towards glycolysis[35, 36]. The adult heart derives its energy primarily from fatty acid oxidation. When the energy supply mode is switched from fatty acid β-oxidation to glycolysis, increased lactate production leads to acidosis. Excessive acidosis results in an overload of Na+ due to the exchange of Na+-H+ and subsequently leads to an overload of Ca+. This can be secondary to cardiac electrical remodelling and promote the development and progression of AF[37].

This study confirms that high fatty acid stimulation reduces cardiomyocyte NO production. NO, a diffusible and highly reactive free radical, is produced by NOS with arginine as substrate under the action of cofactor BH4[10]. NOS has three isoforms: nNOS (neuronal NOS), iNOS (inducible NOS), and eNOS (endothelial NOS). nNOS and eNOS are constitutively expressed in the myocardium, and iNOS is expressed only during inflammatory or pathological states[38]. Studies have demonstrated that the SIRT1/eNOS pathway has a beneficial effect in conditions like ischemia reperfusion[39, 40], atherosclerotic processes[41], and cardiomyopathy[42]. We verified that high-fat stimulation also affects NO production through the SIRT1/eNOS axis. ROS can regulate SIRT1 expression[43], and we reversed the decrease in protein expression of SIRT1 and eNOS caused by high-fat stimulation after ROS removal with NAC. CRIF1 can affect mitochondrial respiratory chain oxidative phosphorylation, thereby regulating ROS production[44]. Our previous experiments revealed that high-fat stimulation could damage the mitochondrial respiratory chain in cardiomyocytes. Therefore, we further verified the relationship between CRIF1 and the SIRT1/eNOS axis. The results suggest that CRIF1 has a role in controlling the acetylation of eNOS through the regulation of SIRT1 expression in response to PA stimulation, thereby influencing the production of NO.

PC12 is a cell line derived from rat adrenal tissue that can exhibit multiple functions of primary neurons and has been widely used as a neuronal model[45]. We established a coculture system using H9C2 cardiomyocytes and PC12 neuronal cells in a high-fat environment to verify the interaction between cardiomyocytes and neuronal cells under high-fat stimulation. We found that high-fat stimulation increased neuronal cell proliferation. In contrast, overexpression of eNOS in cardiomyocytes inhibited neuronal cell proliferation.

Several studies have shown that NO negatively regulates cell proliferation[46-48], As a result, we speculated that NO produced by cardiomyocytes inhibits neuronal cell proliferation Therefore, we added NO scavengers to the culture medium after overexpression of eNOS in cardiomyocytes, and the inhibition of neuronal cell proliferation after overexpression of cardiomyocyte eNOS was partially counteracted. This finding further confirms that NO plays a bridging role in the high lipid stimulation of cardiomyocytes to affect neuronal cell remodelling.

NO, a typical gas signalling molecule, activates guanylate cyclase (sGC). sGC produces cGMP and activates cGMP-dependent protein kinase (PKG)[49]. Recently, a growing body of research has verified that nitrogen monoxide can chemically attach to the sulfur-containing compound cysteine, resulting in the formation of S-nitrosothiols, which is referred to as S-nitrosylation.[50]. This reversible and ubiquitous post-translational modification of proteins regulates various biological activities[12] and is also regulated by enzymatic degradation. The regulation of S-nitrosylation is significantly influenced by GSNOR, which is an important factor in multiple cardiovascular disorders and can exert anti-inflammatory effects by controlling endothelial protein transport and inhibiting the expression of proinflammatory factors[51]. Oestrogen can exert cardioprotective effects by nitrosylating mitochondria-associated proteins[52]. In addition to the protective effects, one study confirmed that SNO-Hsp90 could exacerbate cardiac hypertrophy[53]. This suggests that S-nitrosylation is a double-edged sword that can exert cardioprotective impacts and exacerbate myocardial damage. The present study confirmed that NO could inhibit neuronal cell proliferation, but the exact underlying mechanism of this effect remains to be further explored.

P21$^{WAF1/CIP1}$ is a broadly acting cell cycle protein-dependent kinase inhibitor that can be regulated by various transcription factors. Maintaining its stability is crucial for the correct progression of the cell cycle and the determination of cellular outcomes. [54]. Earlier investigations have indicated that P21 has the capacity to impede the cell cycle progression in diverse types of tumor cells[55] and suppress the process of skeletal muscle and bone tissue regeneration[56]. P21 also exerts some inhibitory effects on proliferation in mammalian cardiac tissue[57, 58]. The prior research substantiated that in neuronal cells, the expression of P21 could be elevated through the influence of NO, thereby inhibiting proliferation. We predicted transcription factors based on the promoter of P21. Our discovery reveals that Nrf2 effectively binds to the P21 promoter, thereby exerting precise control over the transcriptional regulation of P21. Nrf2, a transcription factor expressed in nearly all tissues, plays a crucial role in the body's antioxidative processes[59, 60]. Our previous study revealed that Nrf2 could regulate the proliferation of fibroblasts. Therefore, we hypothesized that NO regulates the expression of P21 through Nrf2. Consequently, we applied SNAP to increase NO in cultured neuronal cells. In a dose-dependent manner, we observed that SNAP enhanced the protein levels of Nrf2 and P21. Additionally, the protein expression of P21 declined upon Nrf2 inhibition through siRNA.

The expression of Nrf2 is tightly regulated in multiple ways, and Keap1, a major regulator of Nrf2[61], is a zinc finger protein consisting of 624 amino acids with a high cysteine content, forming a homodimer[62]. At the physiological level, Keap1 engages with Nrf2 within the cytoplasm, facilitating the ubiquitination and subsequent degradation of Nrf2,

thereby maintaining Nrf2 at a reduced concentration[63]. When stimulated, Keap1 dissociates from Nrf2 to allow its entry into the nucleus to produce an effect[64]. S-nitrosylation is a post-translational protein modification, which is the reaction of endogenous NO with thiols of cysteine to form SNO. It has a crucial role in maintaining cardiac function and regulating oxidative stress homeostasis[65]. Keap1 is rich in cysteine, providing a potential target for regulating thiol-responsive chemicals[66]. We verified in vitro that NO could regulate neuronal cell proliferation by increasing Keap1 nitrosylation and thus Nrf2 entry into the nucleus to increase agonistic P21 transcription.

TMZ is a cardioprotective drug that improves myocardial function by modifying the metabolism of myocardial energy[67]. It has been observed that TMZ exhibits a protective effect in patients with angina pectoris, post-stenting, and heart failure[68]; Because it lacks significant side effects, it has been widely applied in clinical practice. [69]. TMZ reduces oxidative stress and improves myocardial ultrastructural remodelling in dogs with AF by activating eNOS. This reduces the rate of AF induction and duration of AF[70]. It has been reported that TMZ may exert antioxidant effects and improve cardiac function by increasing the expression of eNOS[71, 72]. In our study, we constructed a high-fat environmental coculture model and the HFD rat model and verified that TMZ could partially reverse high-fat-induced atrial neural remodelling by restoring myocardial eNOS expression and NO content. TMZ supplementation may be contemplated as part of the treatment regimen for high-risk patients with elevated lipid levels. However, it is essential to emphasize that further research is necessary to fully elucidate its effectiveness. Our study provides a foundation for the potential investigation of TMZ as an adjunct therapy for cardiac protection in high-lipid patients, with the recognition that more extensive investigation is needed to validate its efficacy.

Strengths and limitation

This study effectively validates the significance of the CRIF1/eNOS/P21 pathway in the context of atrial neural remodeling triggered by high-fat stimulation. Additionally, it firmly establishes the impact of TMZ on the modulation of atrial neural remodeling resulting from high-fat stimulation. These findings provide valuable new perspectives that could potentially contribute to the advancement of clinical approaches for managing atrial fibrillation.

Conclusions

In the present study, we found that high-fat stimulation damaged cardiomyocyte mitochondria, affected mitochondrial morphology and function, and decreased NO production through the CRIF1/SIRT1/eNOS axis, thereby allowing neuronal cell proliferation and leading to remodelling. Increasing NO levels increased Keap1 nitrosylation, promoting Nrf2 entry into the nucleus to activate P21 expression. Therefore, neuronal cell proliferation is inhibited in the high-fat stimulated myocardial microenvironment. TMZ inhibits neural remodelling caused by high-fat stimulation by increasing the protein expression of eNOS and decreasing the rate of AF induction. Therefore, we believe that the addition of quetiapine for myocardial protection may be

considered when treating high-risk patients with elevated lipid levels. However, further research is needed to substantiate its effectiveness.

References

1. Abed HS, Samuel CS, Lau DH, Kelly DJ, Royce SG, Alasady M, Mahajan R, Kuklik P, Zhang Y, Brooks AG *et al*: **Obesity results in progressive atrial structural and electrical remodeling: implications for atrial fibrillation**. *Heart Rhythm* 2013, **10**(1):90-100.
2. Schnabel RB, Yin X, Gona P, Larson MG, Beiser AS, McManus DD, Newton-Cheh C, Lubitz SA, Magnani JW, Ellinor PT *et al*: **50 year trends in atrial fibrillation prevalence, incidence, risk factors, and mortality in the Framingham Heart Study: a cohort study**. *Lancet* 2015, **386**(9989):154-162.
3. Iwasaki YK, Nishida K, Kato T, Nattel S: **Atrial fibrillation pathophysiology: implications for management**. *Circulation* 2011, **124**(20):2264-2274.
4. Yao YS, Li TD, Zeng ZH: **Mechanisms underlying direct actions of hyperlipidemia on myocardium: an updated review**. *Lipids Health Dis* 2020, **19**(1):23.
5. Han Q, Yeung SC, Ip MSM, Mak JCW: **Dysregulation of cardiac lipid parameters in high-fat high-cholesterol diet-induced rat model**. *Lipids Health Dis* 2018, **17**(1):255.
6. Cheng Y, Zhu Y, Zhang J, Duan X, Zhang Y: **Large Accumulation of Collagen and Increased Activation of Mast Cells in Hearts of Mice with Hyperlipidemia**. *Arq Bras Cardiol* 2017, **109**(5):404-409.
7. Joseph LC, Subramanyam P, Radlicz C, Trent CM, Iyer V, Colecraft HM, Morrow JP: **Mitochondrial oxidative stress during cardiac lipid overload causes intracellular calcium leak and arrhythmia**. *Heart Rhythm* 2016, **13**(8):1699-1706.
8. Zorov DB, Juhaszova M, Sollott SJ: **Mitochondrial reactive oxygen species (ROS) and ROS-induced ROS release**. *Physiol Rev* 2014, **94**(3):909-950.
9. Droge W: **Free radicals in the physiological control of cell function**. *Physiol Rev* 2002, **82**(1):47-95.
10. Karam BS, Chavez-Moreno A, Koh W, Akar JG, Akar FG: **Oxidative stress and inflammation as central mediators of atrial fibrillation in obesity and diabetes**. *Cardiovasc Diabetol* 2017, **16**(1):120.
11. Forstermann U: **Nitric oxide and oxidative stress in vascular disease**. *Pflugers Arch* 2010, **459**(6):923-939.
12. Ahmad A, Dempsey SK, Daneva Z, Azam M, Li N, Li PL, Ritter JK: **Role of Nitric Oxide in the Cardiovascular and Renal Systems**. *Int J Mol Sci* 2018, **19**(9).
13. Lima B, Forrester MT, Hess DT, Stamler JS: **S-nitrosylation in cardiovascular signaling**. *Circ Res* 2010, **106**(4):633-646.
14. Lenaerts I, Driesen RB, Hermida N, Holemans P, Heidbuchel H, Janssens S, Balligand JL, Sipido KR, Willems R: **Role of nitric oxide and oxidative stress in a sheep model of persistent atrial fibrillation**. *Europace* 2013, **15**(5):754-760.
15. Cai H, Li Z, Goette A, Mera F, Honeycutt C, Feterik K, Wilcox JN, Dudley SC, Jr., Harrison DG, Langberg JJ: **Downregulation of endocardial nitric oxide synthase expression and nitric oxide production in atrial fibrillation: potential mechanisms for atrial thrombosis and stroke**. *Circulation* 2002, **106**(22):2854-2858.
16. Ciani E, Severi S, Contestabile A, Bartesaghi R, Contestabile A: **Nitric oxide negatively regulates proliferation and promotes neuronal differentiation through N-Myc downregulation**. *J Cell Sci* 2004, **117**(Pt 20):4727-4737.
17. Wei J, Zhang Y, Li Z, Wang X, Chen L, Du J, Liu J, Liu J, Hou Y: **GCH1 attenuates cardiac autonomic

nervous remodeling in canines with atrial-tachypacing via tetrahydrobiopterin pathway regulated by microRNA-206. *Pacing Clin Electrophysiol* 2018, **41**(5):459-471.

18. Armour JA: **Functional anatomy of intrathoracic neurons innervating the atria and ventricles**. *Heart Rhythm* 2010, **7**(7):994-996.
19. Hou Y, Scherlag BJ, Lin J, Zhang Y, Lu Z, Truong K, Patterson E, Lazzara R, Jackman WM, Po SS: **Ganglionated plexi modulate extrinsic cardiac autonomic nerve input: effects on sinus rate, atrioventricular conduction, refractoriness, and inducibility of atrial fibrillation**. *J Am Coll Cardiol* 2007, **50**(1):61-68.
20. Schotten U, Verheule S, Kirchhof P, Goette A: **Pathophysiological mechanisms of atrial fibrillation: a translational appraisal**. *Physiol Rev* 2011, **91**(1):265-325.
21. Patterson E, Po SS, Scherlag BJ, Lazzara R: **Triggered firing in pulmonary veins initiated by in vitro autonomic nerve stimulation**. *Heart Rhythm* 2005, **2**(6):624-631.
22. Patterson E, Lazzara R, Szabo B, Liu H, Tang D, Li YH, Scherlag BJ, Po SS: **Sodium-calcium exchange initiated by the Ca2+ transient: an arrhythmia trigger within pulmonary veins**. *J Am Coll Cardiol* 2006, **47**(6):1196-1206.
23. Lu Z, Scherlag BJ, Lin J, Yu L, Guo JH, Niu G, Jackman WM, Lazzara R, Jiang H, Po SS: **Autonomic mechanism for initiation of rapid firing from atria and pulmonary veins: evidence by ablation of ganglionated plexi**. *Cardiovasc Res* 2009, **84**(2):245-252.
24. Linz D, Ukena C, Mahfoud F, Neuberger HR, Bohm M: **Atrial autonomic innervation: a target for interventional antiarrhythmic therapy?** *J Am Coll Cardiol* 2014, **63**(3):215-224.
25. Park HW, Shen MJ, Lin SF, Fishbein MC, Chen LS, Chen PS: **Neural mechanisms of atrial fibrillation**. *Curr Opin Cardiol* 2012, **27**(1):24-28.
26. Kneller J, Zou R, Vigmond EJ, Wang Z, Leon LJ, Nattel S: **Cholinergic atrial fibrillation in a computer model of a two-dimensional sheet of canine atrial cells with realistic ionic properties**. *Circ Res* 2002, **90**(9):E73-87.
27. Dobrev D, Voigt N, Wehrens XH: **The ryanodine receptor channel as a molecular motif in atrial fibrillation: pathophysiological and therapeutic implications**. *Cardiovasc Res* 2011, **89**(4):734-743.
28. Tan AY, Zhou S, Ogawa M, Song J, Chu M, Li H, Fishbein MC, Lin SF, Chen LS, Chen PS: **Neural mechanisms of paroxysmal atrial fibrillation and paroxysmal atrial tachycardia in ambulatory canines**. *Circulation* 2008, **118**(9):916-925.
29. Hall CJ, Sanderson LE, Crosier KE, Crosier PS: **Mitochondrial metabolism, reactive oxygen species, and macrophage function-fishing for insights**. *J Mol Med (Berl)* 2014, **92**(11):1119-1128.
30. Emelyanova L, Ashary Z, Cosic M, Negmadjanov U, Ross G, Rizvi F, Olet S, Kress D, Sra J, Tajik AJ *et al*: **Selective downregulation of mitochondrial electron transport chain activity and increased oxidative stress in human atrial fibrillation**. *Am J Physiol Heart Circ Physiol* 2016, **311**(1):H54-63.
31. Lenaz G, Genova ML: **Structure and organization of mitochondrial respiratory complexes: a new understanding of an old subject**. *Antioxid Redox Signal* 2010, **12**(8):961-1008.
32. Poznyak AV, Ivanova EA, Sobenin IA, Yet SF, Orekhov AN: **The Role of Mitochondria in Cardiovascular Diseases**. *Biology (Basel)* 2020, **9**(6).
33. Camara AK, Lesnefsky EJ, Stowe DF: **Potential therapeutic benefits of strategies directed to mitochondria**. *Antioxid Redox Signal* 2010, **13**(3):279-347.
34. Szczepanowska K, Trifunovic A: **Origins of mtDNA mutations in ageing**. *Essays Biochem* 2017, **61**(3):325-337.
35. Fu Y, Wang D, Wang H, Cai M, Li C, Zhang X, Chen H, Hu Y, Zhang X, Ying M *et al*: **TSPO deficiency**

induces mitochondrial dysfunction, leading to hypoxia, angiogenesis, and a growth-promoting metabolic shift toward glycolysis in glioblastoma. *Neuro Oncol* 2020, **22**(2):240-252.
36. Smith AM, Depp C, Ryan BJ, Johnston GI, Alegre-Abarrategui J, Evetts S, Rolinski M, Baig F, Ruffmann C, Simon AK *et al*: **Mitochondrial dysfunction and increased glycolysis in prodromal and early Parkinson's blood cells**. *Mov Disord* 2018, **33**(10):1580-1590.
37. Yang KC, Dudley SC, Jr.: **Oxidative stress and atrial fibrillation: finding a missing piece to the puzzle**. *Circulation* 2013, **128**(16):1724-1726.
38. Nishijima Y, Sridhar A, Bonilla I, Velayutham M, Khan M, Terentyeva R, Li C, Kuppusamy P, Elton TS, Terentyev D *et al*: **Tetrahydrobiopterin depletion and NOS2 uncoupling contribute to heart failure-induced alterations in atrial electrophysiology**. *Cardiovasc Res* 2011, **91**(1):71-79.
39. Li D, Wang X, Huang Q, Li S, Zhou Y, Li Z: **Cardioprotection of CAPE-oNO(2) against myocardial ischemia/reperfusion induced ROS generation via regulating the SIRT1/eNOS/NF-kappaB pathway in vivo and in vitro**. *Redox Biol* 2018, **15**:62-73.
40. Ding M, Lei J, Han H, Li W, Qu Y, Fu E, Fu F, Wang X: **SIRT1 protects against myocardial ischemia-reperfusion injury via activating eNOS in diabetic rats**. *Cardiovasc Diabetol* 2015, **14**:143.
41. Luo Y, Lu S, Ai Q, Zhou P, Qin M, Sun G, Sun X: **SIRT1/AMPK and Akt/eNOS signaling pathways are involved in endothelial protection of total aralosides of Aralia elata (Miq) Seem against high-fat diet-induced atherosclerosis in ApoE-/- mice**. *Phytother Res* 2019, **33**(3):768-778.
42. Karbasforooshan H, Karimi G: **The role of SIRT1 in diabetic cardiomyopathy**. *Biomed Pharmacother* 2017, **90**:386-392.
43. Palmeira CM, Teodoro JS, Amorim JA, Steegborn C, Sinclair DA, Rolo AP: **Mitohormesis and metabolic health: The interplay between ROS, cAMP and sirtuins**. *Free Radic Biol Med* 2019, **141**:483-491.
44. Chang H, Li J, Qu K, Wan Y, Liu S, Zheng W, Zhang Z, Liu C: **CRIF1 overexpression facilitates tumor growth and metastasis through inducing ROS/NFkappaB pathway in hepatocellular carcinoma**. *Cell Death Dis* 2020, **11**(5):332.
45. Zhang C, Li C, Chen S, Li Z, Jia X, Wang K, Bao J, Liang Y, Wang X, Chen M *et al*: **Berberine protects against 6-OHDA-induced neurotoxicity in PC12 cells and zebrafish through hormetic mechanisms involving PI3K/AKT/Bcl-2 and Nrf2/HO-1 pathways**. *Redox Biol* 2017, **11**:1-11.
46. Dai Y, Zhu Y, Cheng J, Shen J, Huang H, Liu M, Chen Z, Liu Y: **Nitric oxide-releasing platinum(IV) prodrug efficiently inhibits proliferation and metastasis of cancer cells**. *Chem Commun (Camb)* 2020, **56**(90):14051-14054.
47. Maksoud MJE, Tellios V, Xiang YY, Lu WY: **Nitric oxide signaling inhibits microglia proliferation by activation of protein kinase-G**. *Nitric Oxide* 2020, **94**:125-134.
48. Bruno CJ, Greco TM, Ischiropoulos H: **Nitric oxide counteracts the hyperoxia-induced proliferation and proinflammatory responses of mouse astrocytes**. *Free Radic Biol Med* 2011, **51**(2):474-479.
49. Ataei Ataabadi E, Golshiri K, Juttner A, Krenning G, Danser AHJ, Roks AJM: **Nitric Oxide-cGMP Signaling in Hypertension: Current and Future Options for Pharmacotherapy**. *Hypertension* 2020, **76**(4):1055-1068.
50. Hess DT, Stamler JS: **Regulation by S-nitrosylation of protein post-translational modification**. *J Biol Chem* 2012, **287**(7):4411-4418.
51. Lowenstein CJ: **Nitric oxide regulation of protein trafficking in the cardiovascular system**. *Cardiovasc Res* 2007, **75**(2):240-246.
52. Sun J, Morgan M, Shen RF, Steenbergen C, Murphy E: **Preconditioning results in S-nitrosylation of**

proteins involved in regulation of mitochondrial energetics and calcium transport. *Circ Res* 2007, **101**(11):1155-1163.

53. Zhao S, Song TY, Wang ZY, Gao J, Cao JW, Hu LL, Huang ZR, Xie LP, Ji Y: **S-nitrosylation of Hsp90 promotes cardiac hypertrophy in mice through GSK3beta signaling**. *Acta Pharmacol Sin* 2022, **43**(8):1979-1988.
54. Deng T, Yan G, Song X, Xie L, Zhou Y, Li J, Hu X, Li Z, Hu J, Zhang Y *et al*: **Deubiquitylation and stabilization of p21 by USP11 is critical for cell-cycle progression and DNA damage responses**. *Proc Natl Acad Sci U S A* 2018, **115**(18):4678-4683.
55. El-Deiry WS: **p21(WAF1) Mediates Cell-Cycle Inhibition, Relevant to Cancer Suppression and Therapy**. *Cancer Res* 2016, **76**(18):5189-5191.
56. Chinzei N, Hayashi S, Ueha T, Fujishiro T, Kanzaki N, Hashimoto S, Sakata S, Kihara S, Haneda M, Sakai Y *et al*: **P21 deficiency delays regeneration of skeletal muscular tissue**. *PLoS One* 2015, **10**(5):e0125765.
57. Aix E, Gutierrez-Gutierrez O, Sanchez-Ferrer C, Aguado T, Flores I: **Postnatal telomere dysfunction induces cardiomyocyte cell-cycle arrest through p21 activation**. *J Cell Biol* 2016, **213**(5):571-583.
58. Tane S, Ikenishi A, Okayama H, Iwamoto N, Nakayama KI, Takeuchi T: **CDK inhibitors, p21(Cip1) and p27(Kip1), participate in cell cycle exit of mammalian cardiomyocytes**. *Biochem Biophys Res Commun* 2014, **443**(3):1105-1109.
59. Ma Q: **Role of nrf2 in oxidative stress and toxicity**. *Annu Rev Pharmacol Toxicol* 2013, **53**:401-426.
60. Tan Y, Ichikawa T, Li J, Si Q, Yang H, Chen X, Goldblatt CS, Meyer CJ, Li X, Cai L *et al*: **Diabetic downregulation of Nrf2 activity via ERK contributes to oxidative stress-induced insulin resistance in cardiac cells in vitro and in vivo**. *Diabetes* 2011, **60**(2):625-633.
61. Zang H, Mathew RO, Cui T: **The Dark Side of Nrf2 in the Heart**. *Front Physiol* 2020, **11**:722.
62. Tu W, Wang H, Li S, Liu Q, Sha H: **The Anti-Inflammatory and Anti-Oxidant Mechanisms of the Keap1/Nrf2/ARE Signaling Pathway in Chronic Diseases**. *Aging Dis* 2019, **10**(3):637-651.
63. Suzuki T, Yamamoto M: **Molecular basis of the Keap1-Nrf2 system**. *Free Radic Biol Med* 2015, **88**(Pt B):93-100.
64. Baird L, Dinkova-Kostova AT: **The cytoprotective role of the Keap1-Nrf2 pathway**. *Arch Toxicol* 2011, **85**(4):241-272.
65. Shi X, Qiu H: **Post-Translational S-Nitrosylation of Proteins in Regulating Cardiac Oxidative Stress**. *Antioxidants (Basel)* 2020, **9**(11).
66. Nguyen T, Nioi P, Pickett CB: **The Nrf2-antioxidant response element signaling pathway and its activation by oxidative stress**. *J Biol Chem* 2009, **284**(20):13291-13295.
67. Kantor PF, Lucien A, Kozak R, Lopaschuk GD: **The antianginal drug trimetazidine shifts cardiac energy metabolism from fatty acid oxidation to glucose oxidation by inhibiting mitochondrial long-chain 3-ketoacyl coenzyme A thiolase**. *Circ Res* 2000, **86**(5):580-588.
68. Tarkin JM, Kaski JC: **Trimetazidine: is there a role beyond angina?** *Eur Heart J Cardiovasc Pharmacother* 2018, **4**(2):67-68.
69. Marzilli M, Vinereanu D, Lopaschuk G, Chen Y, Dalal JJ, Danchin N, Etriby E, Ferrari R, Gowdak LH, Lopatin Y *et al*: **Trimetazidine in cardiovascular medicine**. *Int J Cardiol* 2019, **293**:39-44.
70. Li Z, Chaolan L, Chengcheng W, Xi H, Yingying W, Jiaqiu L, Wei H: **GW28-e0789 Trimetazidine decreases inducibility and duration of atrial fibrillation in a dog model of congestive heart failure**. *Journal of the American College of Cardiology* 2017, **70**(16S):C29-C29.
71. Wu Q, Qi B, Liu Y, Cheng B, Liu L, Li Y, Wang Q: **Mechanisms underlying protective effects of**

trimetazidine on endothelial progenitor cells biological functions against H2O2-induced injury: involvement of antioxidation and Akt/eNOS signaling pathways. *Eur J Pharmacol* 2013, **707**(1-3):87-94.

72. Di Napoli P, Chierchia S, Taccardi AA, Grilli A, Felaco M, De Caterina R, Barsotti A: **Trimetazidine improves post-ischemic recovery by preserving endothelial nitric oxide synthase expression in isolated working rat hearts**. *Nitric Oxide* 2007, **16**(2):228-236.

A PROMO database

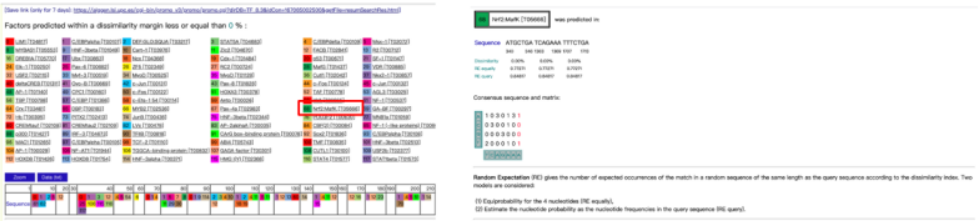

B JASPAR database

Matrix ID	Name	Score	Relative score	Sequence ID	Start	End	Strand	[illegible] sequence
MA0150.1	MA0150.1.NFE2L2	9.392886	0.8351686320129837	NC_000006.12:36674463-36676562	188	198	-	ATGGCTGAGCA
MA0150.1	MA0150.1.NFE2L2	9.27905	0.832339156215503	NC_000006.12:36674463-36676562	663	673	+	GTGACTCATCC

Supplementary Figure 1. P21 promoter region has Nrf2 binding sites.

(A) PROMO database predictes Nrf2 binding sites in the P21 promoter region.

(B) JASPER database predictes Nrf2 binding sites in the P21 promoter region.

20230910131438898237097525014528

ORIGINALITY REPORT

13%
SIMILARITY INDEX

PRIMARY SOURCES

1	Systems Biology of Free Radicals and Antioxidants, 2014.<br>Crossref	53 words — 1%
2	KURAL, Birgül Vanizor, ALVER, Ahmet, CANPOLAT, Sinan, KAHRAMAN, Cemil, ALTAY, Diler Us, KARA, Hanife and AKCAN, Buket. "Sıçanlarda N-asetilsistein takviyeli ve takviyesiz yüksek yağlı diyetin serum ve", Türk Biyokimya Derneği, 2014.<br>Publications	40 words — 1%
3	www.nature.com<br>Internet	35 words — < 1%
4	link.springer.com<br>Internet	34 words — < 1%
5	www.frontiersin.org<br>Internet	32 words — < 1%
6	topsecretapiaccess.dovepress.com<br>Internet	28 words — < 1%
7	www.mdpi.com<br>Internet	28 words — < 1%
8	www.spandidos-publications.com<br>Internet	25 words — < 1%

9 docksci.com
Internet
23 words — < 1%

10 www.researchsquare.com
Internet
22 words — < 1%

11 nutrition.moh.gov.my
Internet
19 words — < 1%

12 stemcellres.biomedcentral.com
Internet
18 words — < 1%

13 worldwidescience.org
Internet
18 words — < 1%

14 www.thno.org
Internet
18 words — < 1%

15 Tamami Kawasaki, Masanobu Sakata, Hideo Namiki. "Elemental Characterization of Daphnia Resting Eggs by X-ray Analytical Microscopy", Zoological Science, 2004
Crossref
17 words — < 1%

16 Kamila Lievajová, Marcela Martončíková, Juraj Blaško, Judita Orendáčová, Viera Almašiová, Enikő Račeková. "Early stress affects neurogenesis in the rat rostral migratory stream", Open Life Sciences, 2010
Crossref
16 words — < 1%

17 Stefano R. Giannubilo, Marta Menegazzi, Elisa Tedeschi, Valeria Bezzeccheri, Hisanori Suzuki, Andrea L. Tranquilli. "Doppler analysis and placental nitric oxide synthase expression during fetal growth restriction", The Journal of Maternal-Fetal & Neonatal Medicine, 2009
Crossref
16 words — < 1%

18	Wenwen Xia, Zhaomin Lu, Wei Chen, Jianjun Zhou, Yan Zhao. "Excess fatty acids induce pancreatic acinar cell pyroptosis through macrophage M1 polarization", BMC Gastroenterology, 2022<br>Crossref	16 words — < 1%
19	www.medrxiv.org<br>Internet	16 words — < 1%
20	"Redox-Active Therapeutics", Springer Nature, 2016<br>Crossref	15 words — < 1%
21	academic.oup.com<br>Internet	15 words — < 1%
22	cancerci.biomedcentral.com<br>Internet	15 words — < 1%
23	www.ncbi.nlm.nih.gov<br>Internet	15 words — < 1%
24	Chang, Huai-Chia, Yu-Ting Tai, Yih-Giun Cherng, Jia-Wei Lin, Shing-Hwa Liu, Ta-Liang Chen, and Ruei-Ming Chen. "Resveratrol Attenuates High-Fat Diet-Induced Disruption of the Blood–Brain Barrier and Protects Brain Neurons from Apoptotic Insults", Journal of Agricultural and Food Chemistry<br>Crossref	14 words — < 1%
25	Losby, McKenna. "A Mechanistic Study of ERR α/γ Agonists for Treatment of Metabolic Dysfunction in Heart Failure", Washington University in St. Louis, 2023<br>ProQuest	14 words — < 1%
26	www.bio-rad-antibodies.com<br>Internet	14 words — < 1%

27	Mu Qin, Cong Zeng, Xu Liu. "The cardiac autonomic nervous system: A target for modulation of atrial fibrillation", Clinical Cardiology, 2019<br>Crossref	13 words — < 1%
28	Ulkan Kilic, Ozlem Gok, Ufuk Erenberk, Mehmet Rusen Dundaroz et al. "A Remarkable Age-Related Increase in SIRT1 Protein Expression against Oxidative Stress in Elderly: SIRT1 Gene Variants and Longevity in Human", PLOS ONE, 2015<br>Crossref	13 words — < 1%
29	Jing-Xian Wang, Yuan Yang, Wen-Ying Li. "SIRT3 deficiency increases mitochondrial oxidative stress and promotes migration of retinal pigment epithelial cells", Experimental Biology and Medicine, 2021<br>Crossref	12 words — < 1%
30	www.dovepress.com<br>Internet	11 words — < 1%
31	www.explorationpub.com<br>Internet	11 words — < 1%
32	www.hsc.unt.edu<br>Internet	11 words — < 1%
33	Jian Kang, Xu Huang, Weiguo Dong, Xueying Zhu, Ming Li, Ning Cui. "Long non-coding RNA LINC00630 facilitates hepatocellular carcinoma progression through recruiting transcription factor E2F1 to up-regulate cyclin-dependent kinase 2 expression", Human & Experimental Toxicology, 2021<br>Crossref	10 words — < 1%
34	Yanghong Xu, Xiaoyi Li, Jishuai Huang, Leilei Peng, Dinghui Luo, Qiannan Zhang, Zhiwu Dan,	10 words — < 1%

Haijun Xiao, Fang Yang, Jun Hu. "A simplified method to isolate rice mitochondria", Research Square Platform LLC, 2020
Crossref Posted Content

35 digital.library.adelaide.edu.au
Internet
10 words — < 1%

36 mafiadoc.com
Internet
10 words — < 1%

37 www.e-sc.org
Internet
10 words — < 1%

38 "Practical Guide to Catheter Ablation of Atrial Fibrillation", Wiley, 2015
Crossref
9 words — < 1%

39 Arshia Naaz, Yizhong Zhang, Nashrul Afiq Faidzinn, Sonia Yogasundaram, Mohammad Alfatah. "Curcumin extends the lifespan of aging postmitotic cells with mitochondrial dysfunction", Cold Spring Harbor Laboratory, 2023
Crossref Posted Content
9 words — < 1%

40 Lanfang Li, Junyu Mou, Yanwei Han, Min Wang, Shan Lu, Qiuxiao Ma, Jialu Wang, Jingxue Ye, Guibo Sun. "Calenduloside e modulates macrophage polarization via KLF2-regulated glycolysis, contributing to attenuates atherosclerosis", International Immunopharmacology, 2023
Crossref
9 words — < 1%

41 Probst, Brandon L., Lyndsey McCauley, Isaac Trevino, W. Christian Wigley, and Deborah A. Ferguson. "Cancer Cell Growth Is Differentially Affected by Constitutive Activation of NRF2 by KEAP1 Deletion and Pharmacological Activation of NRF2 by the Synthetic Triterpenoid, RTA 405", PLoS ONE, 2015.
Crossref
9 words — < 1%

42 Weiwei Li, Jiancheng Yang, Qiufeng Lyu, Gaofeng Wu, Shumei Lin, Qunhui Yang, Jianmin Hu. "Taurine attenuates isoproterenol-induced H9c2 cardiomyocytes hypertrophy by improving antioxidative ability and inhibiting calpain-1-mediated apoptosis", Molecular and Cellular Biochemistry, 2020 — 9 words — < 1%

Crossref

43 Yan Shi, Shang Wang, Ronghua Yang, Zhenmin Wang, Weiwei Zhang, Hongwei Liu, Yuesheng Huang. "ROS Promote Hypoxia-Induced Keratinocyte Epithelial-Mesenchymal Transition by Inducing SOX2 Expression and Subsequent Activation of Wnt/β-Catenin", Oxidative Medicine and Cellular Longevity, 2022 — 9 words — < 1%

Crossref

44 Zhihui Song, Rui Chen, Caijun Wang, Guiyun Pan, An Yan, Guinan Xie, Zhihua Yang, Wanying Feng, Yi Wang. "Effect and mechanism of Tangzhiqing in improving cardiac function in mice with hyperlipidaemia complicated with myocardial ischaemia", Heliyon, 2023 — 9 words — < 1%

Crossref

45 coek.info — 9 words — < 1%

Internet

46 jneuroinflammation.biomedcentral.com — 9 words — < 1%

Internet

47 mdpi-res.com — 9 words — < 1%

Internet

48 static-site-aging-prod2.impactaging.com — 9 words — < 1%

Internet

49 www.excli.de

Internet

9 words — < 1%

50 www.tandfonline.com
Internet

9 words — < 1%

51 Alexios S. Antonopoulos, Athina Goliopoulou, Evangelos Oikonomou, Sotiris Tsalamandris et al. "Redox State in Atrial Fibrillation Pathogenesis and Relevant Therapeutic Approaches", Current Medicinal Chemistry, 2019
Crossref

8 words — < 1%

52 Anastasios Spiliotopoulos, Lia Blokpoel Ferreras, Ruth M Densham, Simon G Caulton et al. "Discovery of peptide ligands targeting a specific ubiquitin-like domain-binding site in the deubiquitinase USP11", Journal of Biological Chemistry, 2018
Crossref

8 words — < 1%

53 Bin Huang, Chung Ling Liao, Ya Ping Lin, Shih Chung Chen, Danny Ling Wang. "S-nitrosoproteome in Endothelial Cells Revealed by a Modified Biotin Switch Approach Coupled with Western Blot-Based Two-Dimensional Gel Electrophoresis", Journal of Proteome Research, 2009
Crossref

8 words — < 1%

54 Dehao Shang, Minghao Huang, Biyao Wang, Xu Yan, Zhou Wu, Xinwen Zhang. "mtDNA Maintenance and Alterations in the Pathogenesis of Neurodegenerative Diseases", Current Neuropharmacology, 2023
Crossref

8 words — < 1%

55 Elżbieta Skrzydlewska, Wojciech Łuczaj, Michał Biernacki, Piotr Wójcik et al. "Preliminary Comparison of Molecular Antioxidant and Inflammatory

8 words — < 1%

Mechanisms Determined in the Peripheral Blood Granulocytes of COVID-19 Patients", International Journal of Molecular Sciences, 2023
Crossref

56 Fuqiang Liu, Xiaoqing Li, Han Yan, Jiao Wu, Yichun Yang, Jin He, Jun Chen, Zhongxiang Jiang, Fan Wu, Zheng Jiang. "Downregulation of CPT2 promotes proliferation and inhibits apoptosis through p53 pathway in colorectal cancer", Cellular Signalling, 2022 — 8 words — < 1%
Crossref

57 Hyo Jin Kang, Yong Weon Yi, Young Bin Hong, Hee Jeong Kim, Young-Joo Jang, Yeon-Sun Seong, Insoo Bae. "HER2 confers drug resistance of human breast cancer cells through activation of NRF2 by direct interaction", Scientific Reports, 2014 — 8 words — < 1%
Crossref

58 Junhui Sun. "Disruption of Caveolae Blocks Ischemic Preconditioning-Mediated S-Nitrosylation of Mitochondrial Proteins", Antioxidants and Redox Signaling, 08/11/2011 — 8 words — < 1%
Crossref

59 M. He. "Angiotensin II stimulates KLF5 phosphorylation and its interaction with c-Jun leading to suppression of p21 expression in vascular smooth muscle cells", Journal of Biochemistry, 07/23/2009 — 8 words — < 1%
Crossref

60 Mingxu Qi, Li He, Xiaofeng Ma, Zili Li. "MiR-181a-5p is involved in the cardiomyocytes apoptosis induced by hypoxia–reoxygenation through regulating SIRT1", Bioscience, Biotechnology, and Biochemistry, 2020 — 8 words — < 1%
Crossref

61 Qingmiao Shao, Lei Meng, Sharen Lee, Gary Tse, Mengqi Gong, Zhiwei Zhang, Jichao Zhao, Yungang Zhao, Guangping Li, Tong Liu. "Empagliflozin, a sodium glucose co-transporter-2 inhibitor, alleviates atrial remodeling and improves mitochondrial function in high-fat diet/streptozotocin-induced diabetic rats", Cardiovascular Diabetology, 2019 — 8 words — < 1%

Crossref

---

62 Sadagopan Magesh. "Small Molecule Modulators of Keap1-Nrf2-ARE Pathway as Potential Preventive and Therapeutic Agents : SMALL MOLECULE MODULATORS OF KEAP1-NRF2-ARE PATHWAY", Medicinal Research Reviews, 04/2012 — 8 words — < 1%

Crossref

---

63 Shuai Chen, Dianfu Ma, Suli Xiao, Pingping Li, Huaxiang Lei, Xiaojing Huang. "Effects of chronic apical periodontitis on the inflammatory response of the aorta in hyperlipemic rats", Clinical Oral Investigations, 2021 — 8 words — < 1%

Crossref

---

64 Su, Y.. "Substrate Inhibition of Nitric Oxide Synthase in Pulmonary Artery Endothelial Cells in Culture", Nitric Oxide, 199712 — 8 words — < 1%

Crossref

---

65 XiaoMeng Chen, LiLei Yu, ShaoBo Shi, Hong Jiang, CongXin Huang, Mayurika Desai, YiGang Li, Hector Barajas-Martinez, Dan Hu. " Neuronal Na 1.8 Channels as a Novel Therapeutic Target of Acute Atrial Fibrillation Prevention ", Journal of the American Heart Association, 2016 — 8 words — < 1%

Crossref

---

66 Yun-Han Jiang, Yu Zhu, Sai Chen, Hai-Long Wang, Yang Zhou, Fu-Qin Tang, Zhao Jian, Ying-Bin Xiao. "Re-enforcing hypoxia-induced polyploid cardiomyocytes enter — 8 words — < 1%

cytokinesis through activation of β-catenin", Scientific Reports, 2019
Crossref

67 Zhang, Yujiao, Shaohua Zheng, Yangyang Geng, Jiao Xue, Zhongsu Wang, Xinxing Xie, Jiangrong Wang, Shuyu Zhang, and Yinglong Hou. "MicroRNA Profiling of Atrial Fibrillation in Canines: MiR-206 Modulates Intrinsic Cardiac Autonomic Nerve Remodeling by Regulating SOD1", PLoS ONE, 2015.
Crossref
8 words — < 1%

68 Zheng Xu, Jian Sun, Qian Tong, Qian Lin, Lingbo Qian, Yongsoo Park, Yang Zheng. "The Role of ERK1/2 in the Development of Diabetic Cardiomyopathy", International Journal of Molecular Sciences, 2016
Crossref
8 words — < 1%

69 cardiab.biomedcentral.com
Internet
8 words — < 1%

70 dmsjournal.biomedcentral.com
Internet
8 words — < 1%

71 etheses.whiterose.ac.uk
Internet
8 words — < 1%

72 hdl.handle.net
Internet
8 words — < 1%

73 preview-ccforum.biomedcentral.com
Internet
8 words — < 1%

74 tel.archives-ouvertes.fr
Internet
8 words — < 1%

75 www.spkx.net.cn
Internet

8 words — < 1%

76 www.yumpu.com
Internet
8 words — < 1%

77 LINZ, DOMINIK, FELIX MAHFOUD, ULRICH SCHOTTEN, CHRISTIAN UKENA, HANS-RUPRECHT NEUBERGER, KLAUS WIRTH, and MICHAEL BÖHM. "Effects of Electrical Stimulation of Carotid Baroreflex and Renal Denervation on Atrial Electrophysiology : Renal Denervation and Baroreflex Stimulation", Journal of Cardiovascular Electrophysiology, 2013.
Crossref
7 words — < 1%

78 Mengqi Jiang, Yuanxiu Song, Xi Chen, Min Zhu, Wenjing Lu, Mingyu Wei, Feng Lan, Ming Cui, Yun Bai. "COX6A2 deficiency leads to cardiac remodeling in human pluripotent stem cell-derived cardiomyocytes", Research Square Platform LLC, 2022
Crossref Posted Content
7 words — < 1%

79 Ulkan Kilic, Ozlem Gok, Ahmet Bacaksiz, Muzeyyen Izmirli, Birsen Elibol-Can, Omer Uysal. "SIRT1 Gene Polymorphisms Affect the Protein Expression in Cardiovascular Diseases", PLoS ONE, 2014
Crossref
7 words — < 1%

80 "Brain and Heart Dynamics", Springer Science and Business Media LLC, 2020
Crossref
6 words — < 1%

81 Ankita Sarkar, Sourav Dutta, Malinki Sur, Semanti Chakraborty, Puja Dey, Piyali Mukherjee. " Early loss of endogenous following rotenone treatment leads to mitochondrial dysfunction and Sarm1 induction that is ameliorated by inhibition ", The FEBS Journal, 2022
6 words — < 1%

Crossref

82 Tao Wang, Jiahui Hou, Chang Su, Liang Zhao, Yijie Shi. "Hyaluronic acid-coated chitosan nanoparticles induce ROS-mediated tumor cell apoptosis and enhance antitumor efficiency by targeted drug delivery via CD44", Journal of Nanobiotechnology, 2017

Crossref

6 words — < 1%

EXCLUDE QUOTES	OFF	EXCLUDE SOURCES	OFF
EXCLUDE BIBLIOGRAPHY	ON	EXCLUDE MATCHES	OFF
