# Supplementary material for: High-fat stimulation induces atrial neural remodeling by reducing NO production via the CRIF1/eNOS/P21 axi
Source: Lipids Health Dis. 2023 Nov 6;22:189. doi: 10.1186/s12944-023-01952-7 (PMC10629039; doi:10.1186/s12944-023-01952-7)
Supplement: Supplementary file 4 — Supplementary Material 4 [file 12944_2023_1952_MOESM4_ESM.pdf]

WB ROW DATA

Figure2A

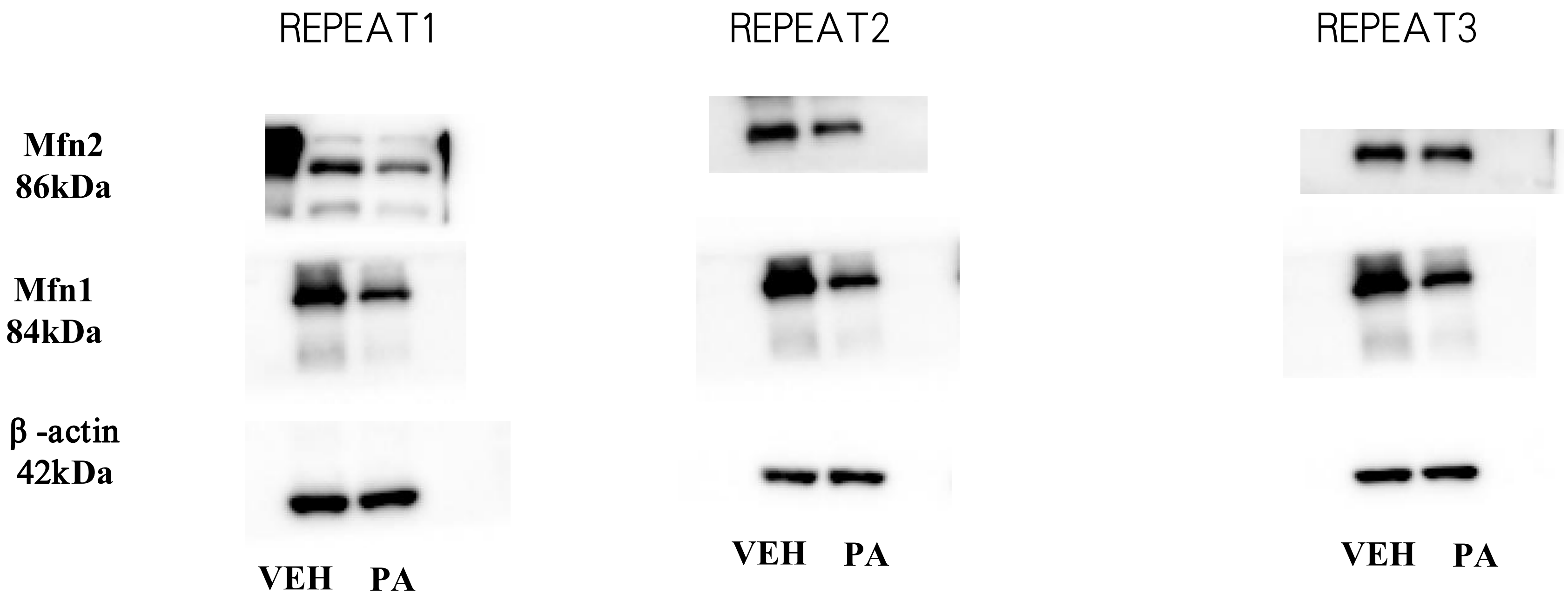
REPEAT1
REPEAT2
REPEAT3
Mfn2
86kDa
Mfn1
84kDa
β -actin
42kDa
VEH PA
VEH PA
VEH PA

Figure2C

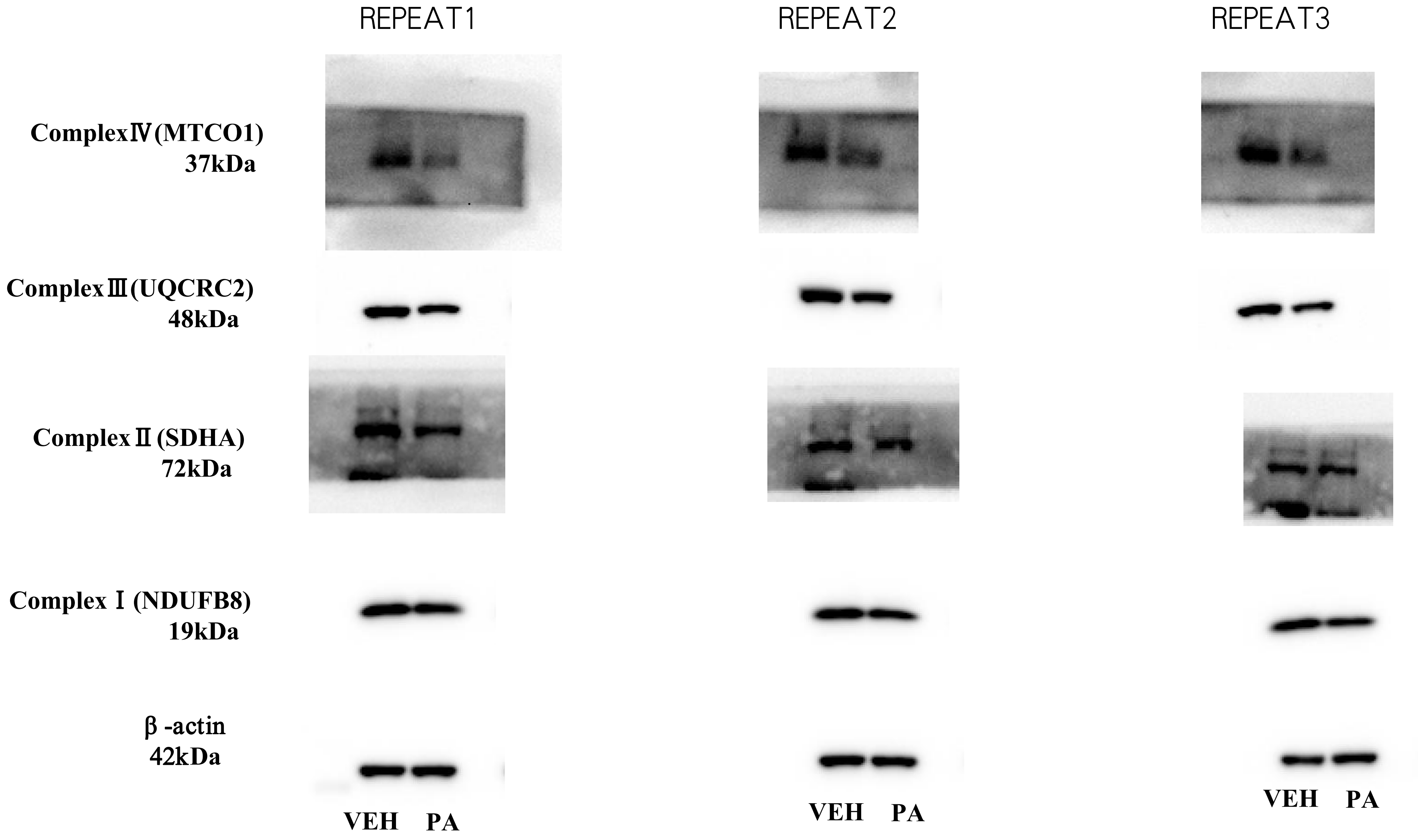
REPEAT1
REPEAT2
REPEAT3
ComplexⅣ(MTCO1)
37kDa
ComplexⅢ(UQCRC2)
48kDa
ComplexⅡ(SDHA)
72kDa
ComplexⅠ(NDUFB8)
19kDa
β-actin
42kDa
VEH
PA
VEH
PA
VEH
PA

Figure3B

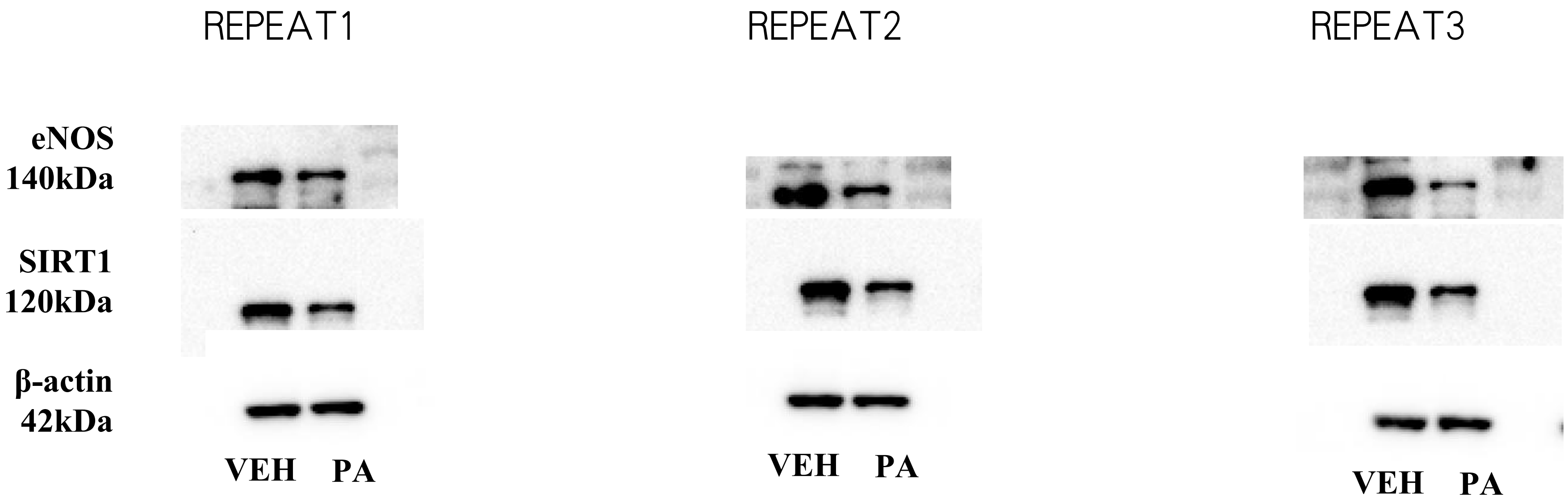

Figure3C

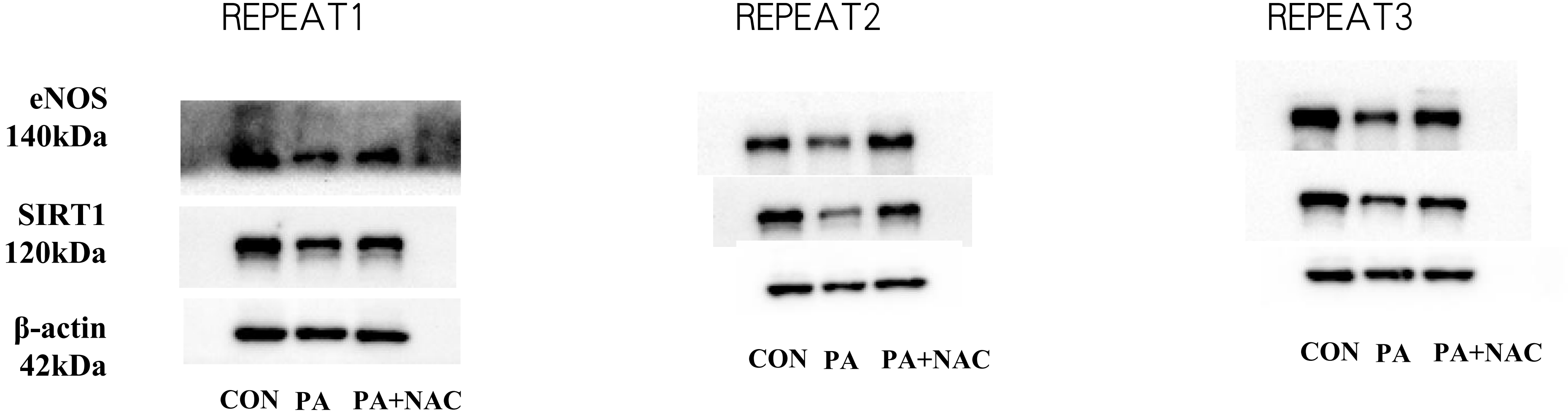

Figure3D

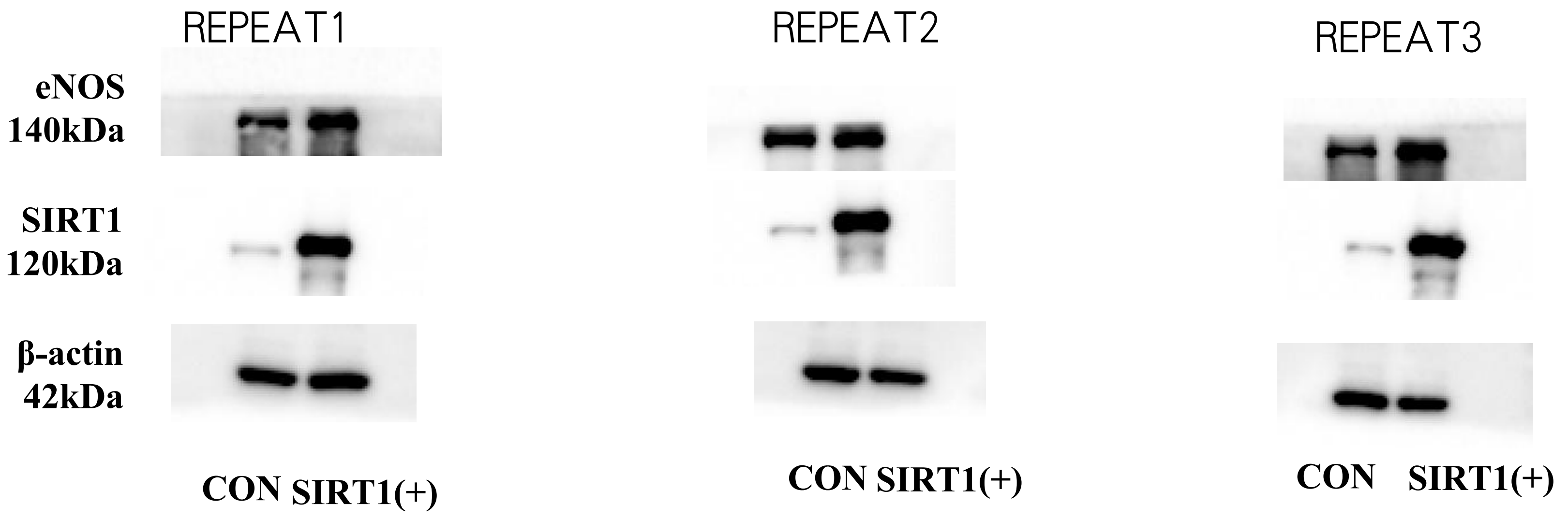

Figure3E

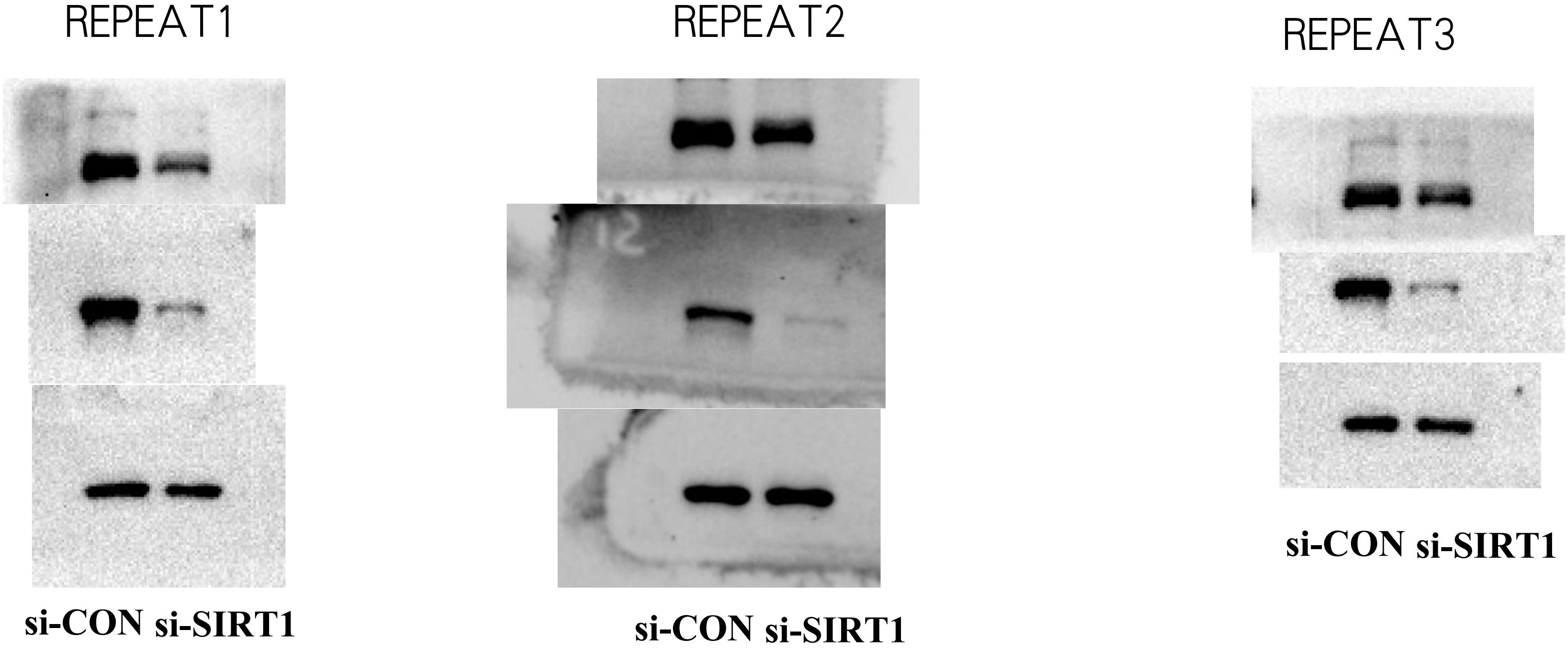

Figure3F

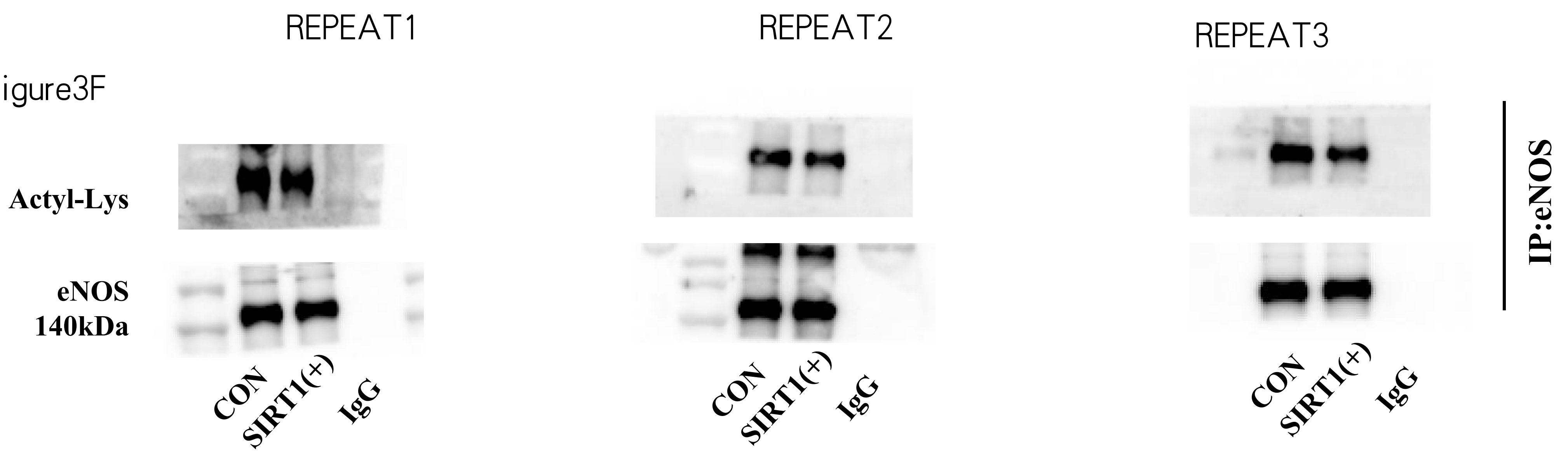

Figure3F

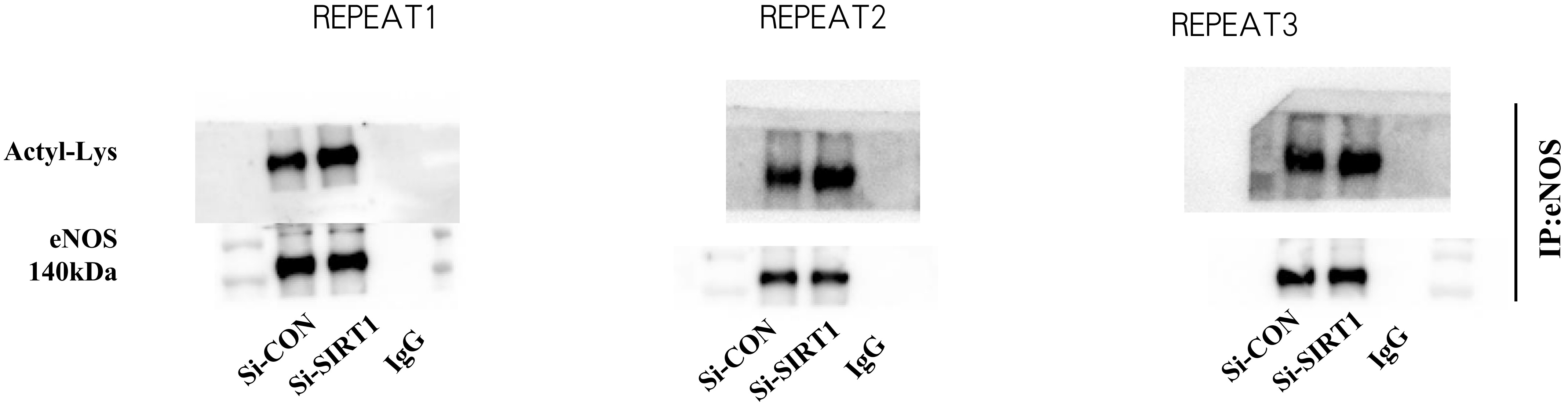

Figure4A21

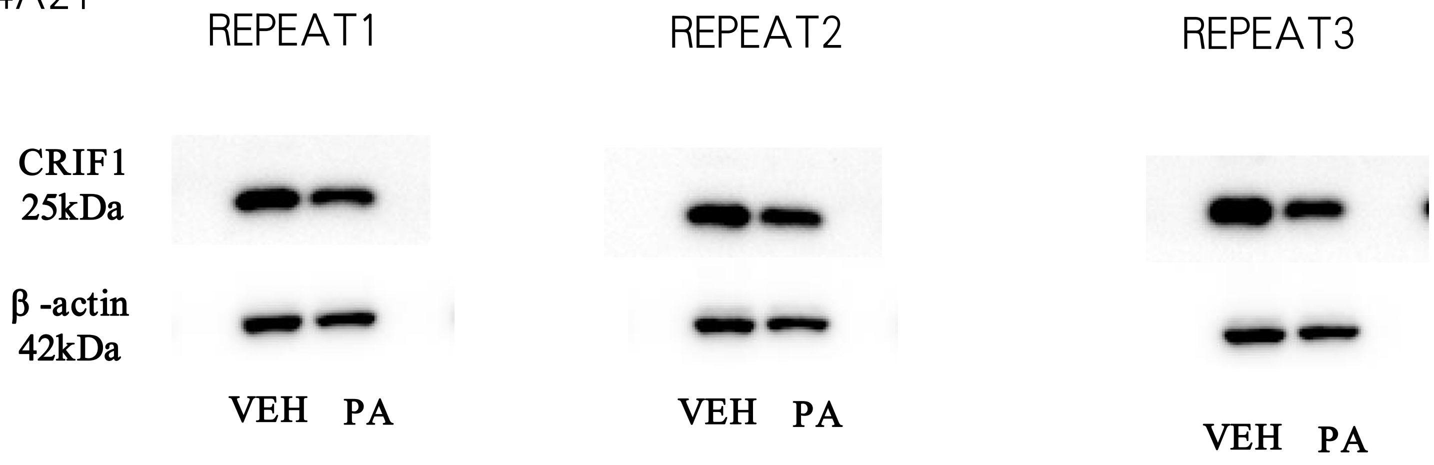

Figure4C

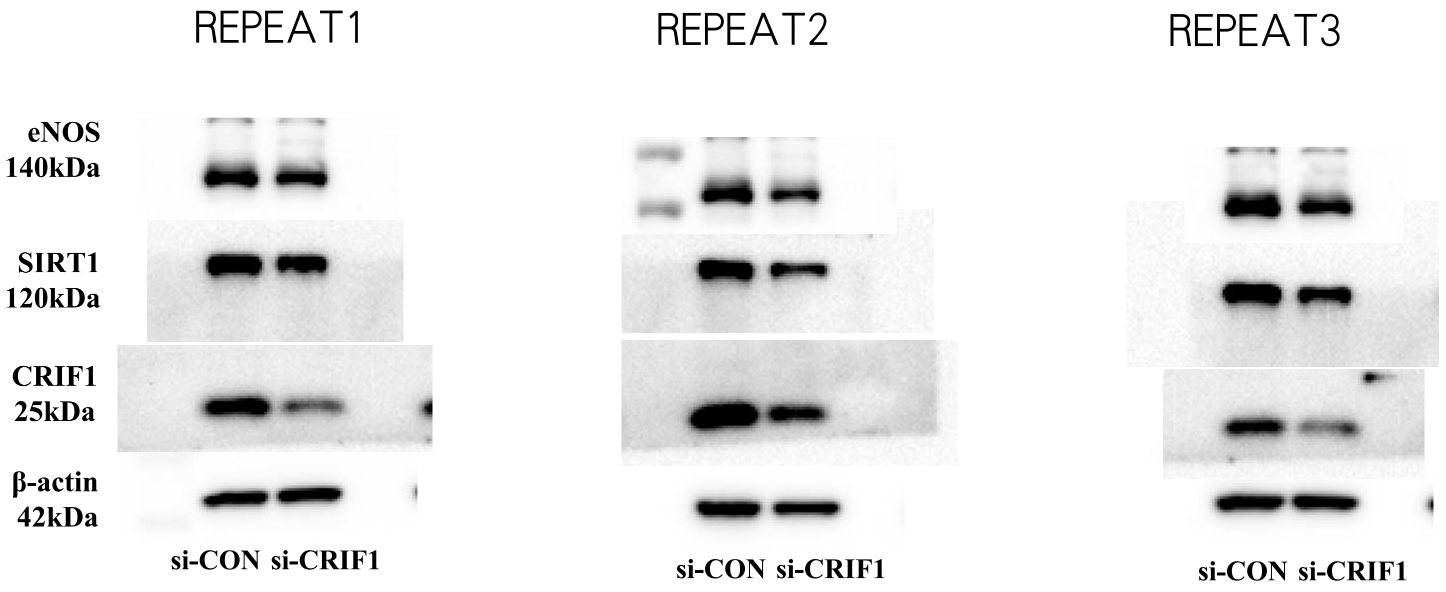

Figure4D

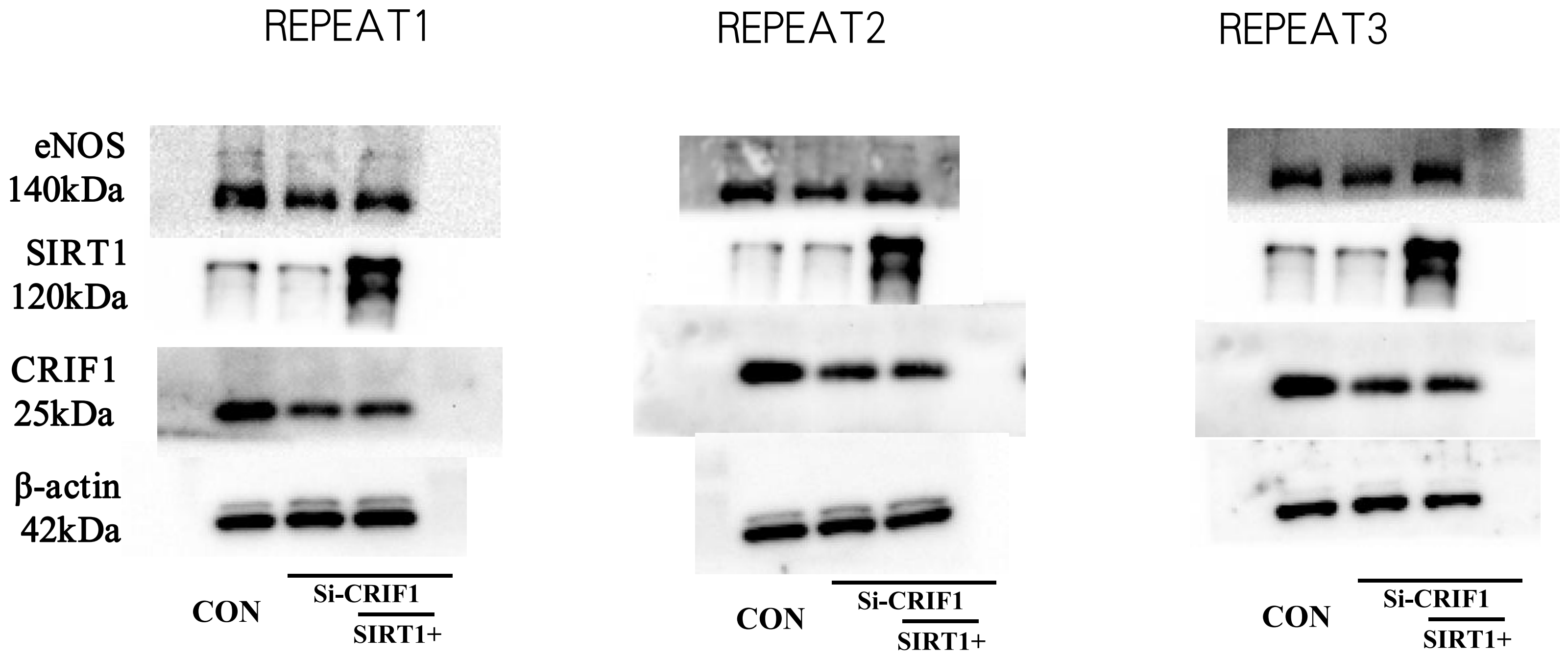
REPEAT1
REPEAT2
REPEAT3
eNOS
140kDa
SIRT1
120kDa
CRIF1
25kDa
β-actin
42kDa
CON
Si-CRIF1
SIRT1+
CON
Si-CRIF1
SIRT1+
CON
Si-CRIF1
SIRT1+

Figure4F

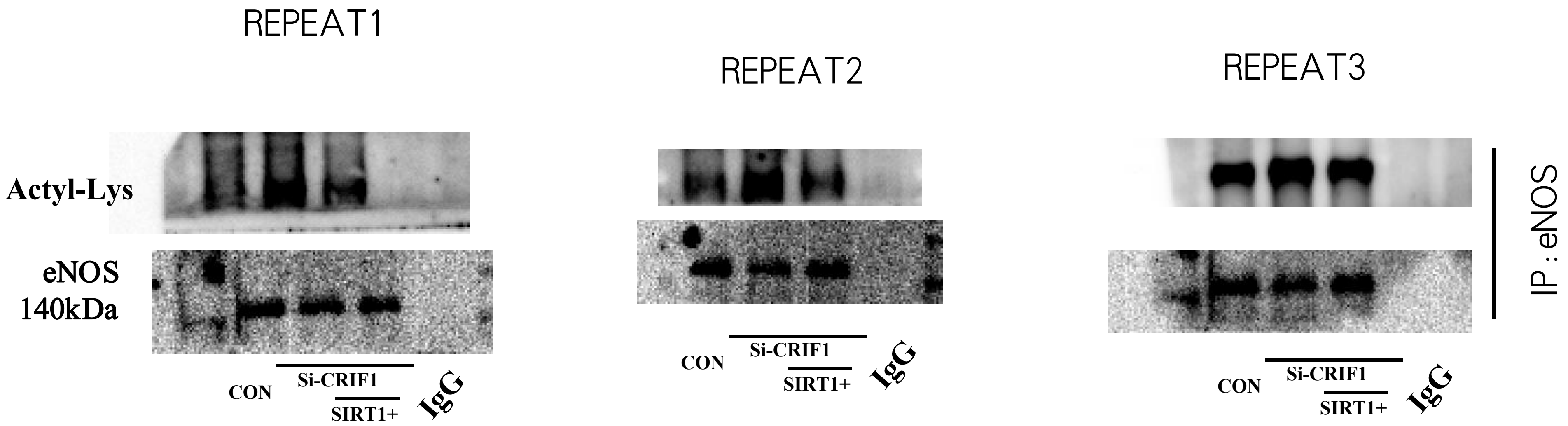
REPEAT1
REPEAT2
REPEAT3
Actyl-Lys
eNOS
140kDa
CON
Si-CRIF1
SIRT1+
IgG
CON
Si-CRIF1
SIRT1+
IgG
CON
Si-CRIF1
SIRT1+
IgG
IP : eNOS

Figure5C

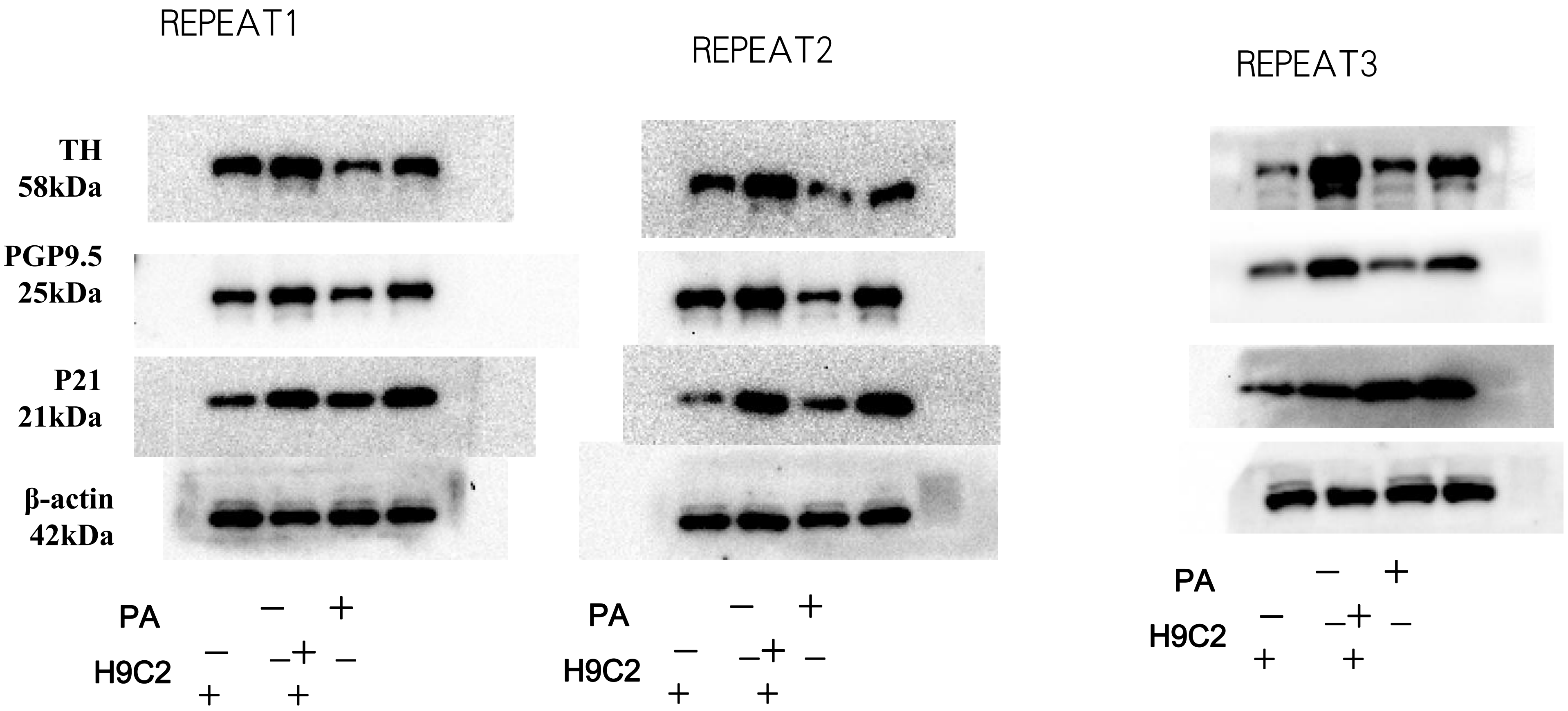
REPEAT1
REPEAT2
REPEAT3
TH
58kDa
PGP9.5
25kDa
P21
21kDa
β-actin
42kDa
PA
H9C2
PA
H9C2
PA
H9C2

Figure6B

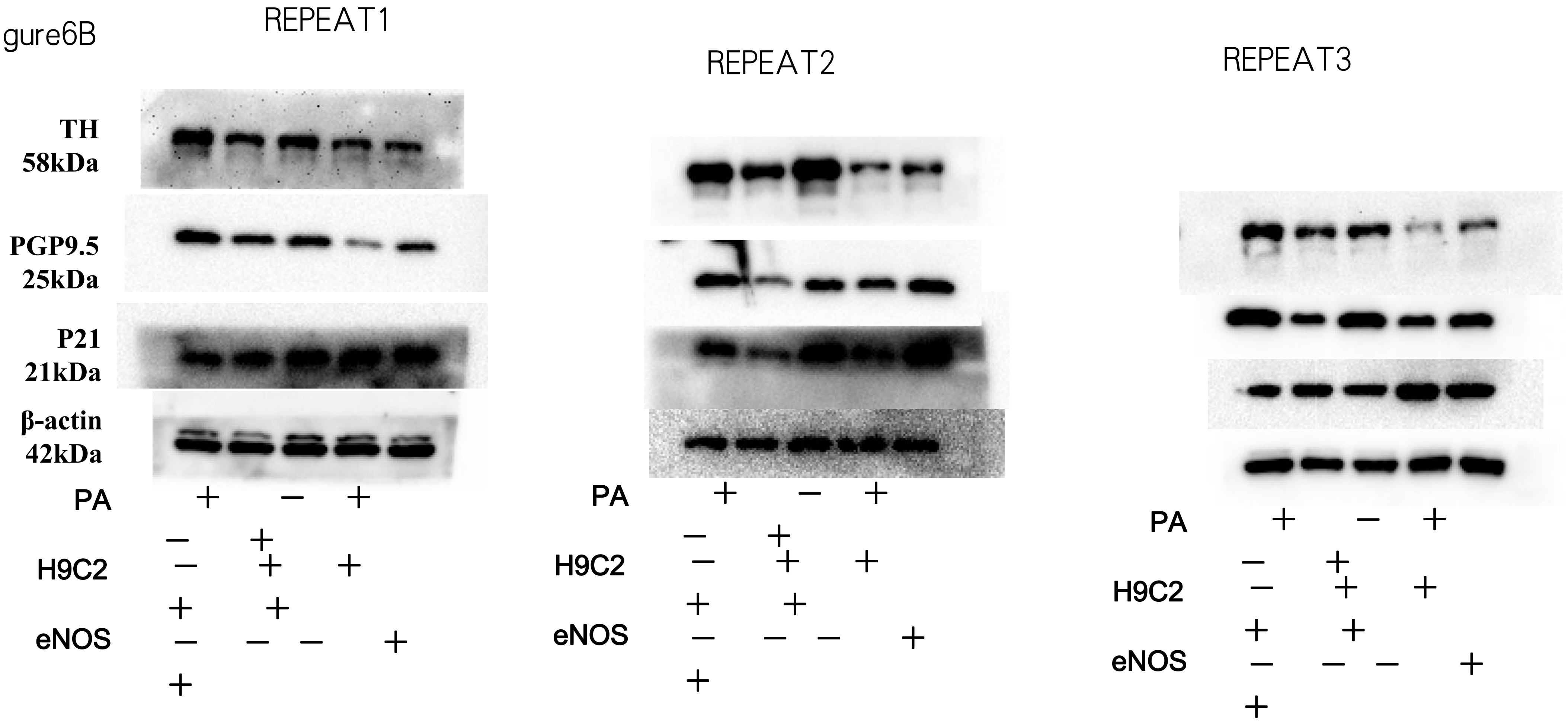
REPEAT1
REPEAT2
REPEAT3
TH
58kDa
PGP9.5
25kDa
P21
21kDa
β-actin
42kDa
PA
H9C2
eNOS
PA
H9C2
eNOS
PA
H9C2
eNOS

Figure6E

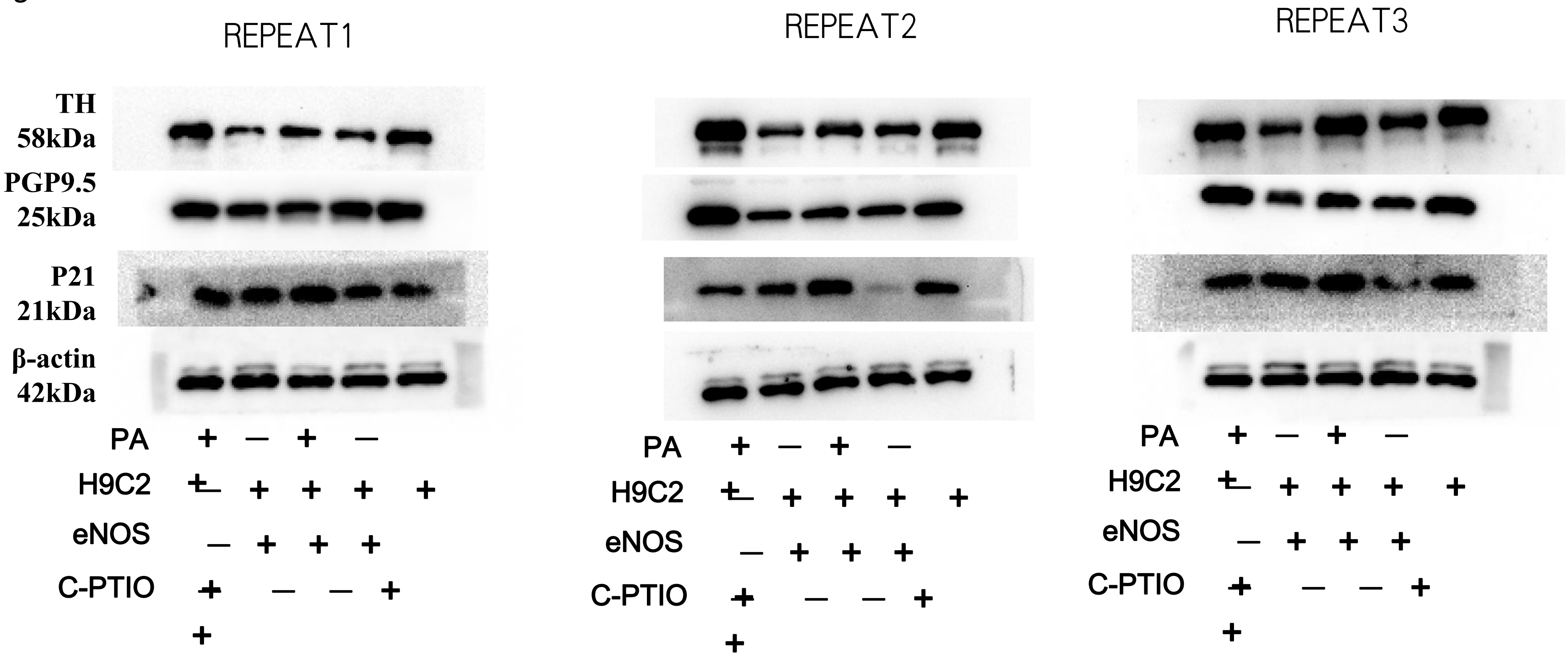
REPEAT1
REPEAT2
REPEAT3
TH
58kDa
PGP9.5
25kDa
P21
21kDa
β-actin
42kDa
PA
H9C2
eNOS
C-PTIO

Figure7B

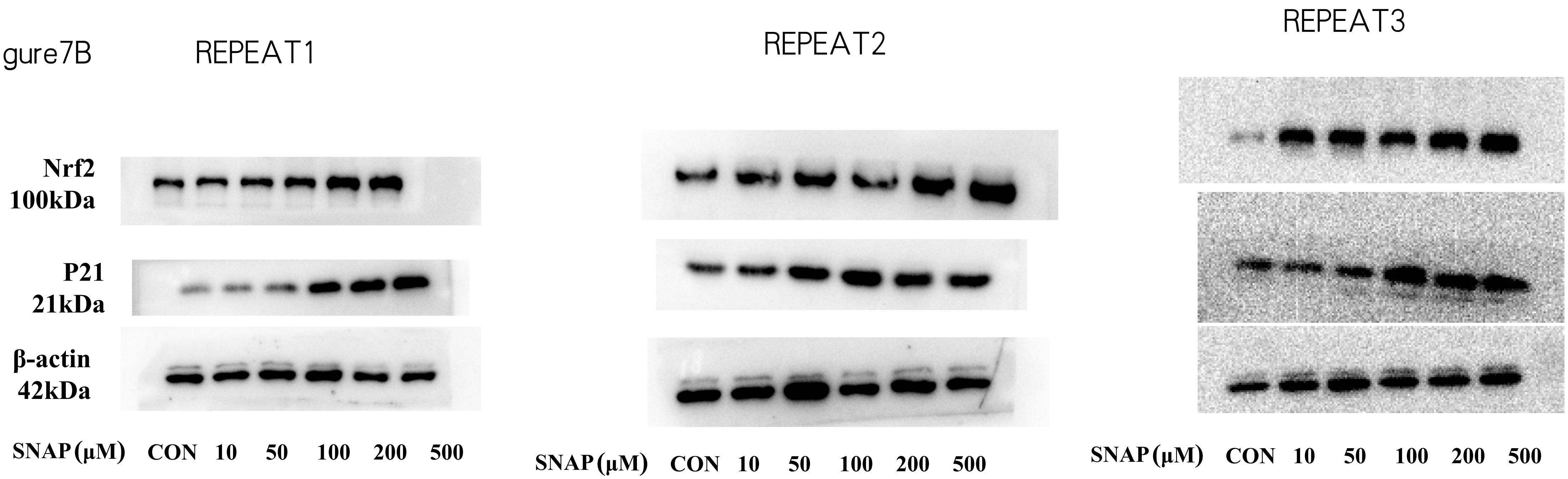

Figure7D

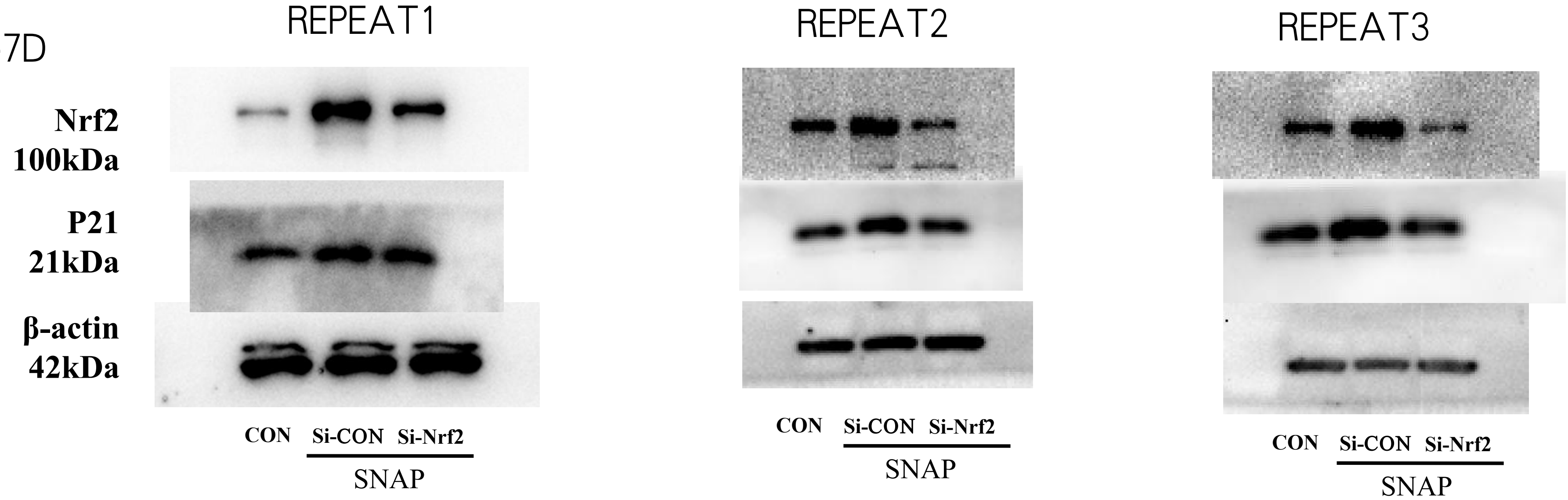

Figure7E

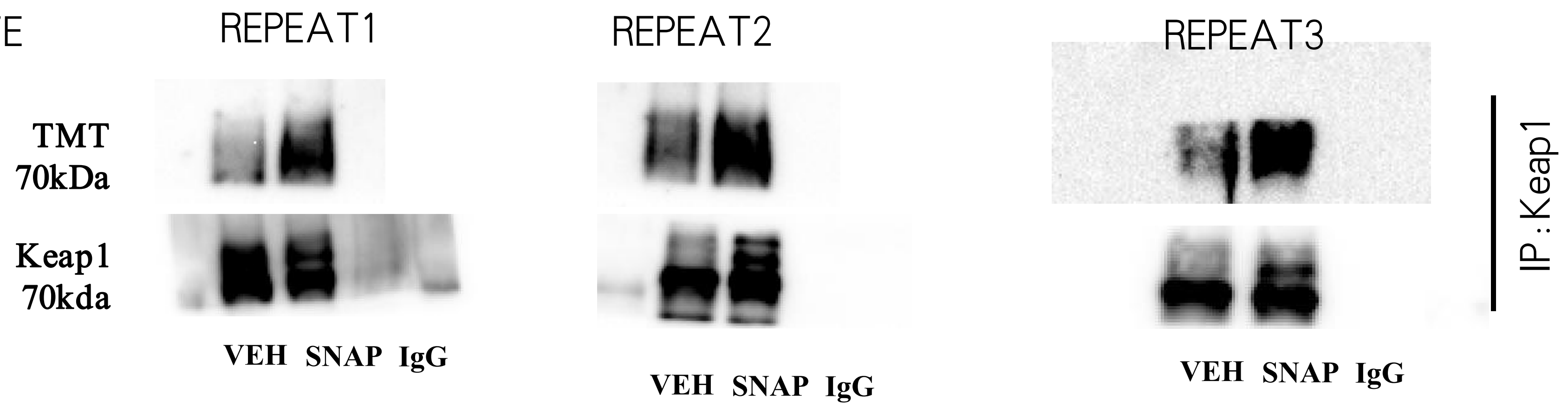

Figure7F

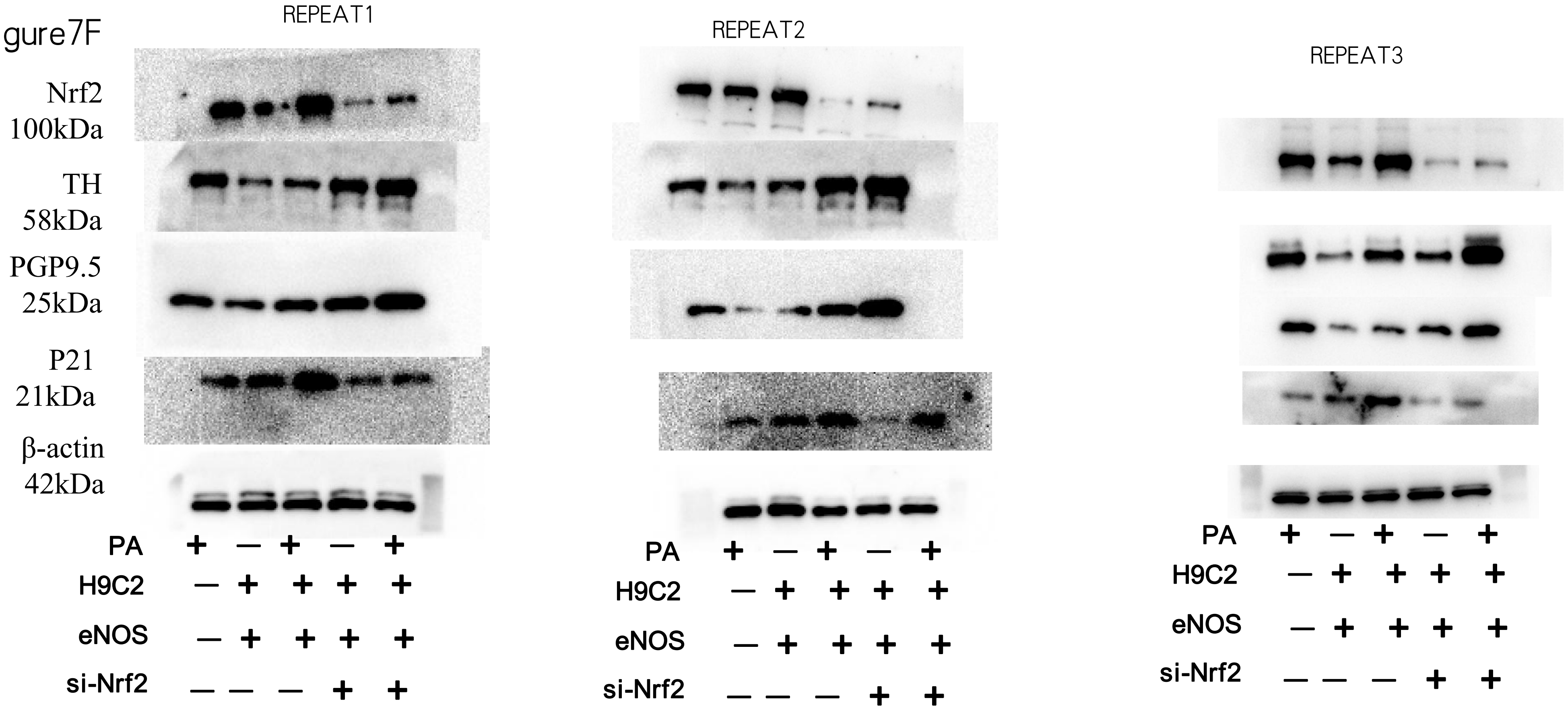

Figure8B

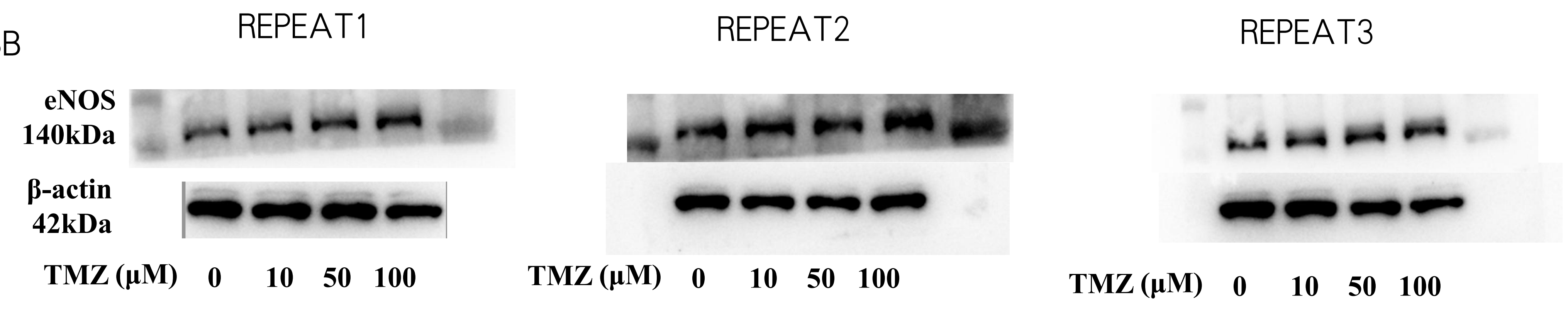

Figure8D

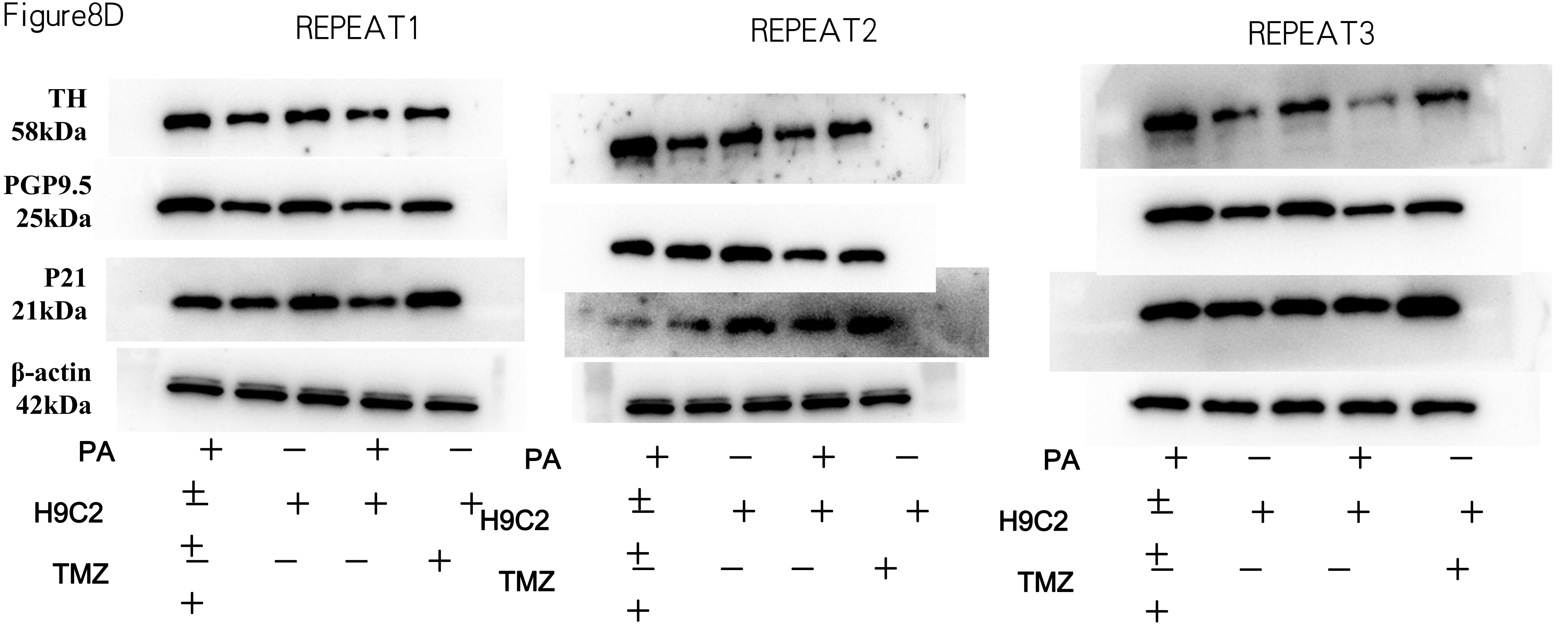

Figure9G

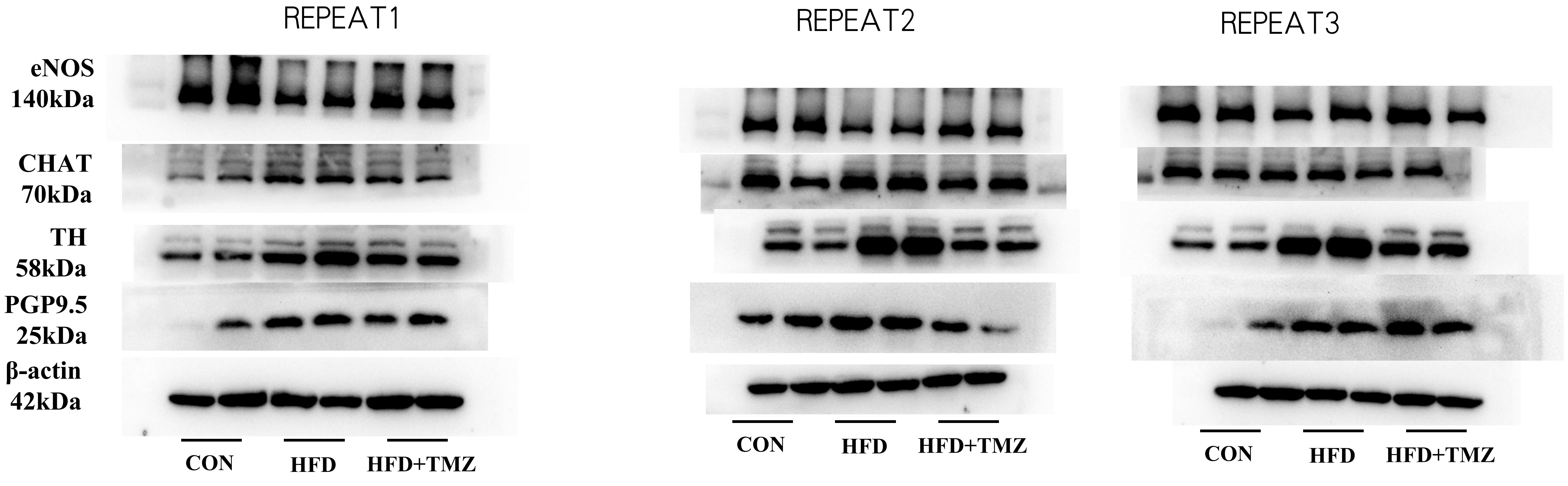
REPEAT1
REPEAT2
REPEAT3
eNOS
140kDa
CHAT
70kDa
TH
58kDa
PGP9.5
25kDa
β-actin
42kDa
CON
HFD
HFD+TMZ
CON
HFD
HFD+TMZ
CON
HFD
HFD+TMZ
